# Supplementary material for: Projecting long-term excess risks of major infectious diseases associated with future extreme weather events in Thailand
Source: PLoS Negl Trop Dis. 2026 Jan 5;20(1):e0013896. doi: 10.1371/journal.pntd.0013896 (PMC12782439; doi:10.1371/journal.pntd.0013896)
Supplement: S1 Table — Cameron and Trivedi test was done on the case counts of each disease respectively to check if the data was overdispersed. Overdispersed data warrants the use of the negative binomial distribution to count data. (DOCX) [file pntd.0013896.s001.docx]

# S1 Table. Overdispersion test.

Cameron and Trivedi test was done on the case counts of each disease respectively to check if the data was overdispersed. Overdispersed data warrants the use of the negative binomial distribution to count data.

| Disease | Lambda t-test value |
| --- | --- |
| Dengue | 26.2* |
| Japanese Encephalitis | 20.2* |
| Influenza | 16.7* |
| Malaria | 11.7* |
| Pneumonia | 117* |
| Leptopspirosis | 10.4* |
| Melioidosis | 18.1* |

*p-value < 0.05
