# Supplementary material for: Projecting long-term excess risks of major infectious diseases associated with future extreme weather events in Thailand
Source: PLoS Negl Trop Dis. 2026 Jan 5;20(1):e0013896. doi: 10.1371/journal.pntd.0013896 (PMC12782439; doi:10.1371/journal.pntd.0013896)
Supplement: S2 Table — Disease-specific generalized additive models were trained taking lagged extreme heat days, lagged standardized precipitation index (SPI), relative humidity and population density as variables. Thin plate splines were used to model non-linear relationships between lagged extreme heat days, lagged SPI and monthly disease case counts. Models including different number of lags were assessed and compared based on the Akaike information criterion (AIC), with lower values indicating better model fit to observations while penalizing for model complexity. The number of variable lags selected for models of each disease are bolded and underlined. (DOCX) [file pntd.0013896.s002.docx]

# S2 Table. Model selection criteria

Disease-specific generalized additive models were trained taking lagged extreme heat days, lagged standardized precipitation index (SPI), relative humidity and population density as variables. Thin plate splines were used to model non-linear relationships between lagged extreme heat days, lagged SPI and monthly disease case counts. Models including different number of lags were assessed and compared based on the Akaike information criterion (AIC), with lower values indicating better model fit to observations while penalizing for model complexity. The number of variable lags selected for models of each disease are bolded and underlined.

| **Dengue** | | | | **Influenza** | | | | **Leptospirosis** | | | |
| --- | --- | --- | --- | --- | --- | --- | --- | --- | --- | --- | --- |
| **Extreme Heat Lags** | **SPI Lags** | | **AIC** | **Extreme Heat Lags** | **SPI Lags** | | **AIC** | **Extreme Heat Lags** | **SPI Lags** | | **AIC** |
| 0 | 0 | | 131217 | 0 | 0 | | 150288 | 0 | 0 | | 65719 |
| 0 | 1 | | 131186 | 0 | 1 | | 150257 | 0 | 1 | | 65716 |
| 0 | 2 | | 131137 | 0 | 2 | | 150185 | 0 | 2 | | 65711 |
| 0 | 3 | | 131023 | 0 | 3 | | 150058 | 0 | 3 | | 65703 |
| 1 | 0 | | 131085 | 1 | 0 | | 150226 | 1 | 0 | | 65716 |
| 1 | 1 | | 131054 | 1 | 1 | | 150203 | 1 | 1 | | 65715 |
| 1 | 2 | | 131006 | 1 | 2 | | 150134 | 1 | 2 | | 65709 |
| 1 | 3 | | 130887 | 1 | 3 | | 150017 | 1 | 3 | | 65702 |
| 2 | 0 | | 130976 | 2 | 0 | | 150224 | 2 | 0 | | 65706 |
| 2 | 1 | | 130947 | 2 | 1 | | 150199 | 2 | 1 | | 65706 |
| 2 | 2 | | 130904 | 2 | 2 | | 150131 | 2 | 2 | | 65702 |
| 2 | 3 | | 130800 | 2 | 3 | | 150008 | 2 | 3 | | 65688 |
| 3 | 0 | | 130831 | 3 | 0 | | 150187 | 3 | 0 | | 65701 |
| 3 | 1 | | 130800 | 3 | 1 | | 150164 | 3 | 1 | | 65700 |
| 3 | 2 | | 130759 | 3 | 2 | | 150094 | 3 | 2 | | 65696 |
| **3** | **3** | | 130676 | **3** | **3** | | 149961 | **3** | **3** | | 65683 |
| **Japanese Encephalitis** | | | | **Malaria** | | | | **Pneumonia** | | | |
| **Extreme Heat Lags** | **SPI Lags** | | **AIC** | **Extreme Heat Lags** | **SPI Lags** | | **AIC** | **Extreme Heat Lags** | **SPI Lags** | | **AIC** |
| 0 | 0 | | 24825 | 0 | 0 | | 86923 | 0 | 0 | | 168283 |
| 0 | 1 | | 24826 | 0 | 1 | | 86915 | 0 | 1 | | 168224 |
| 0 | 2 | | 24822 | 0 | 2 | | 86909 | 0 | 2 | | 168187 |
| 0 | 3 | | 24822 | 0 | 3 | | 86908 | 0 | 3 | | 168128 |
| 1 | 0 | | 24824 | 1 | 0 | | 86923 | 1 | 0 | | 168234 |
| 1 | 1 | | 24825 | 1 | 1 | | 86906 | 1 | 1 | | 168187 |
| 1 | 2 | | 24819 | 1 | 2 | | 86899 | 1 | 2 | | 168161 |
| 1 | 3 | | 24819 | **1** | **3** | | 86898 | 1 | 3 | | 168107 |
| 2 | 0 | | 24820 | 2 | 0 | | 86921 | 2 | 0 | | 168215 |
| 2 | 1 | | 24821 | 2 | 1 | | 86906 | 2 | 1 | | 168164 |
| 2 | 2 | | 24813 | 2 | 2 | | 86903 | 2 | 2 | | 168145 |
| **2** | **3** | | 24811 | 2 | 3 | | 86898 | 2 | 3 | | 168098 |
| 3 | 0 | | 24822 | 3 | 0 | | 86914 | 3 | 0 | | 168206 |
| 3 | 1 | | 24823 | 3 | 1 | | 86905 | 3 | 1 | | 168158 |
| 3 | 2 | | 24815 | 3 | 2 | | 86902 | 3 | 2 | | 168138 |
| 3 | 3 | | 24813 | 3 | 3 | | 86899 | **3** | **3** | | 168093 |
| **Melioidosis** | |  | |  | |  | |  | |  | |
| **Extreme Heat Lags** | **SPI Lags** | | **AIC** |  |  | |  |  |  | |  |
| 0 | 0 | | 49777 |  |  | |  |  |  | |  |
| 0 | 1 | | 49773 |  |  | |  |  |  | |  |
| 0 | 2 | | 49754 |  |  | |  |  |  | |  |
| **0** | **3** | | 49753 |  |  | |  |  |  | |  |
| 1 | 0 | | 49779 |  |  | |  |  |  | |  |
| 1 | 1 | | 49774 |  |  | |  |  |  | |  |
| 1 | 2 | | 49754 |  |  | |  |  |  | |  |
| 1 | 3 | | 49754 |  |  | |  |  |  | |  |
| 2 | 0 | | 49779 |  |  | |  |  |  | |  |
| 2 | 1 | | 49774 |  |  | |  |  |  | |  |
| 2 | 2 | | 49755 |  |  | |  |  |  | |  |
| 2 | 3 | | 49755 |  |  | |  |  |  | |  |
| 3 | 0 | | 49778 |  |  | |  |  |  | |  |
| 3 | 1 | | 49773 |  |  | |  |  |  | |  |
