# Supplementary material for: Projecting long-term excess risks of major infectious diseases associated with future extreme weather events in Thailand
Source: PLoS Negl Trop Dis. 2026 Jan 5;20(1):e0013896. doi: 10.1371/journal.pntd.0013896 (PMC12782439; doi:10.1371/journal.pntd.0013896)
Supplement: S3 Table — Disease-specific generalized additive models (GAM) and generalised linear models (GLM) were trained with relative humidity, lagged extreme heat days, lagged standardized precipitation index (SPI) and population density as variables. The Akaike information criterion (AIC) of each model was calculated to compare model fit of GLM against GAM. (DOCX) [file pntd.0013896.s003.docx]

# S3 Table. AIC of the GAM and GLM models for each disease.

Disease-specific generalized additive models (GAM) and generalised linear models (GLM) were trained with relative humidity, lagged extreme heat days, lagged standardized precipitation index (SPI) and population density as variables. The Akaike information criterion (AIC) of each model was calculated to compare model fit of GLM against GAM.

|  | **Dengue** | **JEV** | **Influenza** | **Malaria** | **Pneumonia** | **Leptospirosis** | **Melioidosis** |
| --- | --- | --- | --- | --- | --- | --- | --- |
| **GAM AIC** | 105367 | 19009 | 113986 | 77945 | 134751 | 54598 | 38874 |
| **GLM AIC** | 107083 | 19083 | 115407 | 78419 | 137366 | 54931 | 39034 |
