# Supplementary material for: Projecting long-term excess risks of major infectious diseases associated with future extreme weather events in Thailand
Source: PLoS Negl Trop Dis. 2026 Jan 5;20(1):e0013896. doi: 10.1371/journal.pntd.0013896 (PMC12782439; doi:10.1371/journal.pntd.0013896)
Supplement: S4 Table — Disease-specific generalized additive models (GAM) were trained with and without relative humidity (RH) respectively, together with lagged extreme heat days, lagged standardized precipitation index (SPI) and population density as variables. The lags chosen for extreme heat days and SPI are indicated in Supplementary Table S2. Thin plate splines were used to model non-linear relationships between lagged extreme heat days, lagged SPI and monthly disease case counts. The Akaike information criterion (AIC) of each model was calculated to compare model fit with inclusion of relative humidity as a predictor. AIC is used to assess model fit as it balances goodness of fit with model complexity. Lower AIC values which indicated better model fit are bolded. (DOCX) [file pntd.0013896.s004.docx]

# S4 Table. AIC of each model with and without relative humidity as a predictor.

Disease-specific generalized additive models (GAM) were trained with and without relative humidity (RH) respectively, together with lagged extreme heat days, lagged standardized precipitation index (SPI) and population density as variables. The lags chosen for extreme heat days and SPI are indicated in Supplementary Table S2. Thin plate splines were used to model non-linear relationships between lagged extreme heat days, lagged SPI and monthly disease case counts. The Akaike information criterion (AIC) of each model was calculated to compare model fit with inclusion of relative humidity as a predictor. AIC is used to assess model fit as it balances goodness of fit with model complexity. Lower AIC values which indicated better model fit are bolded.

|  | **Dengue** | **JEV** | **Influenza** | **Malaria** | **Pneumonia** | **Leptospirosis** | **Melioidosis** |
| --- | --- | --- | --- | --- | --- | --- | --- |
| **With RH** | **105367** | **19009** | **113986** | **77945** | **134751** | **54598** | **38874** |
| **No RH** | 111183 | 21470 | 119713 | 81657 | 144780 | 57204 | 40493 |
