# Supplementary material for: Projecting long-term excess risks of major infectious diseases associated with future extreme weather events in Thailand
Source: PLoS Negl Trop Dis. 2026 Jan 5;20(1):e0013896. doi: 10.1371/journal.pntd.0013896 (PMC12782439; doi:10.1371/journal.pntd.0013896)
Supplement: S5 Table — Disease-specific generalized additive models (GAM) were trained with lagged extreme heat days, lagged standardized precipitation index (SPI), relative humidity and population density as variables. The lags chosen for extreme heat days and SPI are indicated in Supplementary Table S2. Splines were used to model non-linear relationships between lagged extreme heat days, lagged SPI and monthly disease case counts. We explored whether using different splines would affect model fit. Province and disease-specific generalized additive models (GAM) were trained with thin plate regression splines (TR) and compared with GAM models using the same predictors and cubic regression splines (CR). The Akaike information criterion (AIC) of each model was calculated to compare model fit using different splines. AIC is used to assess model fit as it balances goodness of fit with model complexity. Lower AIC values which indicated better model fit are bolded. (DOCX) [file pntd.0013896.s005.docx]

# S5 Table. AIC of GAM models using thin plate regression spline and cubic regression spline.

Disease-specific generalized additive models (GAM) were trained with lagged extreme heat days, lagged standardized precipitation index (SPI), relative humidity and population density as variables. The lags chosen for extreme heat days and SPI are indicated in Supplementary Table S2. Splines were used to model non-linear relationships between lagged extreme heat days, lagged SPI and monthly disease case counts. We explored whether using different splines would affect model fit. Province and disease-specific generalized additive models (GAM) were trained with thin plate regression splines (TR) and compared with GAM models using the same predictors and cubic regression splines (CR). The Akaike information criterion (AIC) of each model was calculated to compare model fit using different splines. AIC is used to assess model fit as it balances goodness of fit with model complexity. Lower AIC values which indicated better model fit are bolded.

|  | **Dengue** | **JEV** | **Influenza** | **Malaria** | **Pneumonia** | **Leptospirosis** | **Melioidosis** |
| --- | --- | --- | --- | --- | --- | --- | --- |
| **TR**  **spline** | 105367 | 19009 | 113986 | 77945 | 134751 | 54598 | 38874 |
| **CR spline** | 105367 | 19009 | 113986 | 77945 | 134751 | 54598 | 38874 |
