# Supplementary material for: Projecting long-term excess risks of major infectious diseases associated with future extreme weather events in Thailand
Source: PLoS Negl Trop Dis. 2026 Jan 5;20(1):e0013896. doi: 10.1371/journal.pntd.0013896 (PMC12782439; doi:10.1371/journal.pntd.0013896)
Supplement: S6 Table — Disease-specific generalized additive models (GAM) were trained on historical data from 2003-2019 and used to project future case counts of the respective disease across 4 time periods (2021–2040, 2041–2060, 2061–2080, 2081–2100) and 4 climate change scenarios (SSP126, SSP245, SSP370, SSP585) during periods of extreme weather. National-level excess risk was calculated using the mean disease case counts across the historical period and the projected case counts at a respective time period and climate change scenario. Excess risk represents the percentage change in disease cases compared to historical levels. Values in asterisk are statistically significant. (DOCX) [file pntd.0013896.s006.docx]

# S6 Table. Annual national projected excess risk of each disease attributable to overall extreme weather, extreme heat, extreme dry and extreme wet weather across time periods and climate change scenarios.

Disease-specific generalized additive models (GAM) were trained on historical data from 2003-2019 and used to project future case counts of the respective disease across 4 time periods (2021-2040, 2041-2060, 2061-2080, 2081-2100) and 4 climate change scenarios (SSP126, SSP245, SSP370, SSP585) during periods of extreme weather. National-level excess risk was calculated using the mean disease case counts across the historical period and the projected case counts at a respective time period and climate change scenario. Excess risk represents the percentage change in disease cases compared to historical levels. Values in asterisk are statistically significant.

| **Period** | **SSP** | **Disease** | **Overall ER (%)** | **Overall ER 95% CI (%)** | **Extreme Heat ER (%)** | **Extreme Heat 95% CI (%)** | **Extreme Dry ER (%)** | **Extreme Dry 95% CI (%)** | **Extreme Wet ER (%)** | **Extreme Wet 95% CI (%)** |
| --- | --- | --- | --- | --- | --- | --- | --- | --- | --- | --- |
| 2021-2040 | SSP126 | Dengue | -9.93 | (-27.48, 7.62) | -2.57 | (-21.47, 16.33) | -42.78* | (-54.43, -31.13) | -46.86* | (-58.02, -35.69) |
| 2021-2040 | SSP245 | Dengue | 16.1 | (-7.3, 39.5) | 26.29* | (0.9, 51.68) | -25.41* | (-41.05, -9.77) | -34.96* | (-48.68, -21.25) |
| 2021-2040 | SSP370 | Dengue | -18.61* | (-34.97, -2.25) | -12.43 | (-30.01, 5.16) | -45.72* | (-56.82, -34.61) | -46.03* | (-57.39, -34.66) |
| 2021-2040 | SSP585 | Dengue | -14.15 | (-31.34, 3.03) | -8.24 | (-26.6, 10.11) | -29.54* | (-44.09, -14.99) | -48.24* | (-58.98, -37.49) |
| 2041-2060 | SSP126 | Dengue | -9.09 | (-26.95, 8.78) | -2.14 | (-21.29, 17) | -35.66* | (-49.44, -21.89) | -47.32* | (-58.23, -36.41) |
| 2041-2060 | SSP245 | Dengue | -0.67 | (-21.2, 19.87) | 6.55 | (-15.48, 28.58) | -28.85* | (-44.51, -13.19) | -39.36* | (-51.92, -26.8) |
| 2041-2060 | SSP370 | Dengue | -14.23 | (-31.99, 3.54) | -8.83 | (-27.66, 10.01) | -45.13* | (-57.05, -33.22) | -52.92* | (-62.82, -43.01) |
| 2041-2060 | SSP585 | Dengue | -18* | (-35.41, -0.59) | -12.86 | (-31.39, 5.67) | -48.97* | (-59.76, -38.18) | -52.77* | (-63.02, -42.52) |
| 2061-2080 | SSP126 | Dengue | -19.9* | (-35.55, -4.25) | -17.89* | (-33.89, -1.89) | -38.47* | (-51.47, -25.47) | -42.04* | (-54.71, -29.36) |
| 2061-2080 | SSP245 | Dengue | -24.86* | (-40.04, -9.68) | -23.75* | (-39.12, -8.37) | -39.73* | (-53.24, -26.21) | -38.1* | (-51.71, -24.5) |
| 2061-2080 | SSP370 | Dengue | -25.51* | (-40.86, -10.15) | -24.2* | (-39.8, -8.59) | -38.99* | (-52.29, -25.69) | -42.81* | (-55.92, -29.71) |
| 2061-2080 | SSP585 | Dengue | -23.03* | (-39.83, -6.24) | -22.14* | (-39.13, -5.15) | -45.51* | (-57.64, -33.38) | -41.09* | (-55.14, -27.04) |
| 2081-2100 | SSP126 | Dengue | -9.26 | (-27.02, 8.5) | -2.51 | (-21.49, 16.47) | -36.34* | (-49.43, -23.25) | -47.75* | (-58.6, -36.9) |
| 2081-2100 | SSP245 | Dengue | -15.12 | (-32.59, 2.35) | -10.34 | (-28.76, 8.08) | -52.05* | (-62.48, -41.63) | -48.56* | (-59.29, -37.84) |
| 2081-2100 | SSP370 | Dengue | -23.27* | (-41.73, -4.82) | -20.91* | (-40.03, -1.78) | -52.09* | (-63.16, -41.03) | -46.16* | (-58.05, -34.27) |
| 2081-2100 | SSP585 | Dengue | -22.86* | (-42.16, -3.57) | -22.04* | (-41.58, -2.5) | -41.89* | (-55.59, -28.19) | -46.2* | (-59.41, -32.99) |
| 2021-2040 | SSP126 | Influenza | -5.66 | (-23.59, 12.26) | -6.05 | (-23.77, 11.67) | -9.06 | (-27.13, 9) | -14.92 | (-31.88, 2.04) |
| 2021-2040 | SSP245 | Influenza | 7.95 | (-13.36, 29.25) | 9.74 | (-11.84, 31.33) | 17.03 | (-6.86, 40.91) | -13.51 | (-30.9, 3.88) |
| 2021-2040 | SSP370 | Influenza | -5.7 | (-23.74, 12.34) | -5.95 | (-23.83, 11.93) | 1.19 | (-18.91, 21.28) | -19.5* | (-36.16, -2.83) |
| 2021-2040 | SSP585 | Influenza | -6.72 | (-24.67, 11.23) | -6.41 | (-24.37, 11.55) | -5.52 | (-23.84, 12.79) | -13.45 | (-30.52, 3.63) |
| 2041-2060 | SSP126 | Influenza | 0.72 | (-18.67, 20.1) | 0.9 | (-18.46, 20.27) | 14.86 | (-7.49, 37.21) | -13.61 | (-30.88, 3.67) |
| 2041-2060 | SSP245 | Influenza | 2.58 | (-18.37, 23.52) | 3.98 | (-17.32, 25.29) | -3.39 | (-22.45, 15.67) | -13.17 | (-30.56, 4.21) |
| 2041-2060 | SSP370 | Influenza | -4.16 | (-23.4, 15.08) | -4.09 | (-23.34, 15.15) | 10.09 | (-12.09, 32.26) | -19.61* | (-35.94, -3.28) |
| 2041-2060 | SSP585 | Influenza | 0.52 | (-20.35, 21.39) | 1.47 | (-19.71, 22.64) | -1.75 | (-21.04, 17.55) | -13.31 | (-30.49, 3.86) |
| 2061-2080 | SSP126 | Influenza | -5.65 | (-23.7, 12.41) | -5.05 | (-23.16, 13.06) | -7.49 | (-25.6, 10.62) | -23.98* | (-39.47, -8.48) |
| 2061-2080 | SSP245 | Influenza | -11.07 | (-28.59, 6.44) | -10.75 | (-28.29, 6.79) | -18.88* | (-35.43, -2.33) | -31.12* | (-45.23, -17.01) |
| 2061-2080 | SSP370 | Influenza | -11.51 | (-29.15, 6.12) | -11.33 | (-28.98, 6.33) | -17.54* | (-34.2, -0.89) | -37.77* | (-50.78, -24.77) |
| 2061-2080 | SSP585 | Influenza | -8.41 | (-27.87, 11.05) | -8.37 | (-27.86, 11.12) | -13.92 | (-31.23, 3.39) | -30.2* | (-44.5, -15.9) |
| 2081-2100 | SSP126 | Influenza | -4.61 | (-22.97, 13.75) | -4.36 | (-22.73, 14.01) | -0.33 | (-19.62, 18.95) | -13.31 | (-30.49, 3.88) |
| 2081-2100 | SSP245 | Influenza | -3.32 | (-22.64, 15.99) | -2.79 | (-22.25, 16.68) | -18.54* | (-34.89, -2.19) | -3.6 | (-23.08, 15.89) |
| 2081-2100 | SSP370 | Influenza | -11.17 | (-31.63, 9.28) | -10.58 | (-31.31, 10.16) | -20.14* | (-36.51, -3.77) | -30.29* | (-44.45, -16.12) |
| 2081-2100 | SSP585 | Influenza | -13.28 | (-35, 8.44) | -13.21 | (-35.07, 8.65) | -17.02* | (-33.69, -0.35) | -35.08* | (-49.33, -20.84) |
| 2021-2040 | SSP126 | Japanese Encephalitis | 4.52 | (-23.58, 32.62) | 5.91 | (-22.34, 34.17) | -9.3 | (-35.63, 17.03) | -10.67 | (-38.24, 16.9) |
| 2021-2040 | SSP245 | Japanese Encephalitis | 15.53 | (-16.17, 47.24) | 17.54 | (-14.48, 49.58) | 2.99 | (-28.01, 33.98) | -9.63 | (-38.93, 19.67) |
| 2021-2040 | SSP370 | Japanese Encephalitis | 3.95 | (-24.9, 32.81) | 5.48 | (-23.61, 34.58) | -10.16 | (-37.62, 17.3) | -12.42 | (-38.36, 13.51) |
| 2021-2040 | SSP585 | Japanese Encephalitis | 5.4 | (-23.69, 34.49) | 6.87 | (-22.52, 36.27) | -6.25 | (-32.15, 19.65) | -11.13 | (-38.87, 16.61) |
| 2041-2060 | SSP126 | Japanese Encephalitis | 6.33 | (-22.67, 35.33) | 7.61 | (-21.44, 36.67) | -6.05 | (-33.66, 21.56) | -9.36 | (-38.94, 20.21) |
| 2041-2060 | SSP245 | Japanese Encephalitis | 7.29 | (-23.33, 37.91) | 8.69 | (-22.11, 39.49) | -3.04 | (-32.86, 26.77) | -5.93 | (-36.09, 24.23) |
| 2041-2060 | SSP370 | Japanese Encephalitis | 3.41 | (-25.86, 32.69) | 4.65 | (-24.87, 34.17) | -8.73 | (-36.18, 18.73) | -16.58 | (-41.68, 8.52) |
| 2041-2060 | SSP585 | Japanese Encephalitis | 6.64 | (-24.27, 37.55) | 7.94 | (-23.27, 39.15) | -17.5 | (-40.51, 5.51) | -5.7 | (-35.89, 24.5) |
| 2061-2080 | SSP126 | Japanese Encephalitis | -2.64 | (-28.44, 23.17) | -2.18 | (-28.03, 23.67) | -10.15 | (-35.16, 14.85) | -12.25 | (-38.07, 13.58) |
| 2061-2080 | SSP245 | Japanese Encephalitis | -5.97 | (-32.02, 20.09) | -5.66 | (-31.73, 20.42) | -14.89 | (-39.38, 9.61) | -7.15 | (-35.39, 21.08) |
| 2061-2080 | SSP370 | Japanese Encephalitis | -4.58 | (-31.24, 22.08) | -4.29 | (-30.98, 22.4) | -11.73 | (-38, 14.54) | -14.72 | (-41.63, 12.2) |
| 2061-2080 | SSP585 | Japanese Encephalitis | 0.63 | (-29.07, 30.33) | 0.78 | (-28.99, 30.55) | -8.2 | (-34.79, 18.39) | -9.01 | (-37.1, 19.08) |
| 2081-2100 | SSP126 | Japanese Encephalitis | 7.99 | (-21.26, 37.24) | 9.29 | (-20.12, 38.7) | -8.24 | (-34.14, 17.67) | -8.34 | (-37.46, 20.77) |
| 2081-2100 | SSP245 | Japanese Encephalitis | 7.05 | (-23.33, 37.44) | 8.35 | (-22.46, 39.16) | -18.23 | (-43.42, 6.96) | -3.61 | (-29.46, 22.23) |
| 2081-2100 | SSP370 | Japanese Encephalitis | 4.59 | (-26.84, 36.02) | 5.28 | (-26.31, 36.88) | -12.6 | (-39.88, 14.68) | -18.3 | (-43.22, 6.61) |
| 2081-2100 | SSP585 | Japanese Encephalitis | 4.66 | (-27.15, 36.47) | 4.97 | (-27, 36.95) | -8.75 | (-35.03, 17.54) | -6.94 | (-36.66, 22.78) |
| 2021-2040 | SSP126 | Leptospirosis | -2.55 | (-41.64, 36.54) | -3.9 | (-42.08, 34.27) | -4.98 | (-42.79, 32.83) | 16.23 | (-37.15, 69.61) |
| 2021-2040 | SSP245 | Leptospirosis | 0.31 | (-41.7, 42.33) | -0.99 | (-42.21, 40.24) | -4.28 | (-43.83, 35.27) | 24.67 | (-32.27, 81.61) |
| 2021-2040 | SSP370 | Leptospirosis | 0.72 | (-40.19, 41.63) | -0.3 | (-40.56, 39.96) | -2.99 | (-41.38, 35.39) | 21.98 | (-33.32, 77.27) |
| 2021-2040 | SSP585 | Leptospirosis | 0.38 | (-40.62, 41.39) | -0.85 | (-41.05, 39.35) | -5.59 | (-42.99, 31.82) | 21.52 | (-34.22, 77.26) |
| 2041-2060 | SSP126 | Leptospirosis | -0.59 | (-41.23, 40.04) | -3.12 | (-42.35, 36.11) | 0.72 | (-38.61, 40.05) | 24.45 | (-31.75, 80.66) |
| 2041-2060 | SSP245 | Leptospirosis | 2.29 | (-42, 46.59) | 0.66 | (-43.12, 44.45) | -1.51 | (-41.17, 38.14) | 24.12 | (-29.84, 78.07) |
| 2041-2060 | SSP370 | Leptospirosis | 3.25 | (-40.62, 47.11) | 2.48 | (-40.98, 45.94) | -1.87 | (-40.58, 36.84) | 21.61 | (-33.97, 77.19) |
| 2041-2060 | SSP585 | Leptospirosis | 11.93 | (-38.22, 62.07) | 11.51 | (-38.56, 61.59) | -2.08 | (-40.63, 36.47) | 25.39 | (-32.06, 82.85) |
| 2061-2080 | SSP126 | Leptospirosis | 0.74 | (-40.69, 42.16) | -0.42 | (-40.99, 40.15) | -7.56 | (-43.91, 28.78) | 14.16 | (-39.53, 67.85) |
| 2061-2080 | SSP245 | Leptospirosis | -1.03 | (-43.07, 41.01) | -1.77 | (-43.34, 39.8) | -12.09 | (-48.26, 24.07) | 11.26 | (-40.54, 63.06) |
| 2061-2080 | SSP370 | Leptospirosis | 1.65 | (-41.58, 44.88) | 1.53 | (-41.61, 44.66) | -12.71 | (-47.44, 22.02) | 7.53 | (-42.75, 57.82) |
| 2061-2080 | SSP585 | Leptospirosis | 17.47 | (-36.95, 71.89) | 17.65 | (-36.94, 72.23) | -11.13 | (-46.46, 24.21) | 1.87 | (-44.59, 48.32) |
| 2081-2100 | SSP126 | Leptospirosis | -1 | (-41.17, 39.17) | -3.27 | (-42.15, 35.62) | 5.72 | (-35.8, 47.24) | 23.83 | (-31.95, 79.61) |
| 2081-2100 | SSP245 | Leptospirosis | 3.89 | (-40.59, 48.37) | 3.26 | (-40.96, 47.49) | -2.52 | (-42.12, 37.08) | 21.47 | (-31.85, 74.8) |
| 2081-2100 | SSP370 | Leptospirosis | 21.38 | (-38.69, 81.44) | 22.68 | (-38.55, 83.91) | -3.69 | (-42.04, 34.66) | 8.24 | (-41.48, 57.96) |
| 2081-2100 | SSP585 | Leptospirosis | 27.44 | (-39.35, 94.22) | 27.78 | (-39.36, 94.91) | -9.58 | (-45.7, 26.54) | 3.69 | (-42.84, 50.22) |
| 2021-2040 | SSP126 | Malaria | -3.88 | (-32.59, 24.82) | -4.59 | (-32.82, 23.64) | -3.33 | (-33.47, 26.8) | 6.8 | (-28.79, 42.38) |
| 2021-2040 | SSP245 | Malaria | -6.89 | (-35.74, 21.95) | -8.17 | (-36.35, 20.01) | -3.64 | (-34.93, 27.66) | 6.02 | (-29.68, 41.73) |
| 2021-2040 | SSP370 | Malaria | -4.38 | (-33.35, 24.59) | -5.18 | (-33.69, 23.32) | -2.53 | (-32.73, 27.66) | 8.19 | (-28.26, 44.63) |
| 2021-2040 | SSP585 | Malaria | -4.92 | (-33.78, 23.95) | -5.96 | (-34.29, 22.37) | -1.91 | (-33.12, 29.3) | 7.15 | (-28.61, 42.9) |
| 2041-2060 | SSP126 | Malaria | -3.23 | (-32.52, 26.06) | -4.52 | (-33.08, 24.04) | 0.85 | (-30.56, 32.26) | 6.07 | (-29.39, 41.53) |
| 2041-2060 | SSP245 | Malaria | -4.82 | (-35.24, 25.59) | -6.22 | (-36.1, 23.67) | -1.16 | (-32.98, 30.66) | 4.42 | (-30, 38.84) |
| 2041-2060 | SSP370 | Malaria | -5.47 | (-34.85, 23.91) | -6.81 | (-35.54, 21.92) | 2.4 | (-30.6, 35.4) | 8.17 | (-28.7, 45.03) |
| 2041-2060 | SSP585 | Malaria | -7.4 | (-36.67, 21.87) | -8.97 | (-37.53, 19.6) | 1.89 | (-32.22, 36) | 6.14 | (-29.48, 41.75) |
| 2061-2080 | SSP126 | Malaria | -1.09 | (-31.16, 28.98) | -1.35 | (-31.17, 28.47) | -1.59 | (-32.37, 29.18) | 4.13 | (-31.58, 39.84) |
| 2061-2080 | SSP245 | Malaria | -2.24 | (-33.25, 28.76) | -2.45 | (-33.32, 28.41) | -0.21 | (-33.53, 33.1) | 4.57 | (-31.31, 40.45) |
| 2061-2080 | SSP370 | Malaria | -3.51 | (-33.68, 26.66) | -3.75 | (-33.81, 26.31) | -2.69 | (-33.19, 27.81) | 5.96 | (-31.77, 43.69) |
| 2061-2080 | SSP585 | Malaria | -7.15 | (-36.72, 22.41) | -7.28 | (-36.8, 22.23) | -7.78 | (-38.25, 22.69) | 3.49 | (-32.8, 39.77) |
| 2081-2100 | SSP126 | Malaria | -4.73 | (-33.56, 24.1) | -5.77 | (-34.02, 22.47) | 0.4 | (-31.09, 31.89) | 5.35 | (-29.78, 40.48) |
| 2081-2100 | SSP245 | Malaria | -7.53 | (-36.5, 21.45) | -8.54 | (-37.07, 19.99) | -1.93 | (-35.03, 31.17) | 3.47 | (-30.24, 37.17) |
| 2081-2100 | SSP370 | Malaria | -10.94 | (-40, 18.12) | -11.86 | (-40.6, 16.88) | -5.24 | (-37.59, 27.12) | 6.72 | (-30.55, 43.98) |
| 2081-2100 | SSP585 | Malaria | -12.06 | (-41.37, 17.24) | -12.25 | (-41.51, 17.02) | -8.5 | (-39.1, 22.1) | -1.98 | (-36.27, 32.32) |
| 2021-2040 | SSP126 | Melioidosis | -1.22 | (-8.12, 5.68) | -1.4 | (-8.25, 5.45) | -1.2 | (-8.65, 6.24) | 2.05 | (-5.88, 9.98) |
| 2021-2040 | SSP245 | Melioidosis | -1.08 | (-8.27, 6.11) | -1.2 | (-8.35, 5.95) | -0.33 | (-8.07, 7.42) | 1.3 | (-6.62, 9.23) |
| 2021-2040 | SSP370 | Melioidosis | -0.85 | (-7.95, 6.25) | -1 | (-8.05, 6.06) | 0.05 | (-7.51, 7.61) | 1.84 | (-6.09, 9.77) |
| 2021-2040 | SSP585 | Melioidosis | -0.88 | (-7.97, 6.2) | -1 | (-8.05, 6.05) | -2.13 | (-9.52, 5.27) | 2.67 | (-5.18, 10.52) |
| 2041-2060 | SSP126 | Melioidosis | 0.46 | (-6.65, 7.56) | 0.34 | (-6.73, 7.4) | -0.34 | (-8.05, 7.36) | 2.52 | (-5.3, 10.35) |
| 2041-2060 | SSP245 | Melioidosis | 0.64 | (-6.91, 8.18) | 0.6 | (-6.93, 8.13) | -1.4 | (-9.16, 6.36) | 2.77 | (-5.14, 10.68) |
| 2041-2060 | SSP370 | Melioidosis | 1.05 | (-6.47, 8.58) | 0.98 | (-6.53, 8.48) | 1.36 | (-6.58, 9.29) | 2.67 | (-5.32, 10.66) |
| 2041-2060 | SSP585 | Melioidosis | 2.02 | (-5.85, 9.89) | 1.99 | (-5.87, 9.86) | 1.21 | (-6.72, 9.14) | 2.18 | (-5.76, 10.12) |
| 2061-2080 | SSP126 | Melioidosis | 0.39 | (-6.74, 7.52) | 0.4 | (-6.7, 7.51) | -0.62 | (-8.21, 6.97) | -0.25 | (-8.18, 7.68) |
| 2061-2080 | SSP245 | Melioidosis | 0.74 | (-6.75, 8.23) | 0.77 | (-6.71, 8.24) | -1.59 | (-9.67, 6.48) | -1.33 | (-9.32, 6.66) |
| 2061-2080 | SSP370 | Melioidosis | 1.02 | (-6.58, 8.62) | 1.1 | (-6.5, 8.7) | -2.06 | (-9.68, 5.56) | -1.85 | (-9.99, 6.28) |
| 2061-2080 | SSP585 | Melioidosis | 2.29 | (-5.98, 10.55) | 2.37 | (-5.9, 10.65) | -2.57 | (-10.45, 5.31) | 0.84 | (-7.72, 9.39) |
| 2081-2100 | SSP126 | Melioidosis | -0.74 | (-7.79, 6.32) | -0.82 | (-7.84, 6.2) | -2.22 | (-9.6, 5.17) | 2.36 | (-5.49, 10.21) |
| 2081-2100 | SSP245 | Melioidosis | -0.08 | (-7.51, 7.35) | -0.09 | (-7.5, 7.32) | -2.48 | (-10.33, 5.38) | 1.95 | (-5.8, 9.7) |
| 2081-2100 | SSP370 | Melioidosis | 1.2 | (-7.32, 9.73) | 1.33 | (-7.23, 9.89) | -2.61 | (-10.61, 5.39) | 0.81 | (-7.26, 8.89) |
| 2081-2100 | SSP585 | Melioidosis | 1.92 | (-7, 10.85) | 2 | (-6.94, 10.94) | -2.63 | (-10.72, 5.45) | 2.05 | (-7.23, 11.34) |
| 2021-2040 | SSP126 | Pneumonia | 2.33 | (-32.59, 37.25) | -0.2 | (-33.46, 33.07) | 24.91 | (-18.68, 68.49) | 9.93 | (-32.64, 52.49) |
| 2021-2040 | SSP245 | Pneumonia | -1.38 | (-35.95, 33.19) | -5.29 | (-37.64, 27.06) | 24.04 | (-21, 69.08) | 11.69 | (-32.24, 55.63) |
| 2021-2040 | SSP370 | Pneumonia | 3.51 | (-32.1, 39.13) | 0.5 | (-33.24, 34.23) | 25.23 | (-18.49, 68.94) | 14.57 | (-30.3, 59.44) |
| 2021-2040 | SSP585 | Pneumonia | 2.47 | (-32.7, 37.65) | -0.46 | (-33.81, 32.89) | 22.79 | (-19.44, 65.02) | 10.96 | (-32.12, 54.03) |
| 2041-2060 | SSP126 | Pneumonia | 2.77 | (-32.6, 38.14) | 0.53 | (-33.02, 34.08) | 20.65 | (-20.62, 61.92) | 11.03 | (-32.9, 54.95) |
| 2041-2060 | SSP245 | Pneumonia | 2.01 | (-34.35, 38.36) | -1.39 | (-35.8, 33.01) | 20.34 | (-22.57, 63.26) | 12.14 | (-32.01, 56.29) |
| 2041-2060 | SSP370 | Pneumonia | 2.32 | (-33.11, 37.75) | -0.32 | (-33.96, 33.33) | 24.53 | (-18.82, 67.89) | 14.94 | (-30.6, 60.48) |
| 2041-2060 | SSP585 | Pneumonia | 1.37 | (-33.79, 36.53) | -1.05 | (-34.75, 32.66) | 24.2 | (-17.86, 66.27) | 9.19 | (-32.86, 51.23) |
| 2061-2080 | SSP126 | Pneumonia | 6.33 | (-29.84, 42.51) | 5.5 | (-29.94, 40.93) | 24.26 | (-18.15, 66.67) | 9.64 | (-33.46, 52.75) |
| 2061-2080 | SSP245 | Pneumonia | 5.06 | (-31.66, 41.78) | 4.52 | (-31.77, 40.8) | 30.58 | (-16.05, 77.21) | 10.25 | (-33.18, 53.69) |
| 2061-2080 | SSP370 | Pneumonia | 9.48 | (-28.38, 47.33) | 8.33 | (-28.88, 45.54) | 30.05 | (-15.28, 75.37) | 15.98 | (-30.92, 62.88) |
| 2061-2080 | SSP585 | Pneumonia | 5.39 | (-30.88, 41.66) | 4.91 | (-31.11, 40.93) | 26.75 | (-16.49, 70) | 10.64 | (-31.89, 53.17) |
| 2081-2100 | SSP126 | Pneumonia | 2.22 | (-32.8, 37.25) | 0.35 | (-33.22, 33.92) | 26.23 | (-17.29, 69.74) | 10.34 | (-32.67, 53.36) |
| 2081-2100 | SSP245 | Pneumonia | -3.79 | (-36.9, 29.31) | -5.69 | (-37.86, 26.47) | 24.5 | (-19, 68) | 4.19 | (-34.52, 42.9) |
| 2081-2100 | SSP370 | Pneumonia | 1.9 | (-33.63, 37.42) | 0.14 | (-34.58, 34.86) | 30.81 | (-15.04, 76.67) | 17.42 | (-29.41, 64.26) |
| 2081-2100 | SSP585 | Pneumonia | 1.14 | (-34.28, 36.56) | 0.6 | (-34.6, 35.8) | 26.31 | (-16.83, 69.46) | 10.31 | (-32.08, 52.7) |
