# Supplementary figures and images for: Projecting long-term excess risks of major infectious diseases associated with future extreme weather events in Thailand
Source: PLoS Negl Trop Dis. 2026 Jan 5;20(1):e0013896. doi: 10.1371/journal.pntd.0013896 (PMC12782439; doi:10.1371/journal.pntd.0013896)

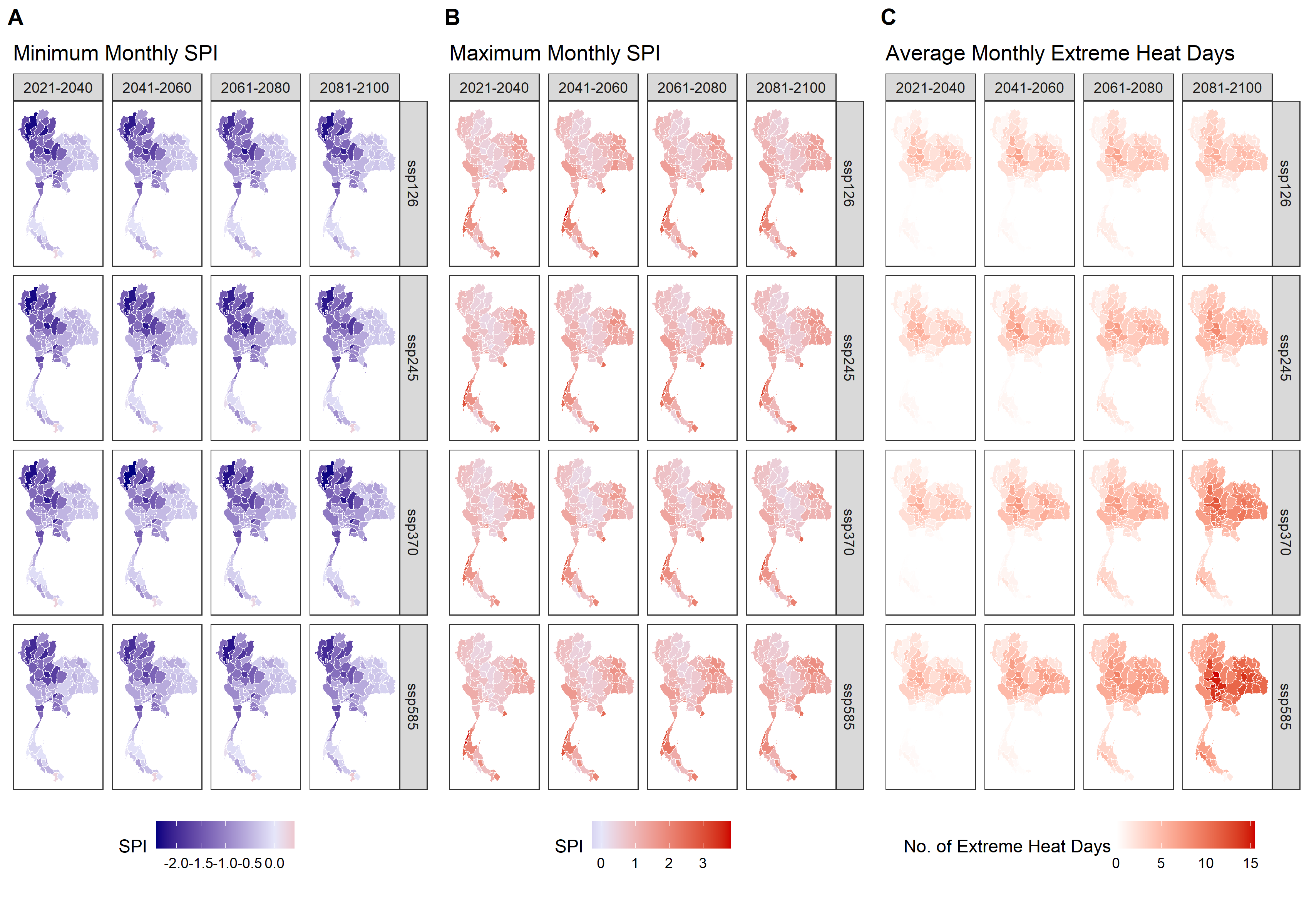

Supplement: S1 Fig — Monthly maximum, minimum temperature and total precipitation from the MIROC6 general circulation model was interpolated to daily frequency. Total precipitation was converted to Standardised Precipitation Index (SPI) and the number of extreme heat days were counted from the daily maximum temperature. An extreme heat day is one where the maximum temperature exceeds the 90th percentile of historical national maximum temperature, for a period of three or more days. Map created using GADM data (https://gadm.org/index.html, freely available for academic use). The map outlines and administrative boundaries are used with permission for academic publishing. (PNG) [file pntd.0013896.s007.png]

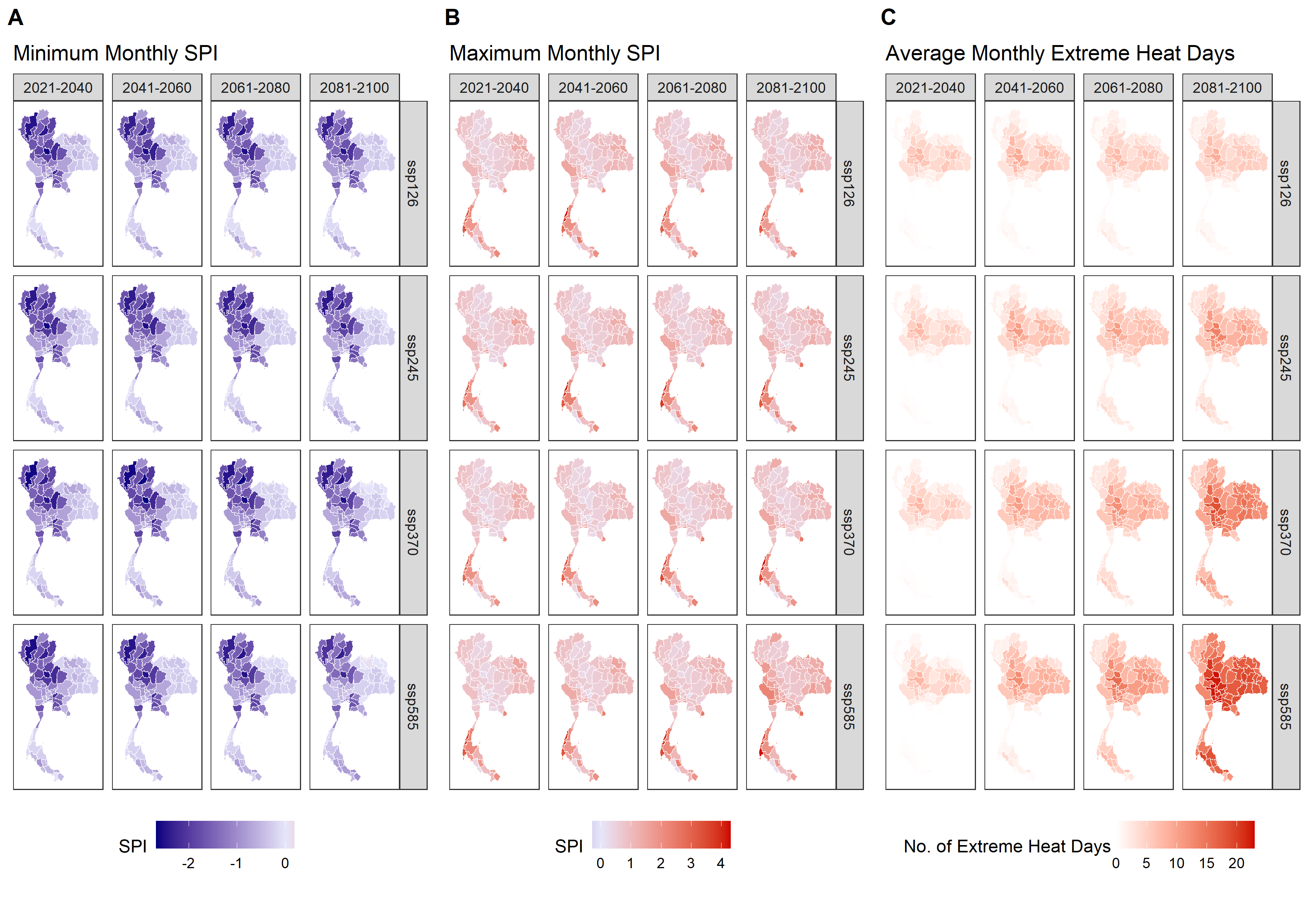

Supplement: S2 Fig — Monthly maximum, minimum temperature and total precipitation from the IPSL-CM6A-LR general circulation model was interpolated to daily frequency. Total precipitation was converted to Standardised Precipitation Index (SPI) and the number of extreme heat days were counted from the daily maximum temperature. An extreme heat day is one where the maximum temperature exceeds the 90th percentile of historical national maximum temperature, for a period of three or more days. Map created using GADM data (https://gadm.org/index.html, freely available for academic use). The map outlines and administrative boundaries are used with permission for academic publishing. (PNG) [file pntd.0013896.s008.png]

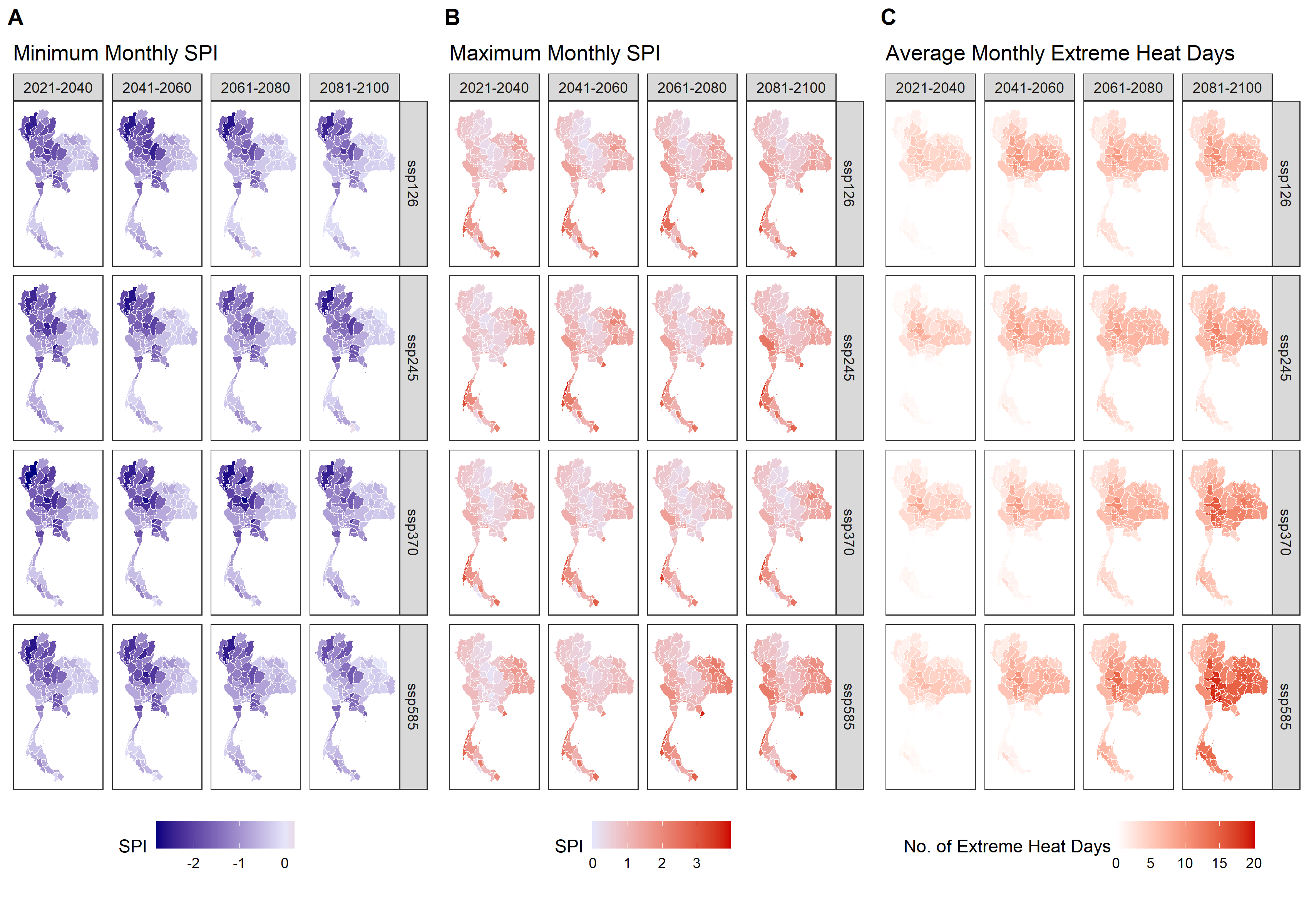

Supplement: S3 Fig — Monthly maximum, minimum temperature and total precipitation from the CMCC-ESM2 general circulation model was interpolated to daily frequency. Total precipitation was converted to Standardised Precipitation Index (SPI) and the number of extreme heat days were counted from the daily maximum temperature. An extreme heat day is one where the maximum temperature exceeds the 90th percentile of historical national maximum temperature, for a period of three or more days. Map created using GADM data (https://gadm.org/index.html, freely available for academic use). The map outlines and administrative boundaries are used with permission for academic publishing. (PNG) [file pntd.0013896.s009.png]

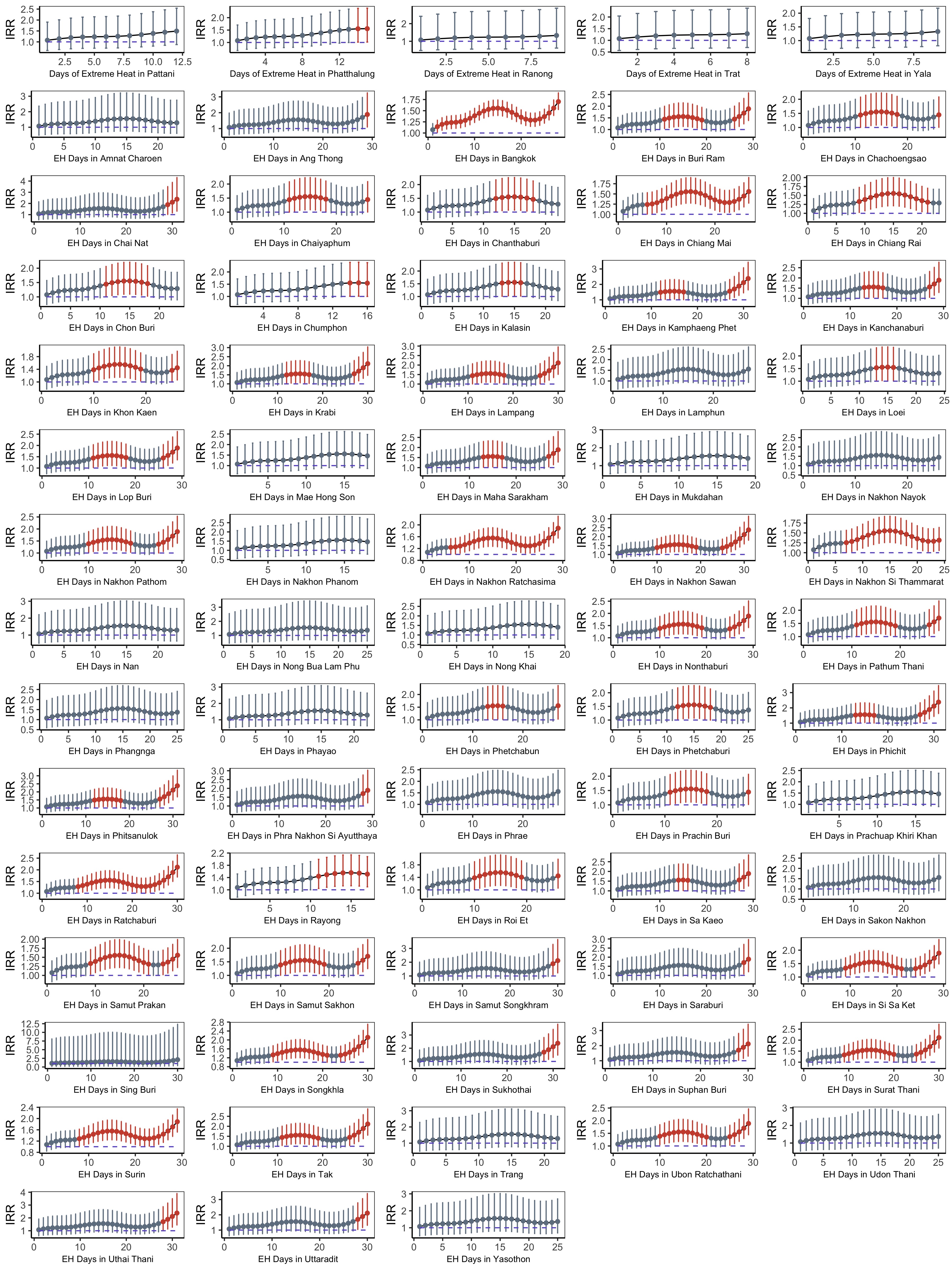

Supplement: S4 Fig — Fig show incidence rate ratio (IRR) of each disease over days of extreme heat for each province. The IRR gives the ratio of predicted cases with exposure to predicted cases with no exposure obtained from the disease-specific generalised additive models. The IRR for extreme heat gives the ratio between incidence rates at non-zero days of extreme heat in a month and the incidence rate with no days of extreme heat. An IRR above 1 represents increased incidence rate with exposure compared to no exposure, while an IRR below 1 represents decreased incidence rate. Points represent point estimates for the IRR with accompanying 95% confidence intervals. IRRs were derived using the ratio of predicted cases with exposure and predicted cases without exposure. Statistical significance was denoted with orange points when 95% CIs of the IRRs do not cross 1. (JPEG) [file pntd.0013896.s010.jpeg]

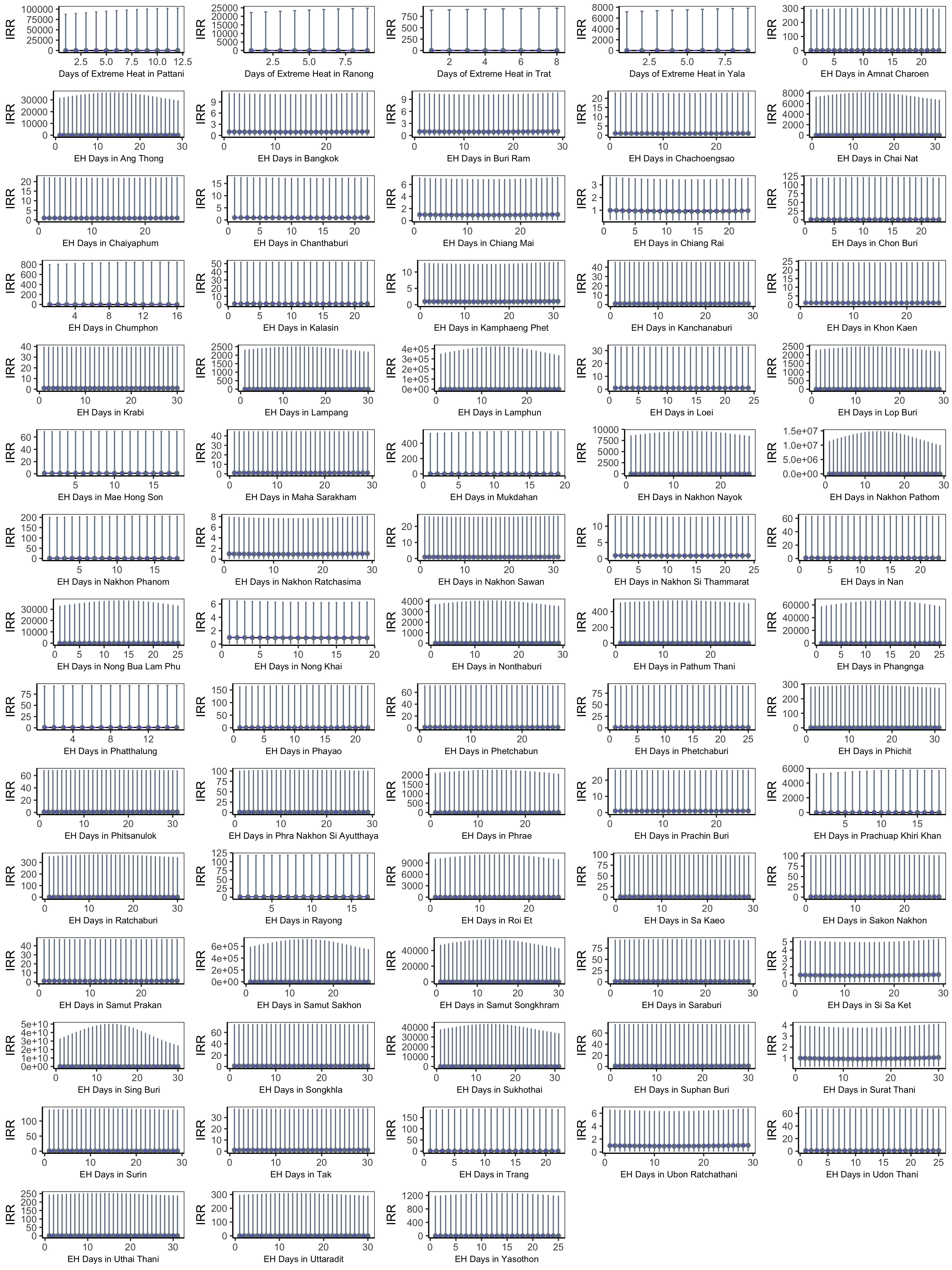

Supplement: S5 Fig — Fig show incidence rate ratio (IRR) of each disease over days of extreme heat for each province. The IRR gives the ratio of predicted cases with exposure to predicted cases with no exposure obtained from the disease-specific generalised additive models. The IRR for extreme heat gives the ratio between incidence rates at non-zero days of extreme heat in a month and the incidence rate with no days of extreme heat. An IRR above 1 represents increased incidence rate with exposure compared to no exposure, while an IRR below 1 represents decreased incidence rate. Points represent point estimates for the IRR with accompanying 95% confidence intervals. IRRs were derived using the ratio of predicted cases with exposure and predicted cases without exposure. Statistical significance was denoted with orange points when 95% CIs of the IRRs do not cross 1. (JPEG) [file pntd.0013896.s011.jpeg]

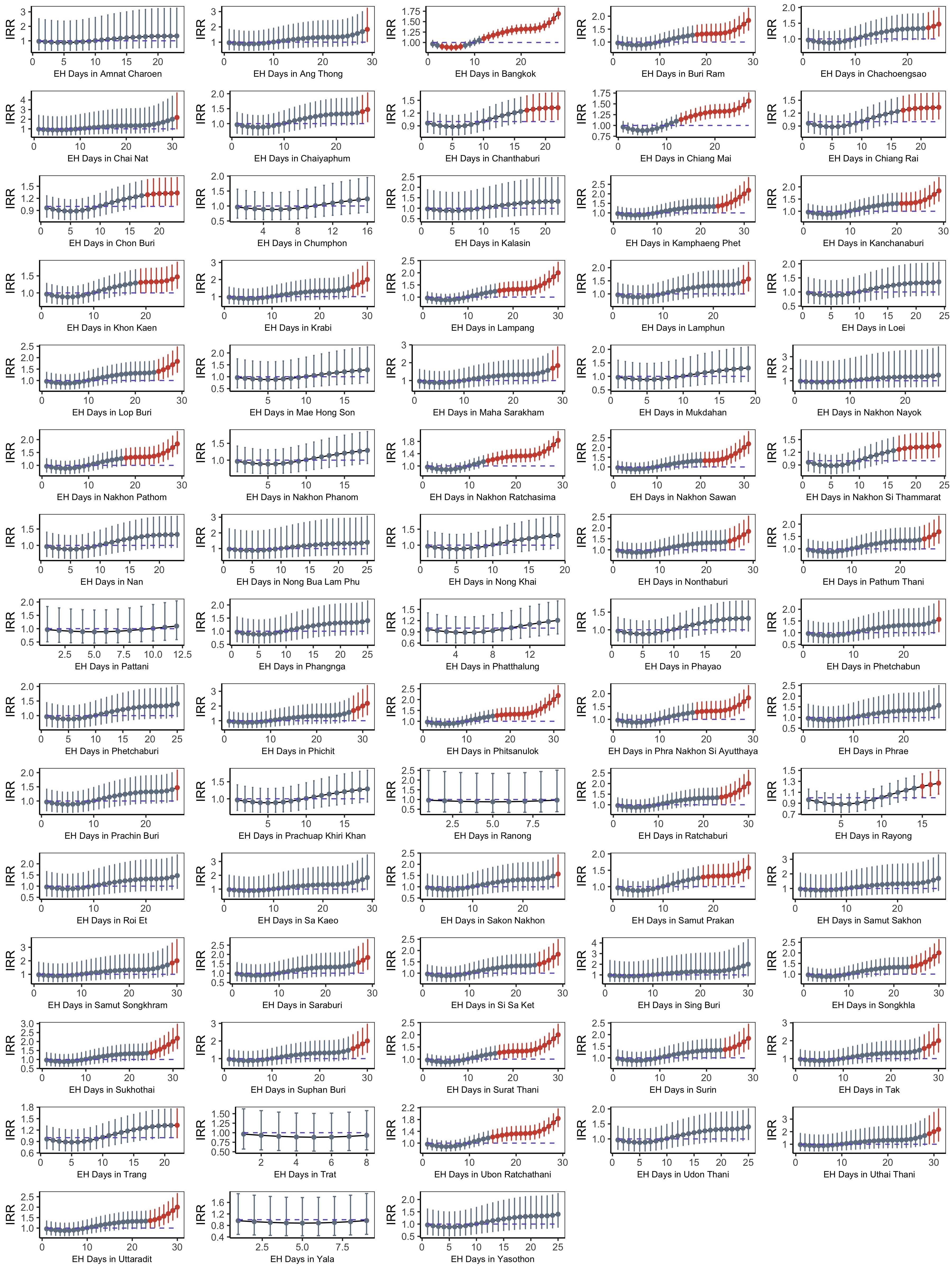

Supplement: S6 Fig — Fig show incidence rate ratio (IRR) of each disease over days of extreme heat for each province. The IRR gives the ratio of predicted cases with exposure to predicted cases with no exposure obtained from the disease-specific generalised additive models. The IRR for extreme heat gives the ratio between incidence rates at non-zero days of extreme heat in a month and the incidence rate with no days of extreme heat. An IRR above 1 represents increased incidence rate with exposure compared to no exposure, while an IRR below 1 represents decreased incidence rate. Points represent point estimates for the IRR with accompanying 95% confidence intervals. IRRs were derived using the ratio of predicted cases with exposure and predicted cases without exposure. Statistical significance was denoted with orange points when 95% CIs of the IRRs do not cross 1. (JPEG) [file pntd.0013896.s012.jpeg]

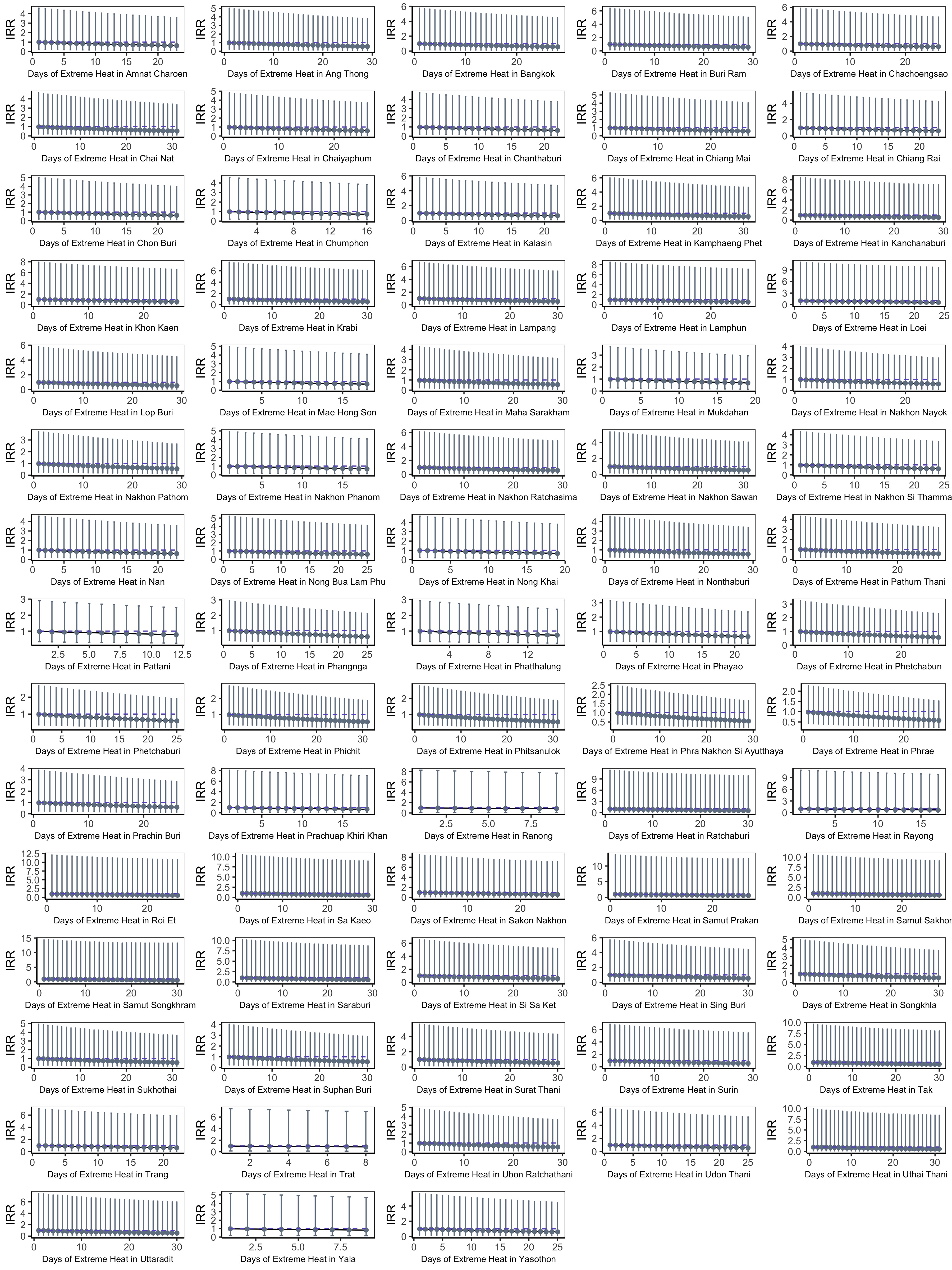

Supplement: S7 Fig — Fig show incidence rate ratio (IRR) of each disease over days of extreme heat for each province. The IRR gives the ratio of predicted cases with exposure to predicted cases with no exposure obtained from the disease-specific generalised additive models. The IRR for extreme heat gives the ratio between incidence rates at non-zero days of extreme heat in a month and the incidence rate with no days of extreme heat. An IRR above 1 represents increased incidence rate with exposure compared to no exposure, while an IRR below 1 represents decreased incidence rate. Points represent point estimates for the IRR with accompanying 95% confidence intervals. IRRs were derived using the ratio of predicted cases with exposure and predicted cases without exposure. Statistical significance was denoted with orange points when 95% CIs of the IRRs do not cross 1. (JPEG) [file pntd.0013896.s013.jpeg]

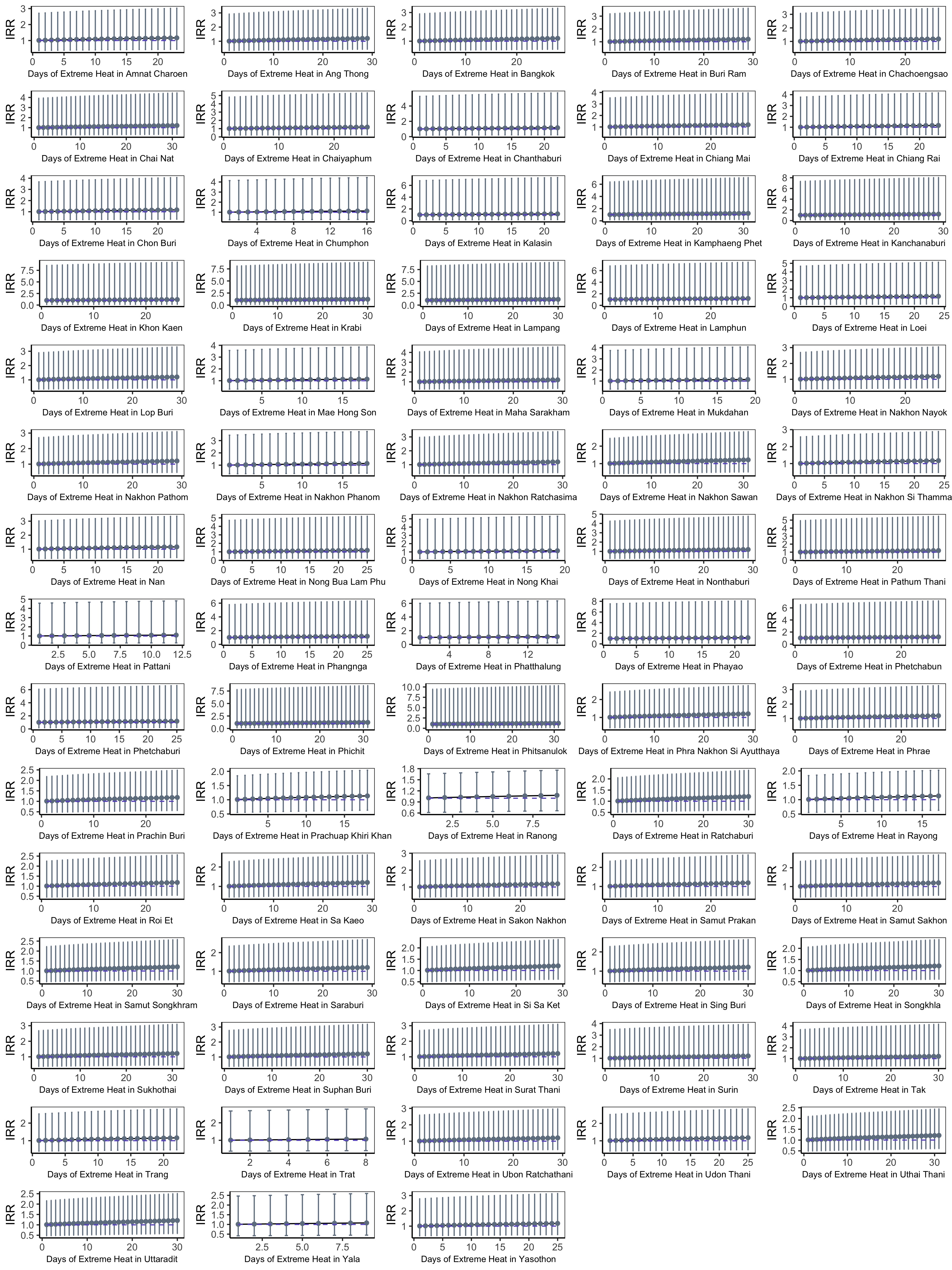

Supplement: S8 Fig — Fig show incidence rate ratio (IRR) of each disease over days of extreme heat for each province. The IRR gives the ratio of predicted cases with exposure to predicted cases with no exposure obtained from the disease-specific generalised additive models. The IRR for extreme heat gives the ratio between incidence rates at non-zero days of extreme heat in a month and the incidence rate with no days of extreme heat. An IRR above 1 represents increased incidence rate with exposure compared to no exposure, while an IRR below 1 represents decreased incidence rate. Points represent point estimates for the IRR with accompanying 95% confidence intervals. IRRs were derived using the ratio of predicted cases with exposure and predicted cases without exposure. Statistical significance was denoted with orange points when 95% CIs of the IRRs do not cross 1. (JPEG) [file pntd.0013896.s014.jpeg]

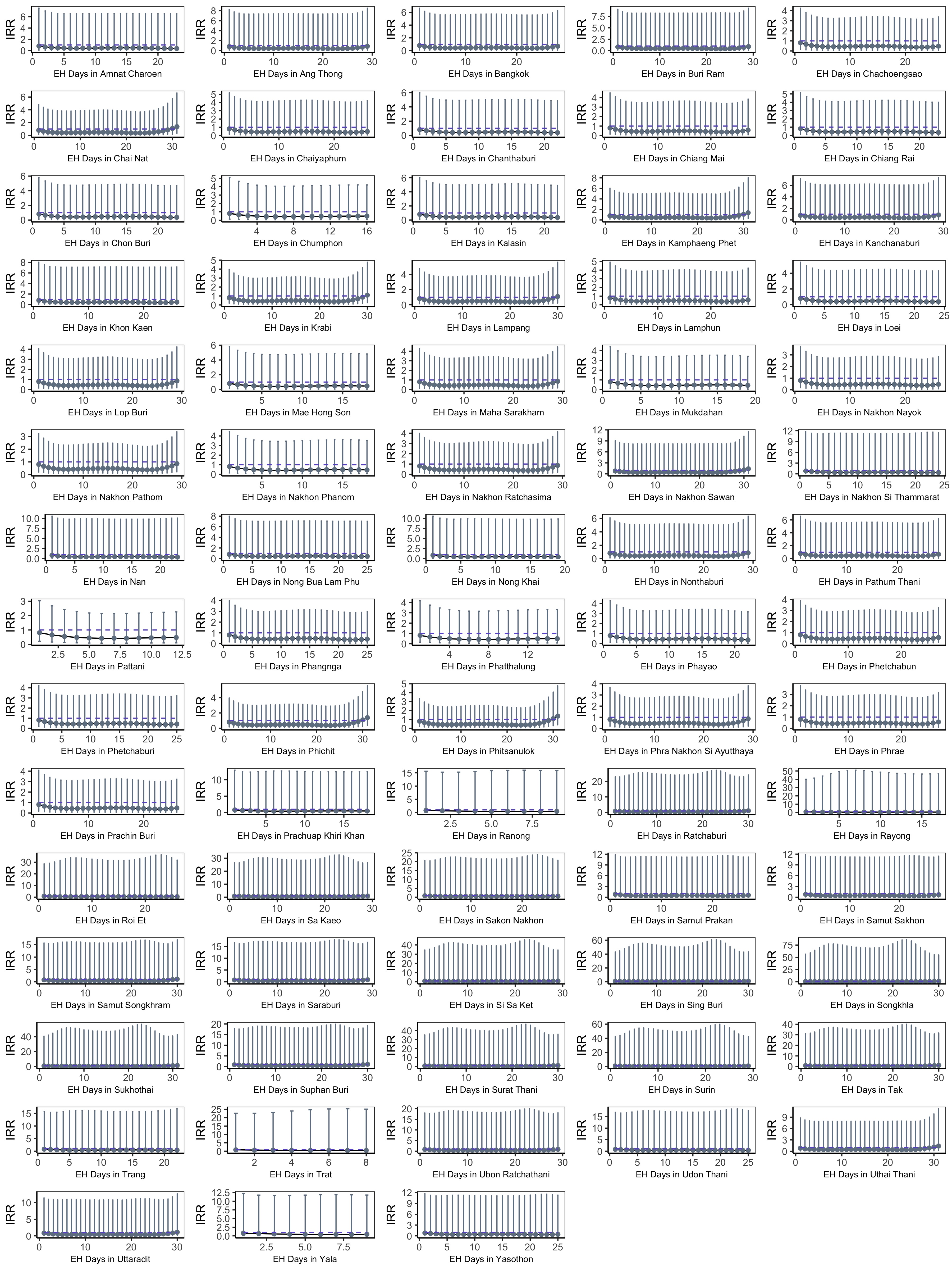

Supplement: S9 Fig — Fig show incidence rate ratio (IRR) of each disease over days of extreme heat for each province. The IRR gives the ratio of predicted cases with exposure to predicted cases with no exposure obtained from the disease-specific generalised additive models. The IRR for extreme heat gives the ratio between incidence rates at non-zero days of extreme heat in a month and the incidence rate with no days of extreme heat. An IRR above 1 represents increased incidence rate with exposure compared to no exposure, while an IRR below 1 represents decreased incidence rate. Points represent point estimates for the IRR with accompanying 95% confidence intervals. IRRs were derived using the ratio of predicted cases with exposure and predicted cases without exposure. Statistical significance was denoted with orange points when 95% CIs of the IRRs do not cross 1. (JPEG) [file pntd.0013896.s015.jpeg]

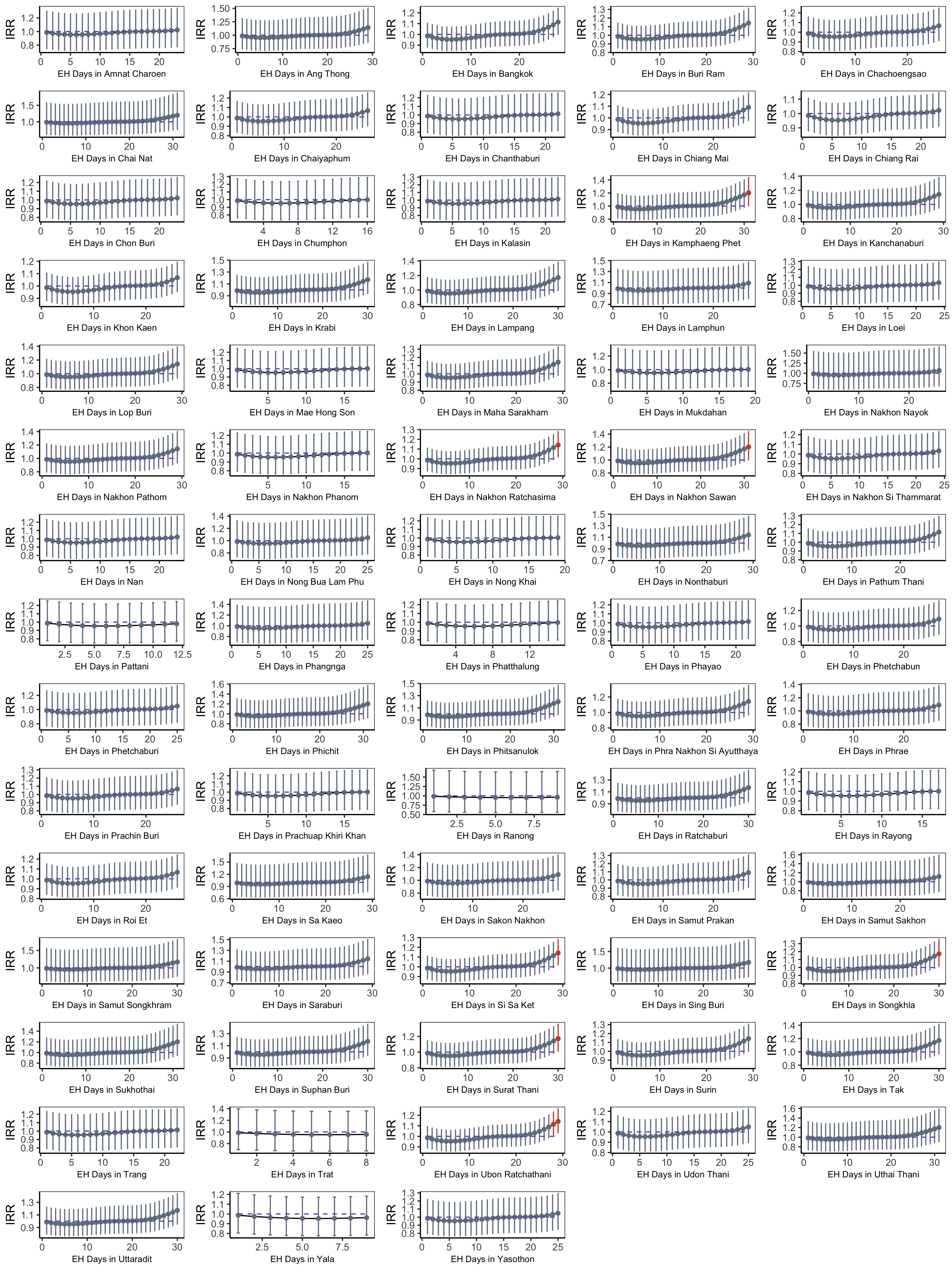

Supplement: S10 Fig — Fig show incidence rate ratio (IRR) of each disease over days of extreme heat for each province. The IRR gives the ratio of predicted cases with exposure to predicted cases with no exposure obtained from the disease-specific generalised additive models. The IRR for extreme heat gives the ratio between incidence rates at non-zero days of extreme heat in a month and the incidence rate with no days of extreme heat. An IRR above 1 represents increased incidence rate with exposure compared to no exposure, while an IRR below 1 represents decreased incidence rate. Points represent point estimates for the IRR with accompanying 95% confidence intervals. IRRs were derived using the ratio of predicted cases with exposure and predicted cases without exposure. Statistical significance was denoted with orange points when 95% CIs of the IRRs do not cross 1. (JPEG) [file pntd.0013896.s016.jpeg]

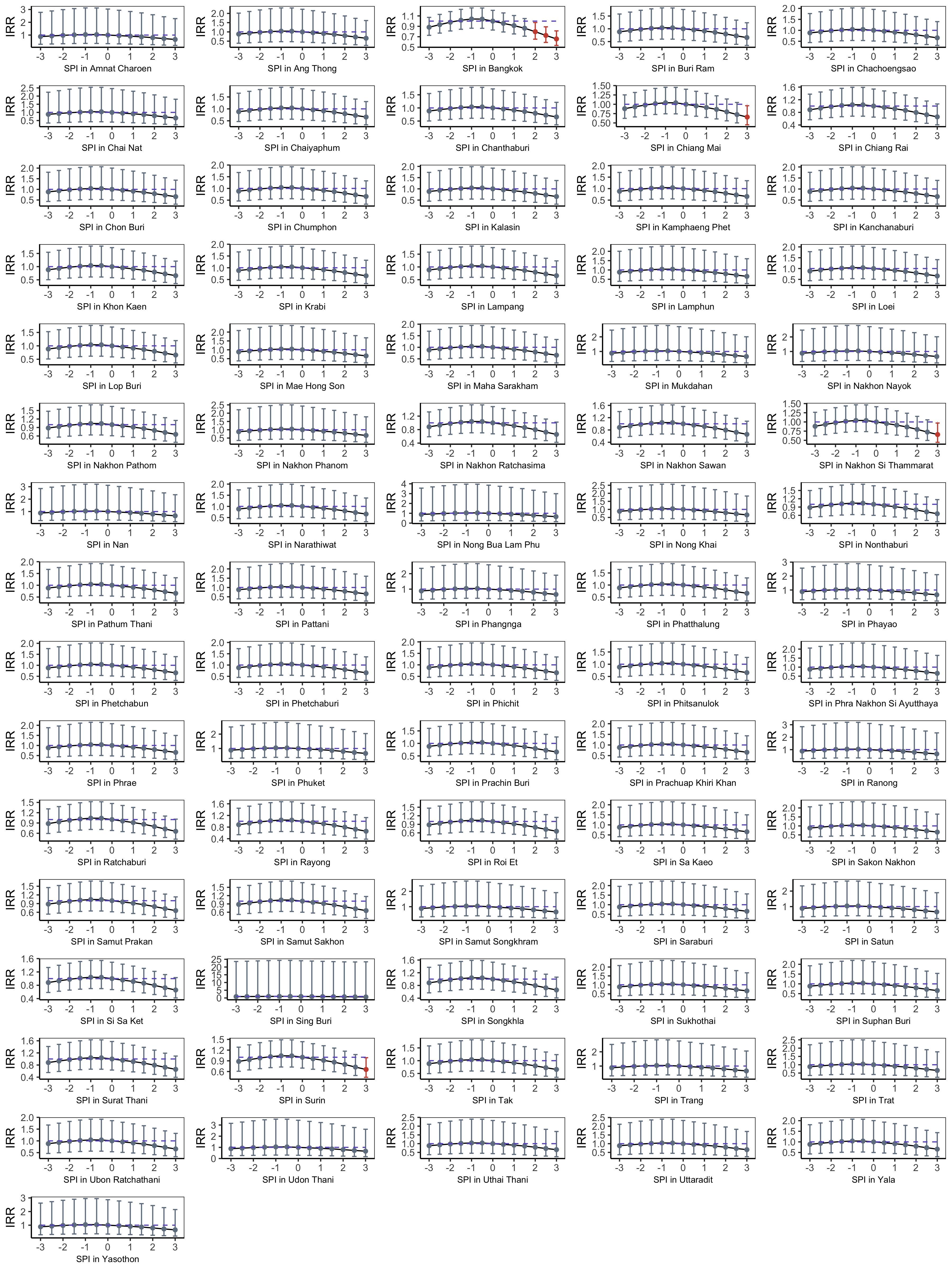

Supplement: S11 Fig — Fig shows incidence rate ratio (IRR) of each disease over days of SPI for each province. The IRR gives the ratio of predicted cases with exposure to predicted cases with no exposure obtained from the disease-specific generalised additive models. Specifically, the IRR for SPI gives the ratio between incidence rates at varying values of SPI and the incidence rate at 0 SPI. An SPI value of 0 represents average precipitation conditions based on the long-term mean precipitation in that location. An IRR above 1 represents increased incidence rate with exposure compared to no exposure, while an IRR below 1 represents decreased incidence rate. Points represent point estimates for the IRR with accompanying 95% confidence intervals. IRRs were derived using the ratio of predicted cases with exposure and predicted cases without exposure. Statistical significance was denoted with orange points when 95% CIs of the IRRs do not cross 1. (JPEG) [file pntd.0013896.s017.jpeg]

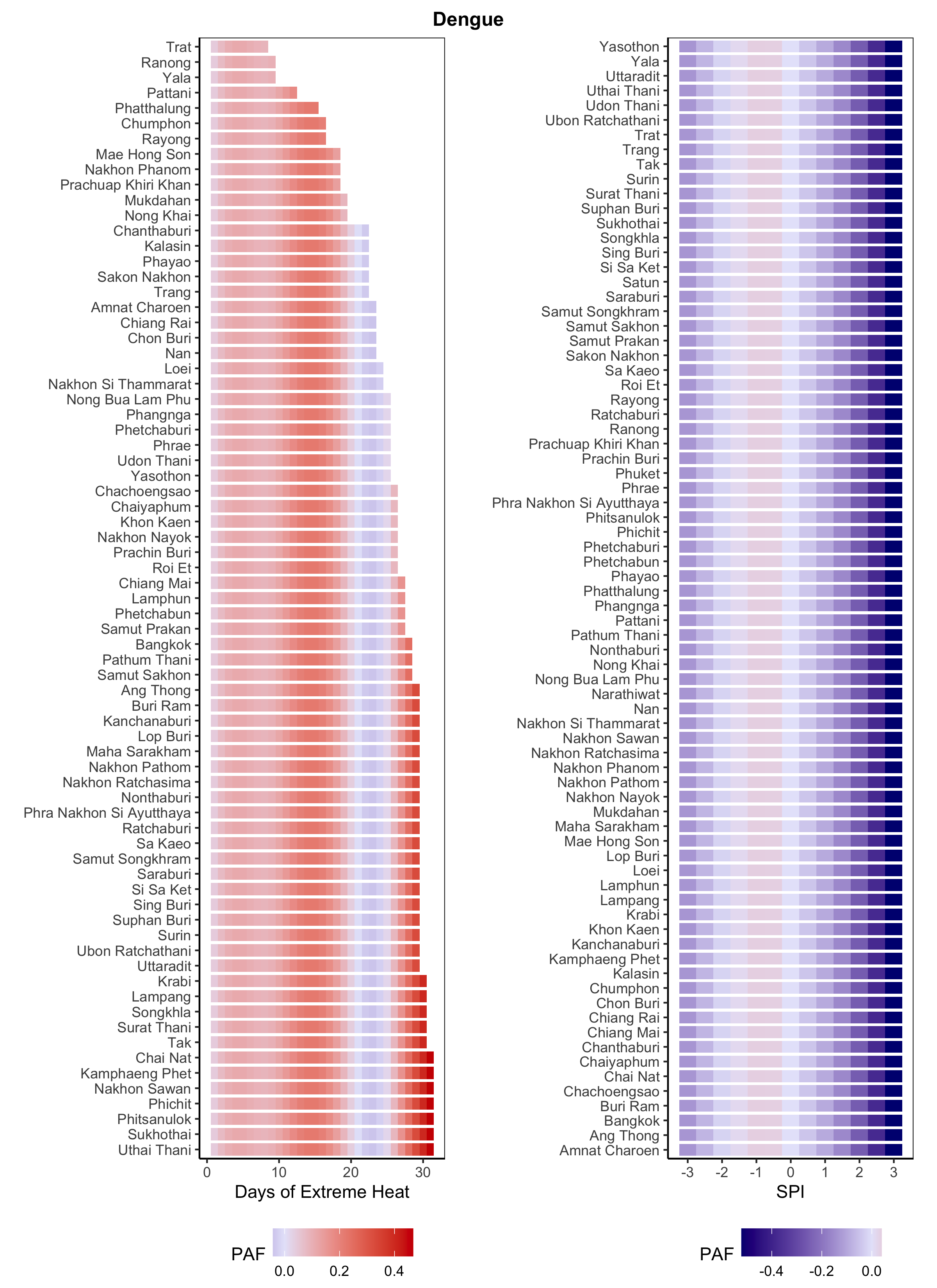

Supplement: S18 Fig — The population attributable fraction (PAF) gives the proportion of cases in a population that is attributed to a particular risk factor. A PAF of 0 indicates that the risk factor has no impact on the occurrence of the disease, and a negative PAF indicates that the factor has a protective effect against the disease. The province-specific PAF was estimated with the ratio of predicted cases when varying either SPI or extreme heat days from zero, with other covariates held constant, against predicted cases when SPI and extreme heat days are set to zero. Predicted cases were obtained from disease-specific generalised additive models. (PNG) [file pntd.0013896.s024.png]

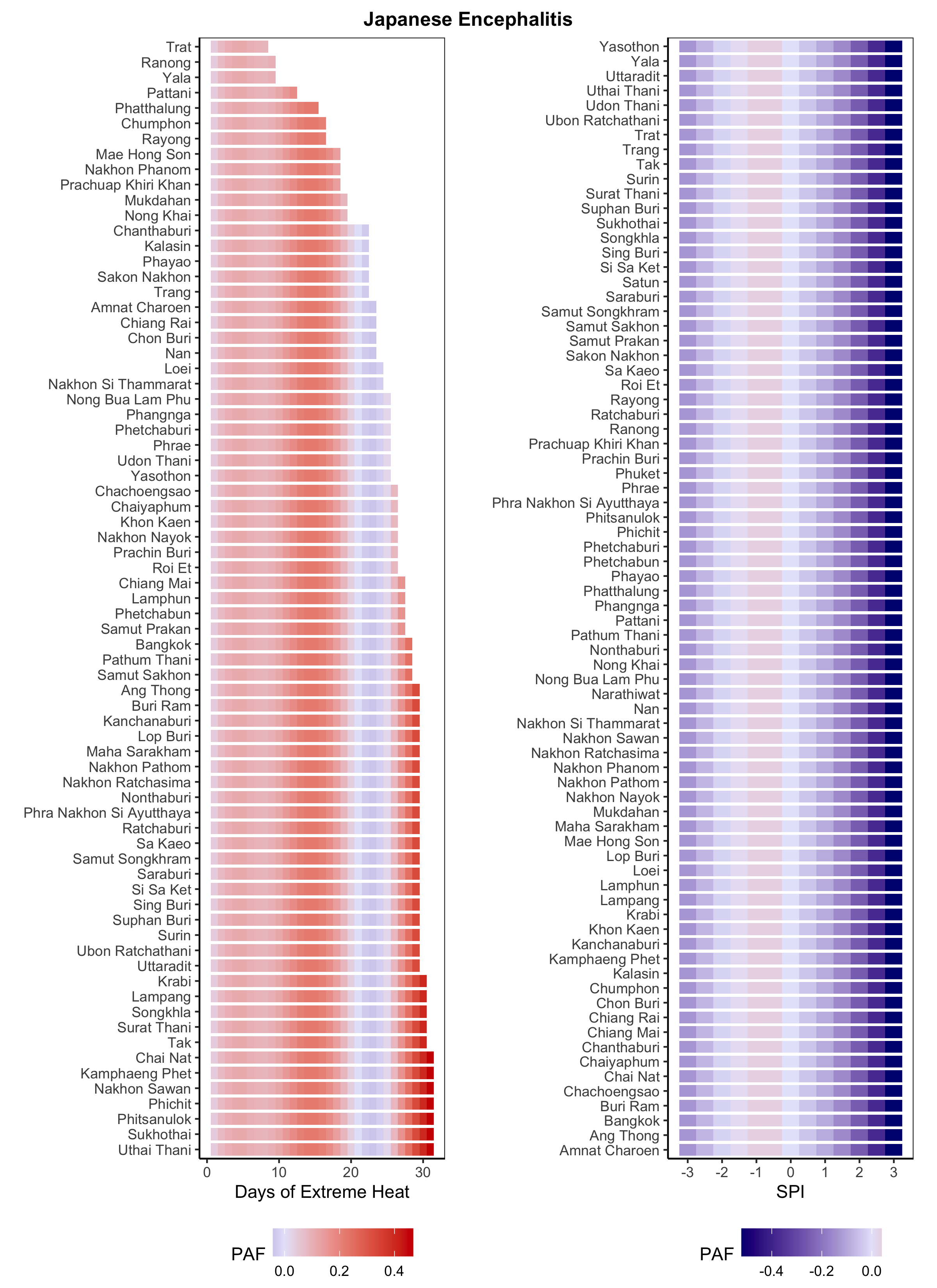

Supplement: S19 Fig — The population attributable fraction (PAF) gives the proportion of cases in a population that is attributed to a particular risk factor. A PAF of 0 indicates that the risk factor has no impact on the occurrence of the disease, and a negative PAF indicates that the factor has a protective effect against the disease. The province-specific PAF was estimated with the ratio of predicted cases when varying either SPI or extreme heat days from zero, with other covariates held constant, against predicted cases when SPI and extreme heat days are set to zero. Predicted cases were obtained from disease-specific generalised additive models. (PNG) [file pntd.0013896.s025.png]

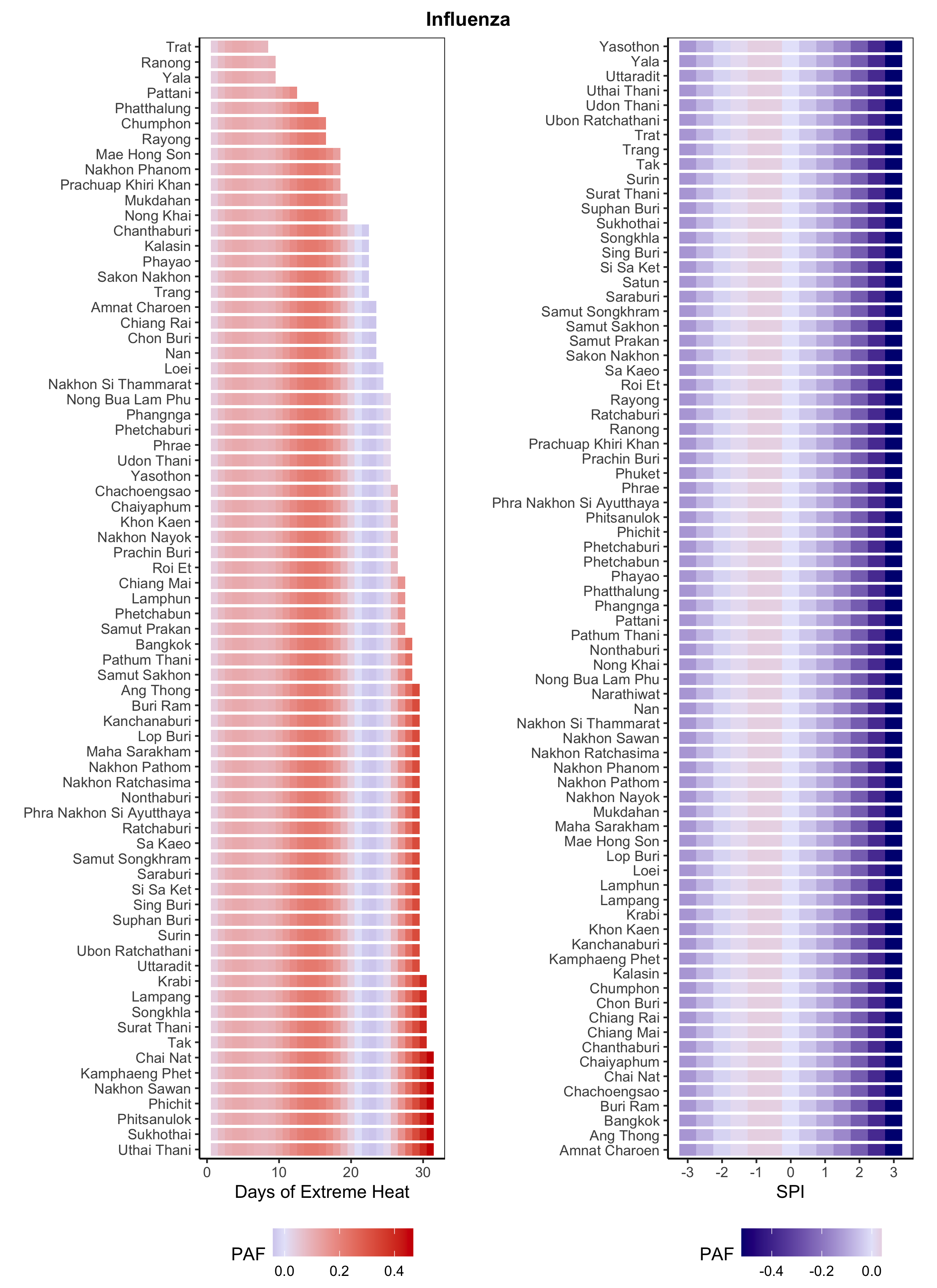

Supplement: S20 Fig — The population attributable fraction (PAF) gives the proportion of cases in a population that is attributed to a particular risk factor. A PAF of 0 indicates that the risk factor has no impact on the occurrence of the disease, and a negative PAF indicates that the factor has a protective effect against the disease. The province-specific PAF was estimated with the ratio of predicted cases when varying either SPI or extreme heat days from zero, with other covariates held constant, against predicted cases when SPI and extreme heat days are set to zero. Predicted cases were obtained from disease-specific generalised additive models. (PNG) [file pntd.0013896.s026.png]

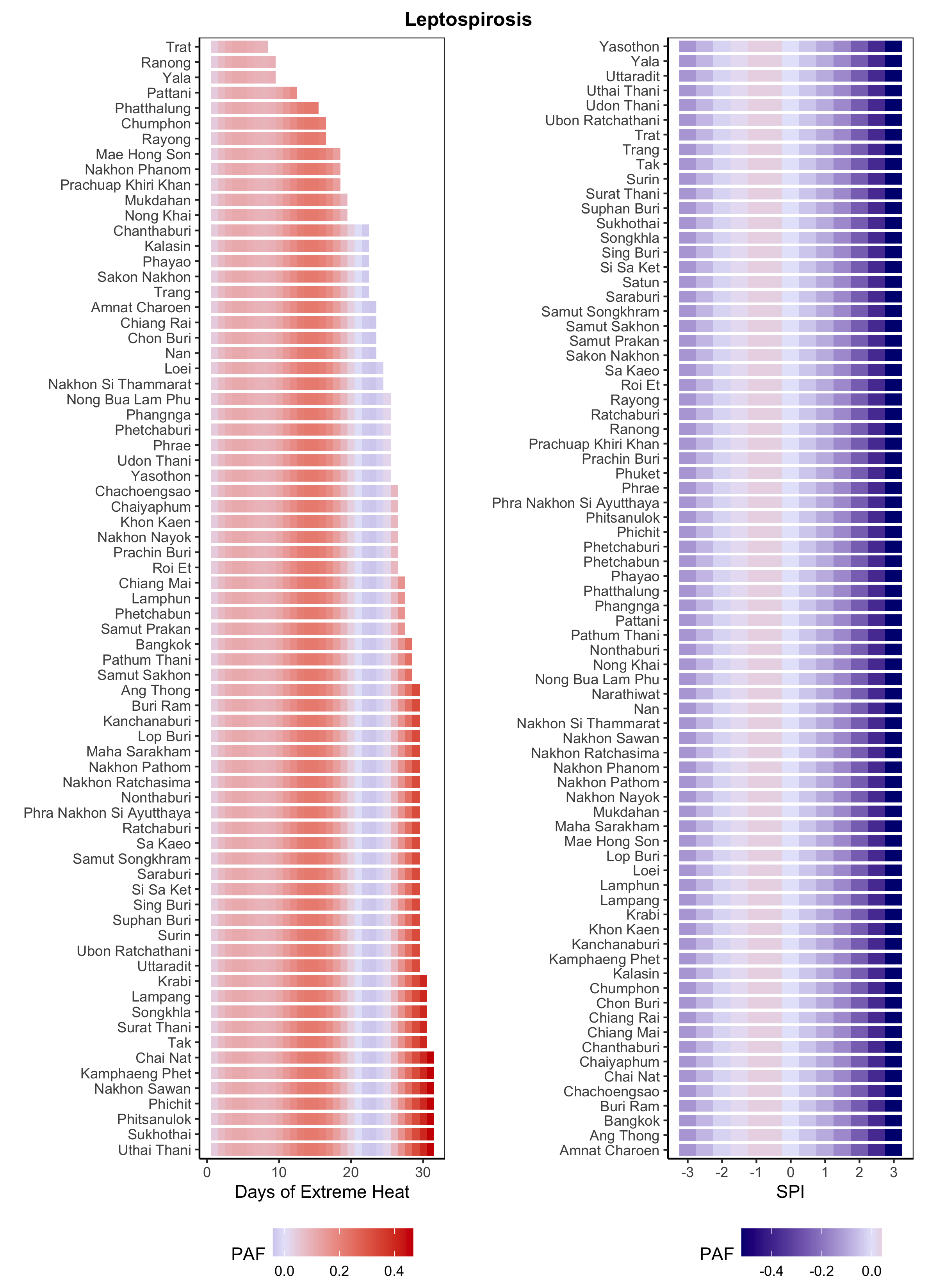

Supplement: S21 Fig — The population attributable fraction (PAF) gives the proportion of cases in a population that is attributed to a particular risk factor. A PAF of 0 indicates that the risk factor has no impact on the occurrence of the disease, and a negative PAF indicates that the factor has a protective effect against the disease. The province-specific PAF was estimated with the ratio of predicted cases when varying either SPI or extreme heat days from zero, with other covariates held constant, against predicted cases when SPI and extreme heat days are set to zero. Predicted cases were obtained from disease-specific generalised additive models. (PNG) [file pntd.0013896.s027.png]

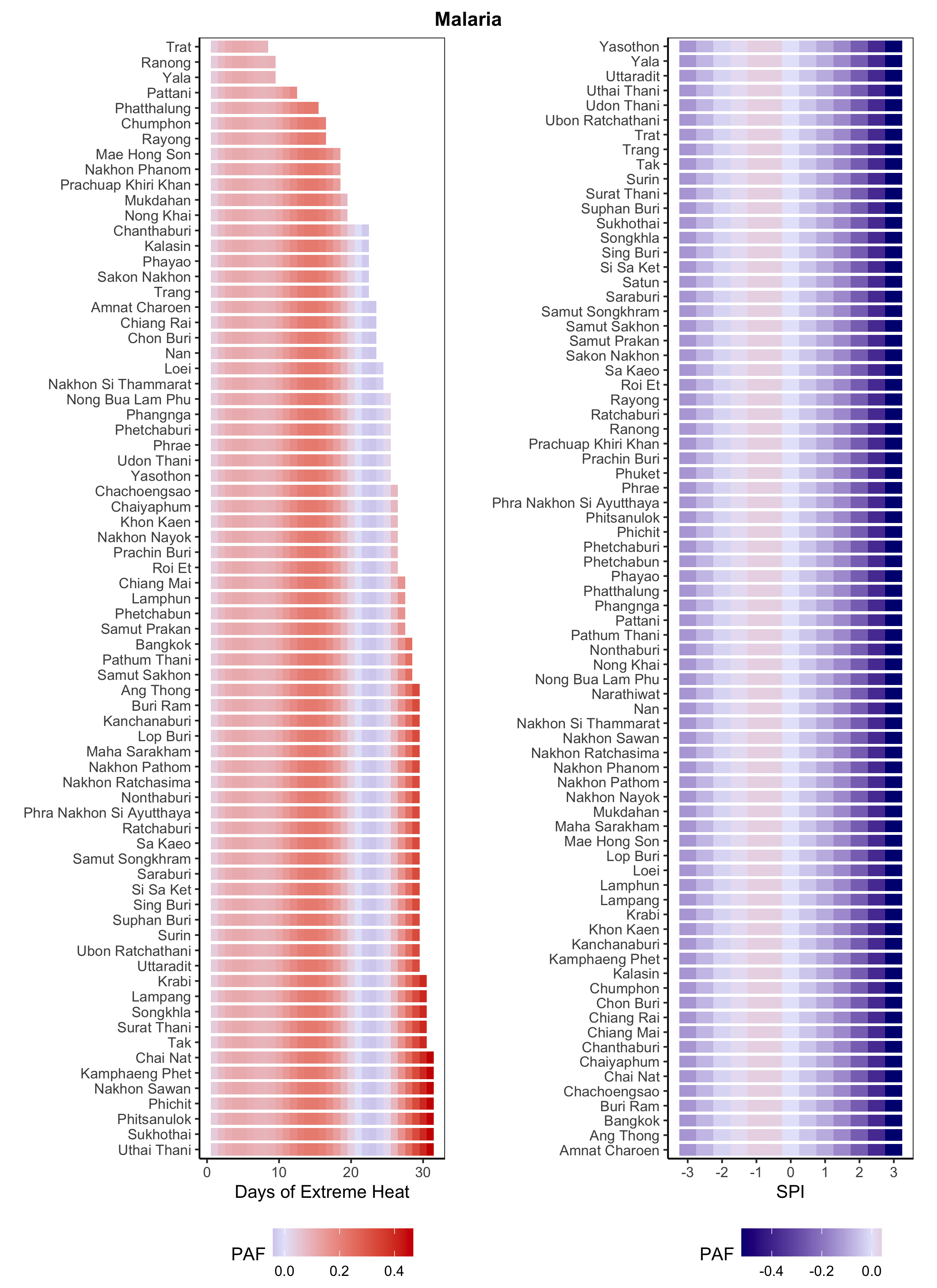

Supplement: S22 Fig — The population attributable fraction (PAF) gives the proportion of cases in a population that is attributed to a particular risk factor. A PAF of 0 indicates that the risk factor has no impact on the occurrence of the disease, and a negative PAF indicates that the factor has a protective effect against the disease. The province-specific PAF was estimated with the ratio of predicted cases when varying either SPI or extreme heat days from zero, with other covariates held constant, against predicted cases when SPI and extreme heat days are set to zero. Predicted cases were obtained from disease-specific generalised additive models. (PNG) [file pntd.0013896.s028.png]

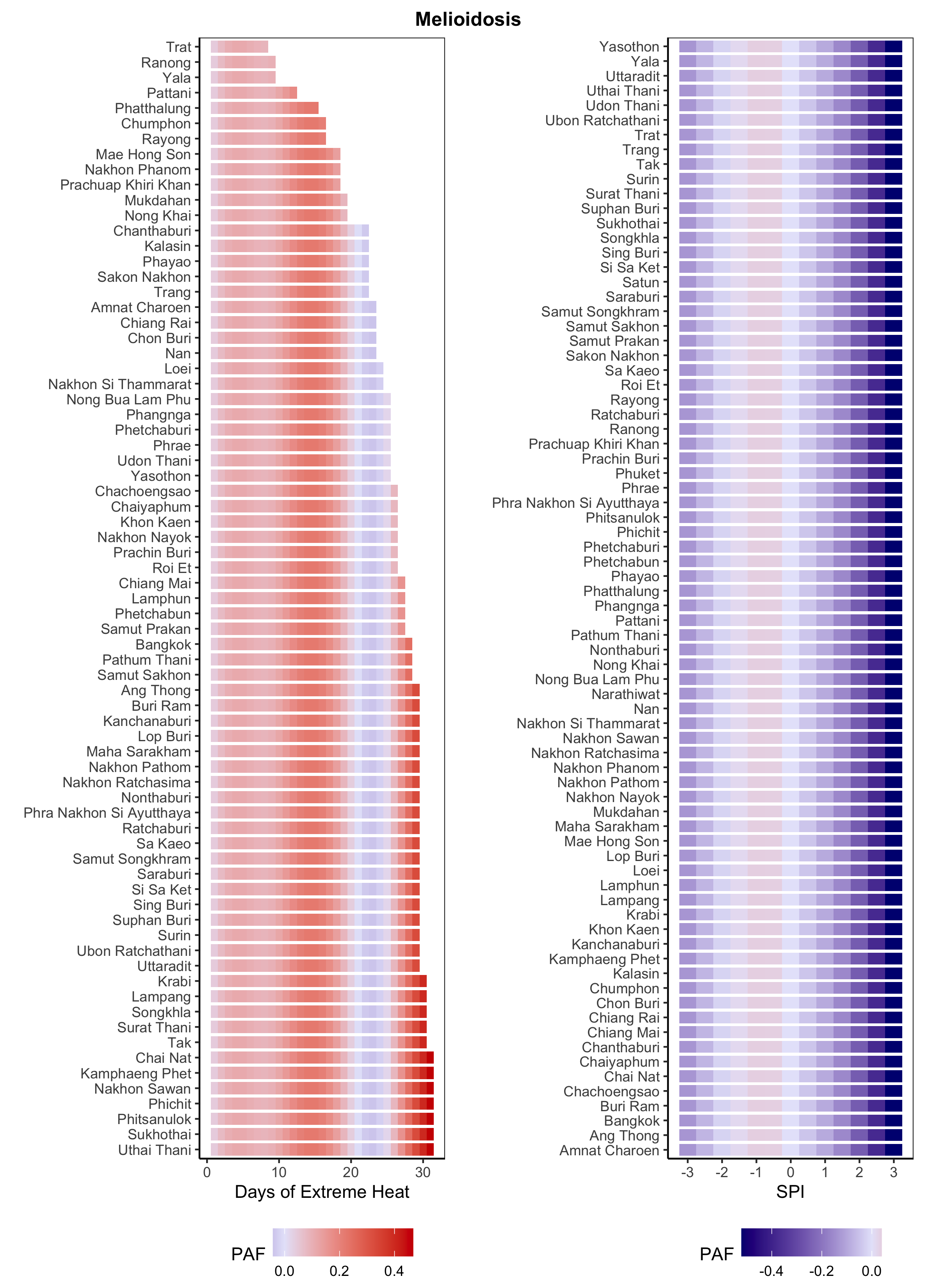

Supplement: S23 Fig — The population attributable fraction (PAF) gives the proportion of cases in a population that is attributed to a particular risk factor. A PAF of 0 indicates that the risk factor has no impact on the occurrence of the disease, and a negative PAF indicates that the factor has a protective effect against the disease. The province-specific PAF was estimated with the ratio of predicted cases when varying either SPI or extreme heat days from zero, with other covariates held constant, against predicted cases when SPI and extreme heat days are set to zero. Predicted cases were obtained from disease-specific generalised additive models. (PNG) [file pntd.0013896.s029.png]

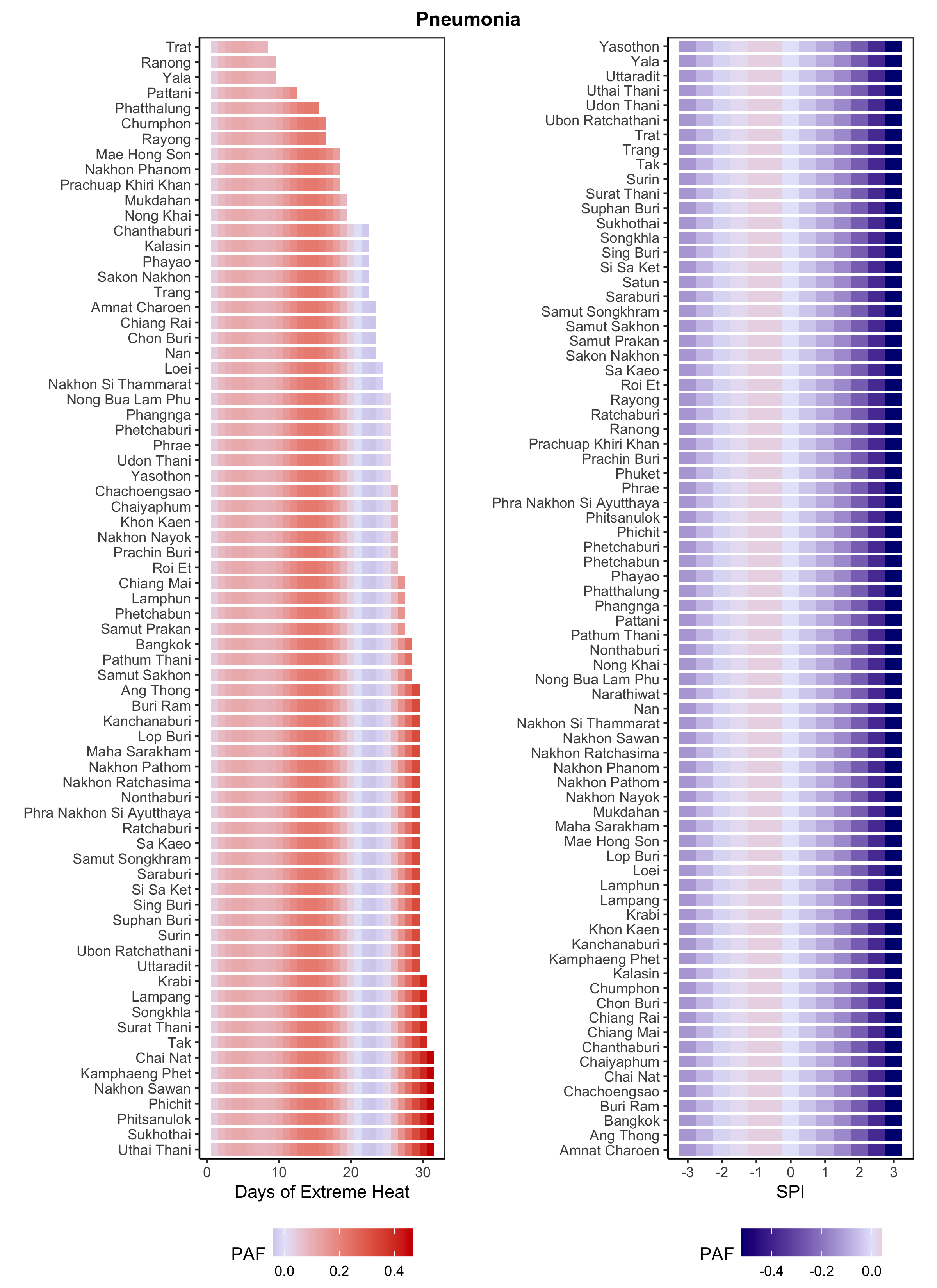

Supplement: S24 Fig — The population attributable fraction (PAF) gives the proportion of cases in a population that is attributed to a particular risk factor. A PAF of 0 indicates that the risk factor has no impact on the occurrence of the disease, and a negative PAF indicates that the factor has a protective effect against the disease. The province-specific PAF was estimated with the ratio of predicted cases when varying either SPI or extreme heat days from zero, with other covariates held constant, against predicted cases when SPI and extreme heat days are set to zero. Predicted cases were obtained from disease-specific generalised additive models. (PNG) [file pntd.0013896.s030.png]

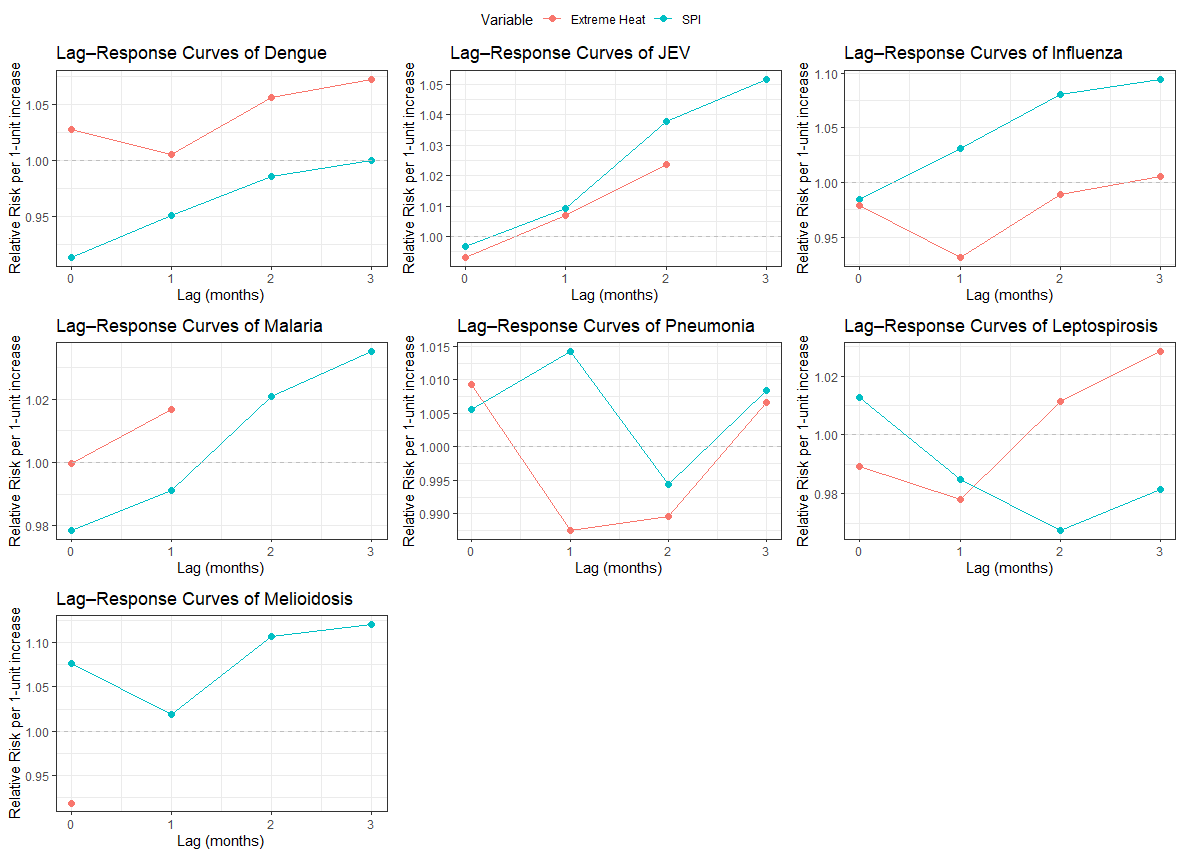

Supplement: S25 Fig — Relative risk (RR) represents the multiplicative change in disease incidence associated with a 1-unit increase in SPI or extreme heat at each lag compared to when all covariates are held at the mean. (PNG) [file pntd.0013896.s031.png]

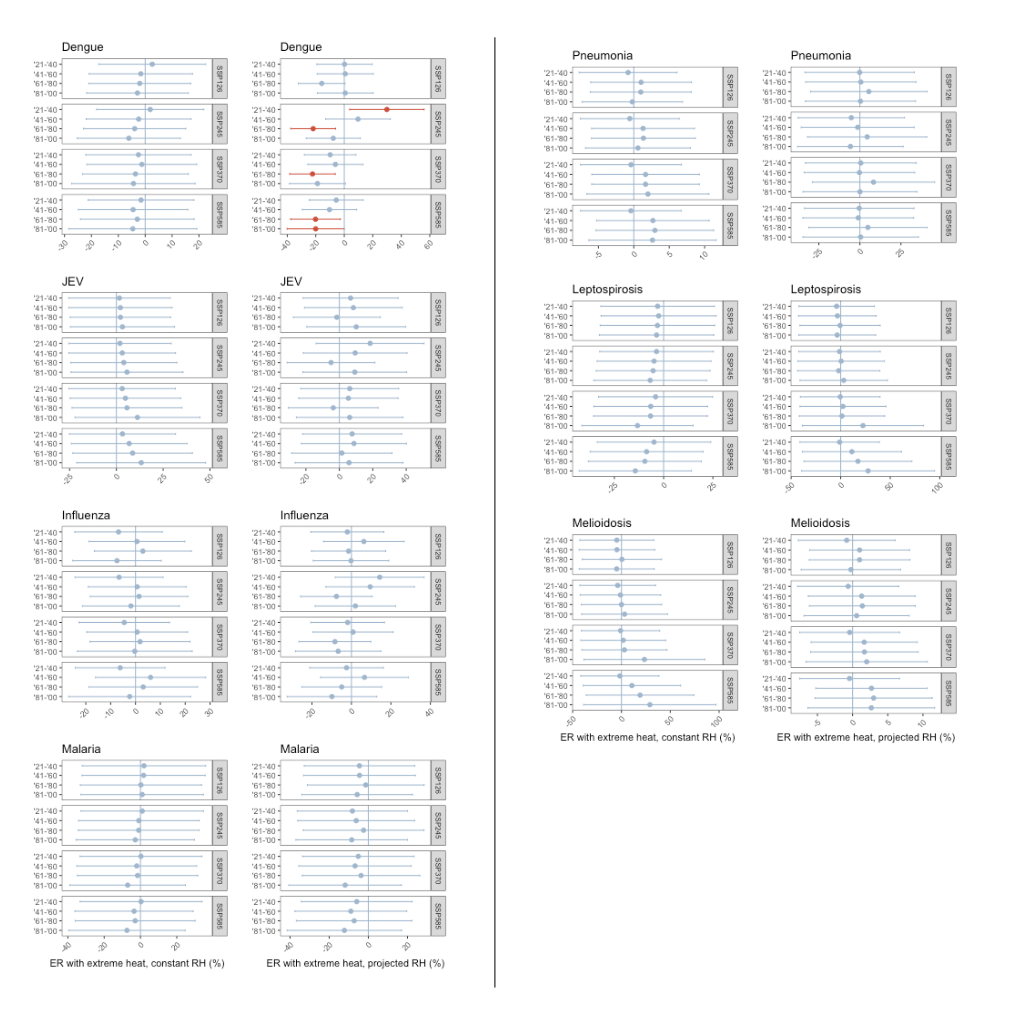

Supplement: S26 Fig — Excess risk associated with extreme heat under scenarios holding relative humidity constant (RH) (left panels) and using projected RH (right panels) for seven infectious diseases. Estimates are shown across climate change scenarios and time periods. Excess risk represents the percentage change in disease cases compared to historical levels. Holding RH constant results in minimal and non-significant dengue excess risk during extreme heat, suggesting that RH drives projected changes in dengue risk during periods of extreme heat. (PNG) [file pntd.0013896.s032.png]

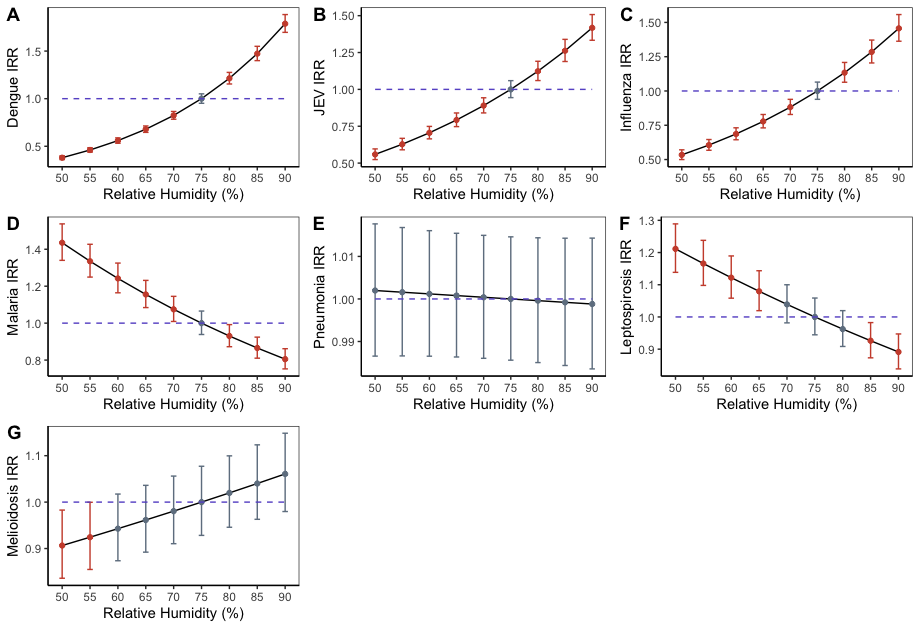

Supplement: S27 Fig — Fig shows incidence rate ratio (IRR) of each disease over relative humidity (RH). The IRR gives the ratio of predicted cases with the respective RH to predicted cases with mean RH of 75%, obtained from the disease-specific generalised additive models. An IRR above 1 represents increased incidence rate when relative humidity exceeds the mean, while an IRR below 1 represents decreased incidence rate. Points represent point estimates for the IRR with accompanying 95% confidence intervals. IRRs were derived using the ratio of predicted cases with exposure and predicted cases with mean exposure. Statistical significance was denoted with orange points when 95% CIs of the IRRs do not cross 1. (PNG) [file pntd.0013896.s033.png]

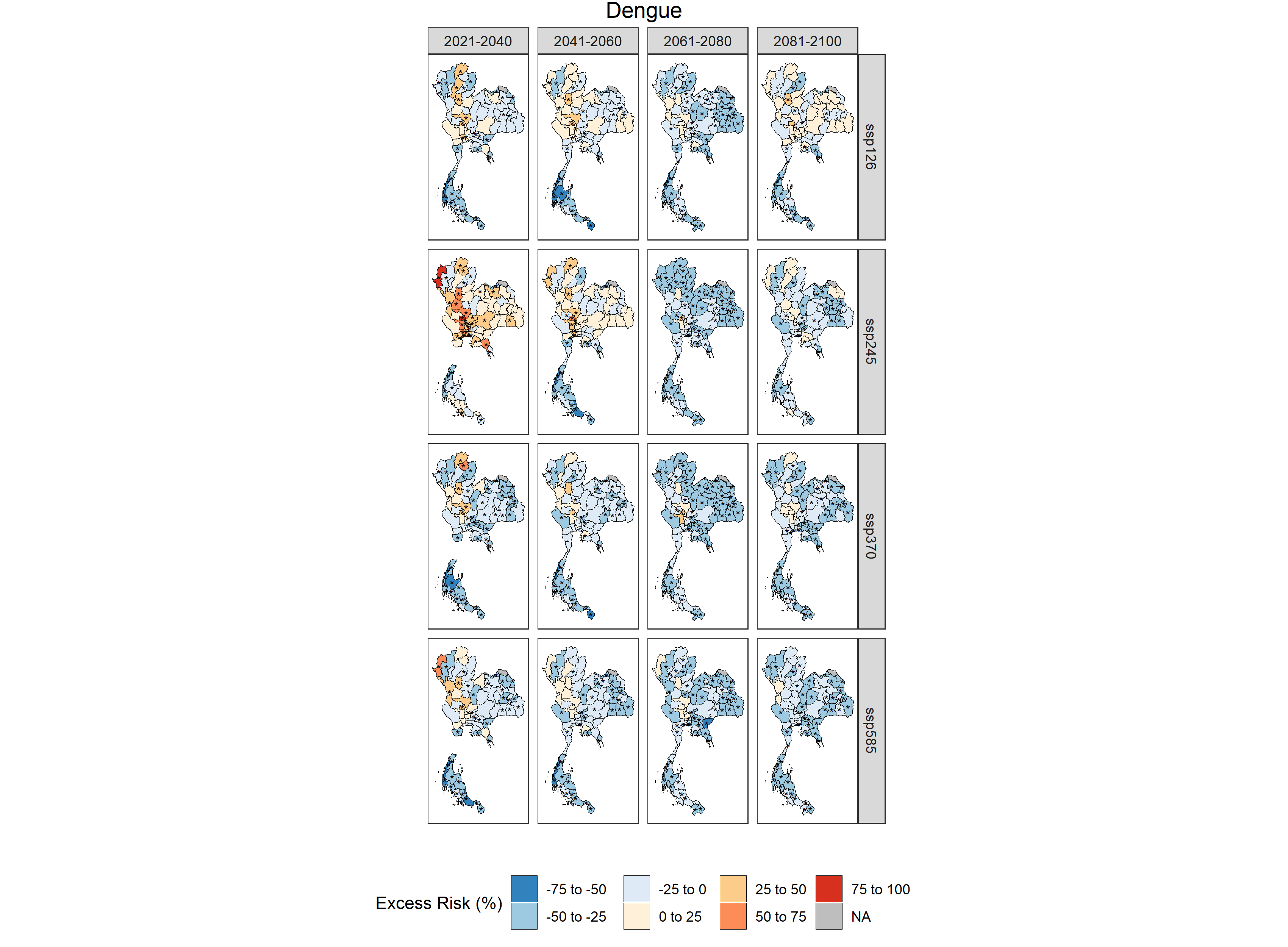

Supplement: S28 Fig — Disease-specific generalised additive models were trained using historical data and future disease cases were projected based on MIROC6 general circulation model climate data. Province-level excess risk was calculated using the mean disease case counts across the historical period and the projected case counts at a respective time period and climate change scenario at each province. Excess risk represents the percentage change in disease cases compared to historical levels. Missing polygons are provinces which are not projected to experience extreme weather events in that period and climate change scenario. Asterisks represent provinces with statistically significant excess risk. Map created using GADM data (https://gadm.org/index.html, freely available for academic use). The map outlines and administrative boundaries are used with permission for academic publishing. (PNG) [file pntd.0013896.s034.png]

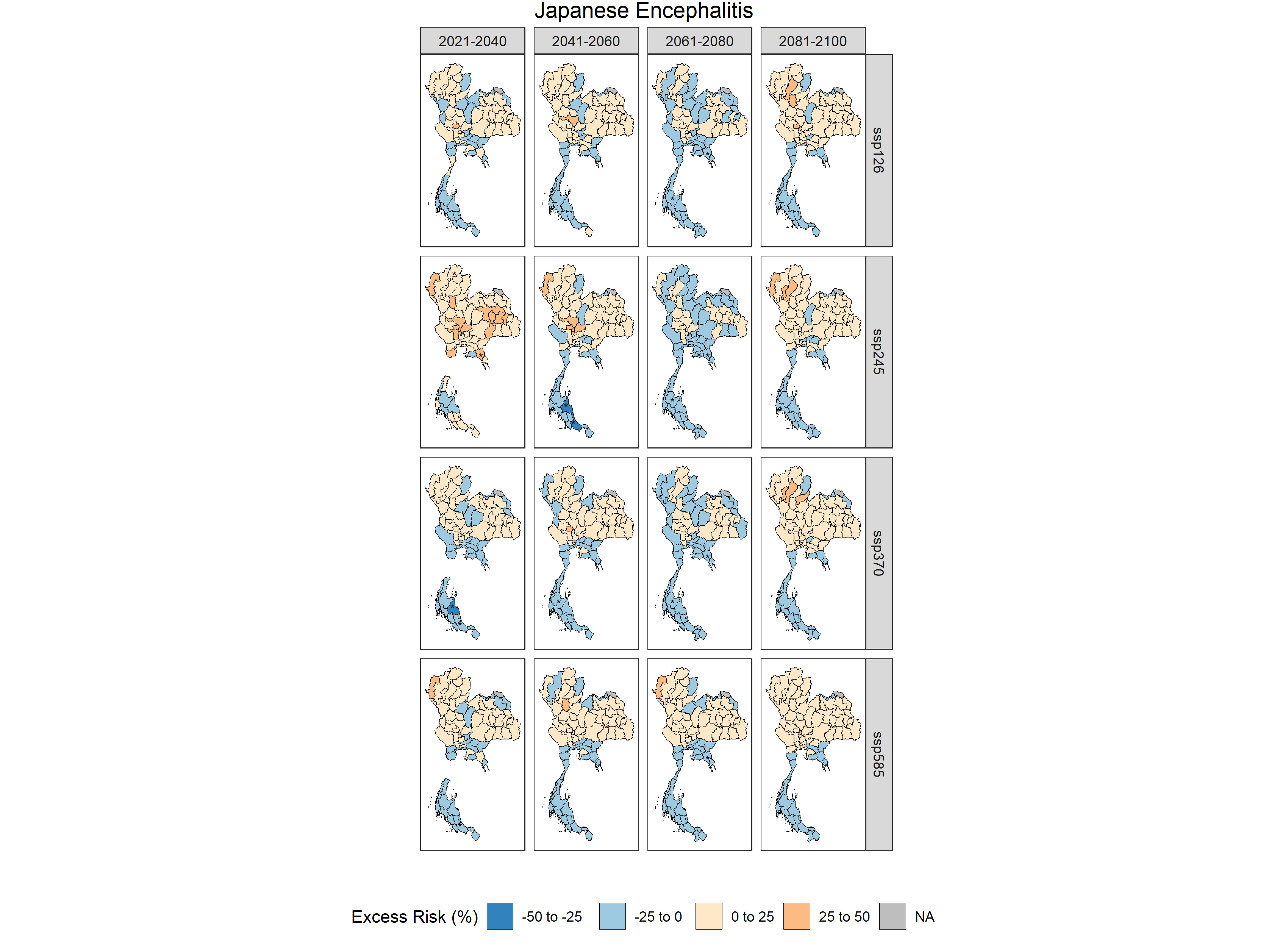

Supplement: S29 Fig — Disease-specific generalised additive models were trained using historical data and future disease cases were projected based on MIROC6 general circulation model climate data. Province-level excess risk was calculated using the mean disease case counts across the historical period and the projected case counts at a respective time period and climate change scenario at each province. Excess risk represents the percentage change in disease cases compared to historical levels. Missing polygons are provinces which are not projected to experience extreme weather events in that period and climate change scenario. Asterisks represent provinces with statistically significant excess risk. Map created using GADM data (https://gadm.org/index.html, freely available for academic use). The map outlines and administrative boundaries are used with permission for academic publishing. (PNG) [file pntd.0013896.s035.png]

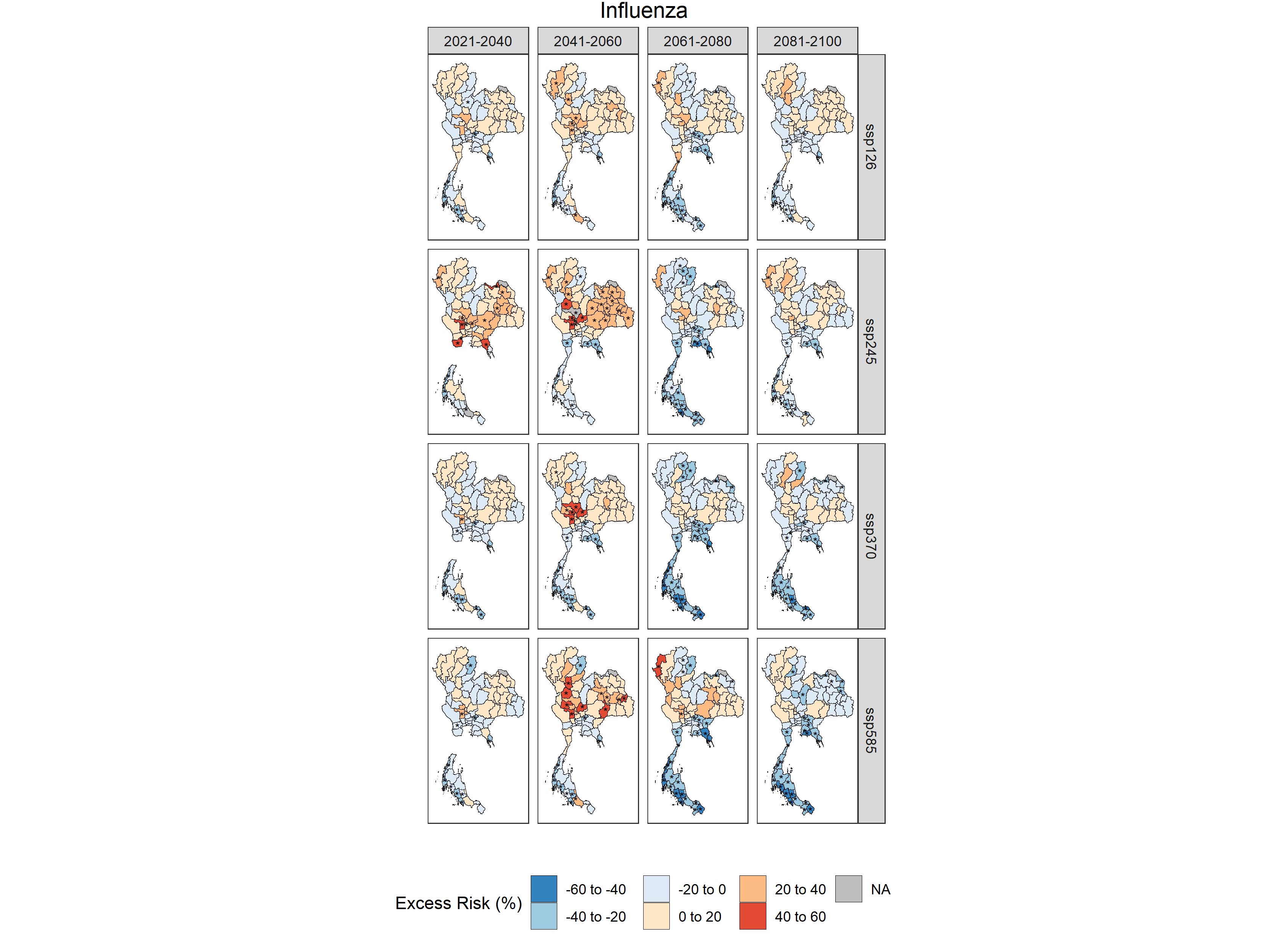

Supplement: S30 Fig — Disease-specific generalised additive models were trained using historical data and future disease cases were projected based on MIROC6 general circulation model climate data. Province-level excess risk was calculated using the mean disease case counts across the historical period and the projected case counts at a respective time period and climate change scenario at each province. Excess risk represents the percentage change in disease cases compared to historical levels. Missing polygons are provinces which are not projected to experience extreme weather events in that period and climate change scenario. Asterisks represent provinces with statistically significant excess risk. Map created using GADM data (https://gadm.org/index.html, freely available for academic use). The map outlines and administrative boundaries are used with permission for academic publishing. (PNG) [file pntd.0013896.s036.png]

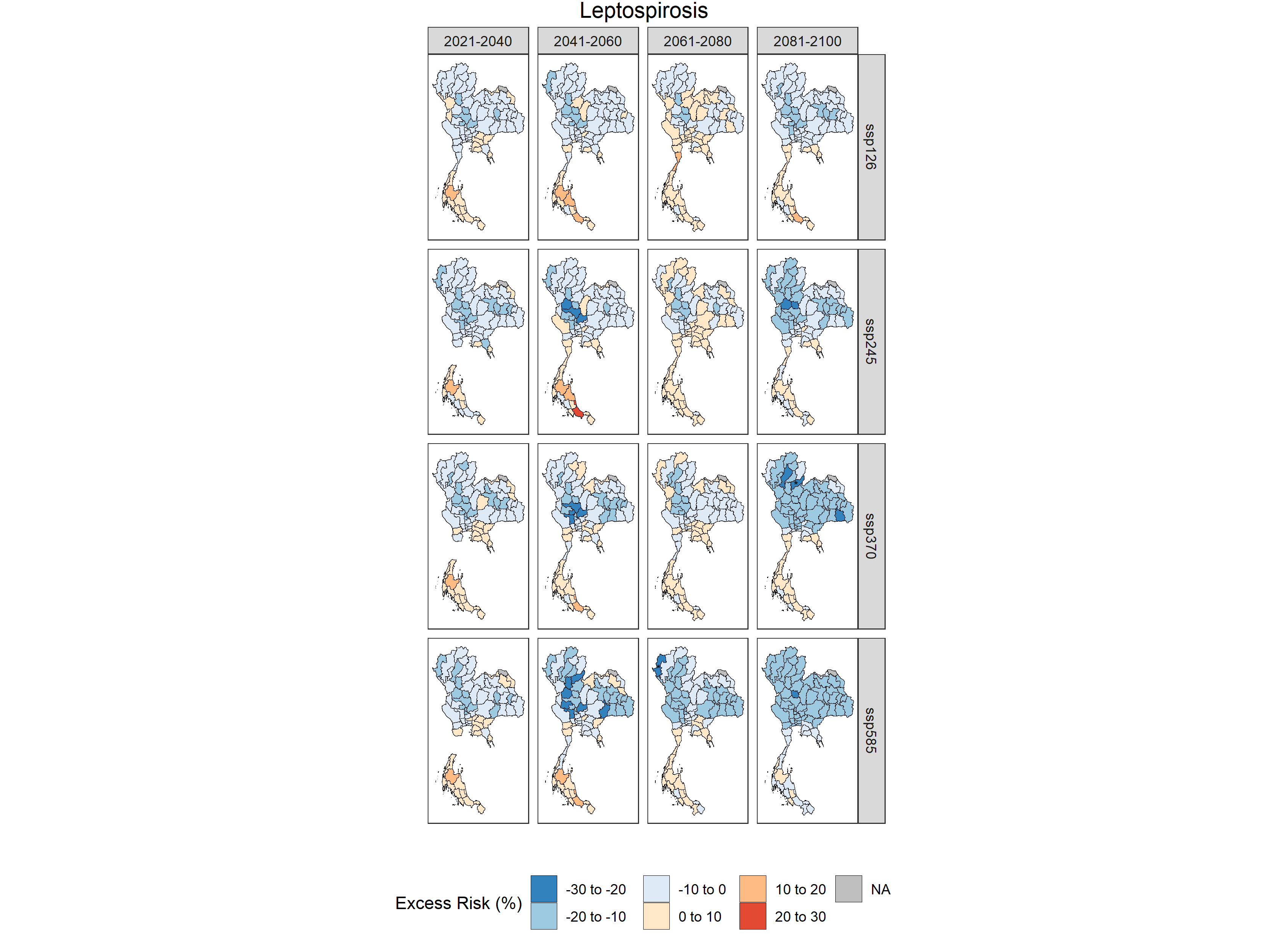

Supplement: S31 Fig — Disease-specific generalised additive models were trained using historical data and future disease cases were projected based on MIROC6 general circulation model climate data. Province-level excess risk was calculated using the mean disease case counts across the historical period and the projected case counts at a respective time period and climate change scenario at each province. Excess risk represents the percentage change in disease cases compared to historical levels. Missing polygons are provinces which are not projected to experience extreme weather events in that period and climate change scenario. Asterisks represent provinces with statistically significant excess risk. Map created using GADM data (https://gadm.org/index.html, freely available for academic use). The map outlines and administrative boundaries are used with permission for academic publishing. (PNG) [file pntd.0013896.s037.png]

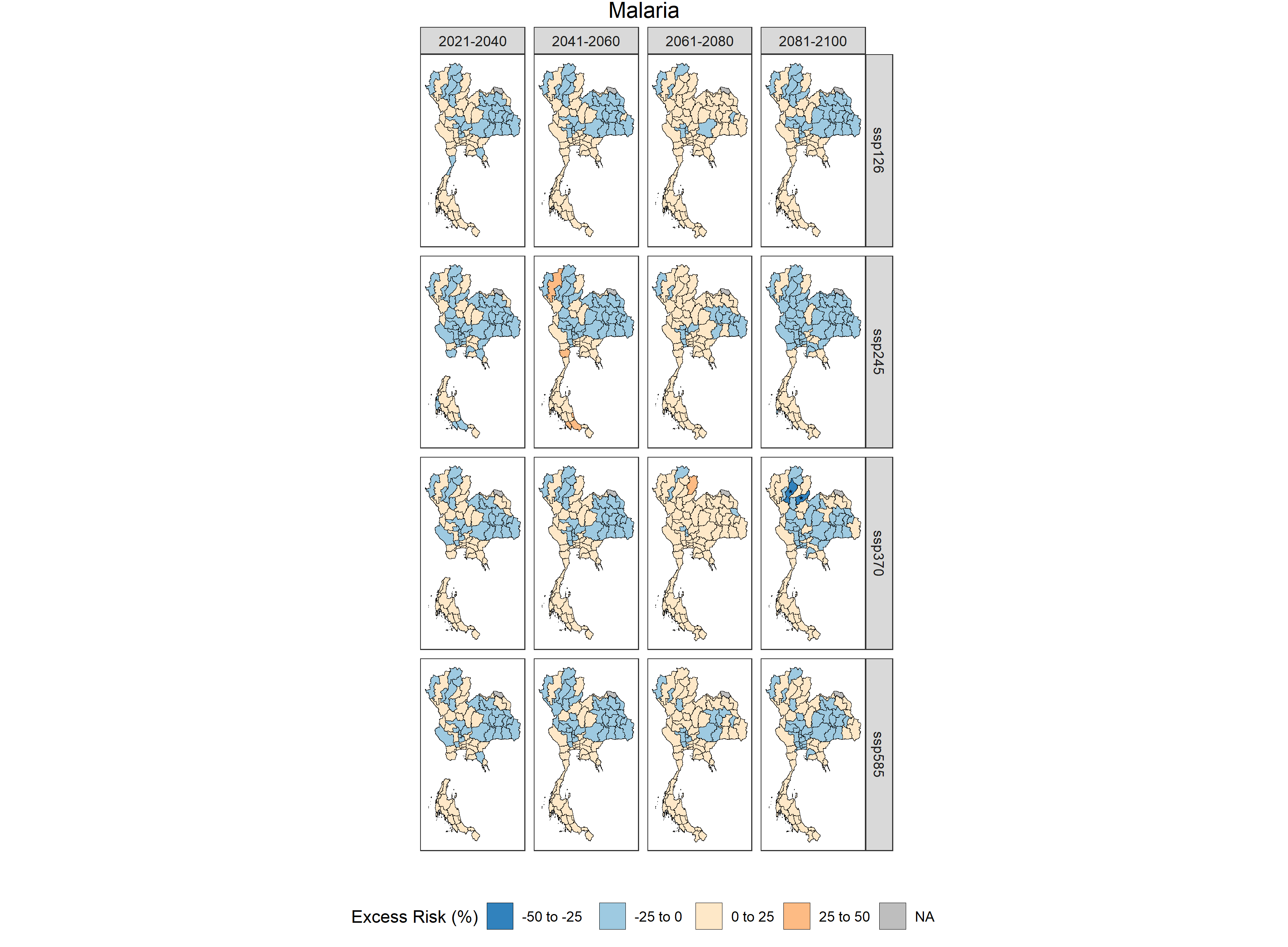

Supplement: S32 Fig — Disease-specific generalised additive models were trained using historical data and future disease cases were projected based on MIROC6 general circulation model climate data. Province-level excess risk was calculated using the mean disease case counts across the historical period and the projected case counts at a respective time period and climate change scenario at each province. Excess risk represents the percentage change in disease cases compared to historical levels. Missing polygons are provinces which are not projected to experience extreme weather events in that period and climate change scenario. Asterisks represent provinces with statistically significant excess risk. Map created using GADM data (https://gadm.org/index.html, freely available for academic use). The map outlines and administrative boundaries are used with permission for academic publishing. (PNG) [file pntd.0013896.s038.png]

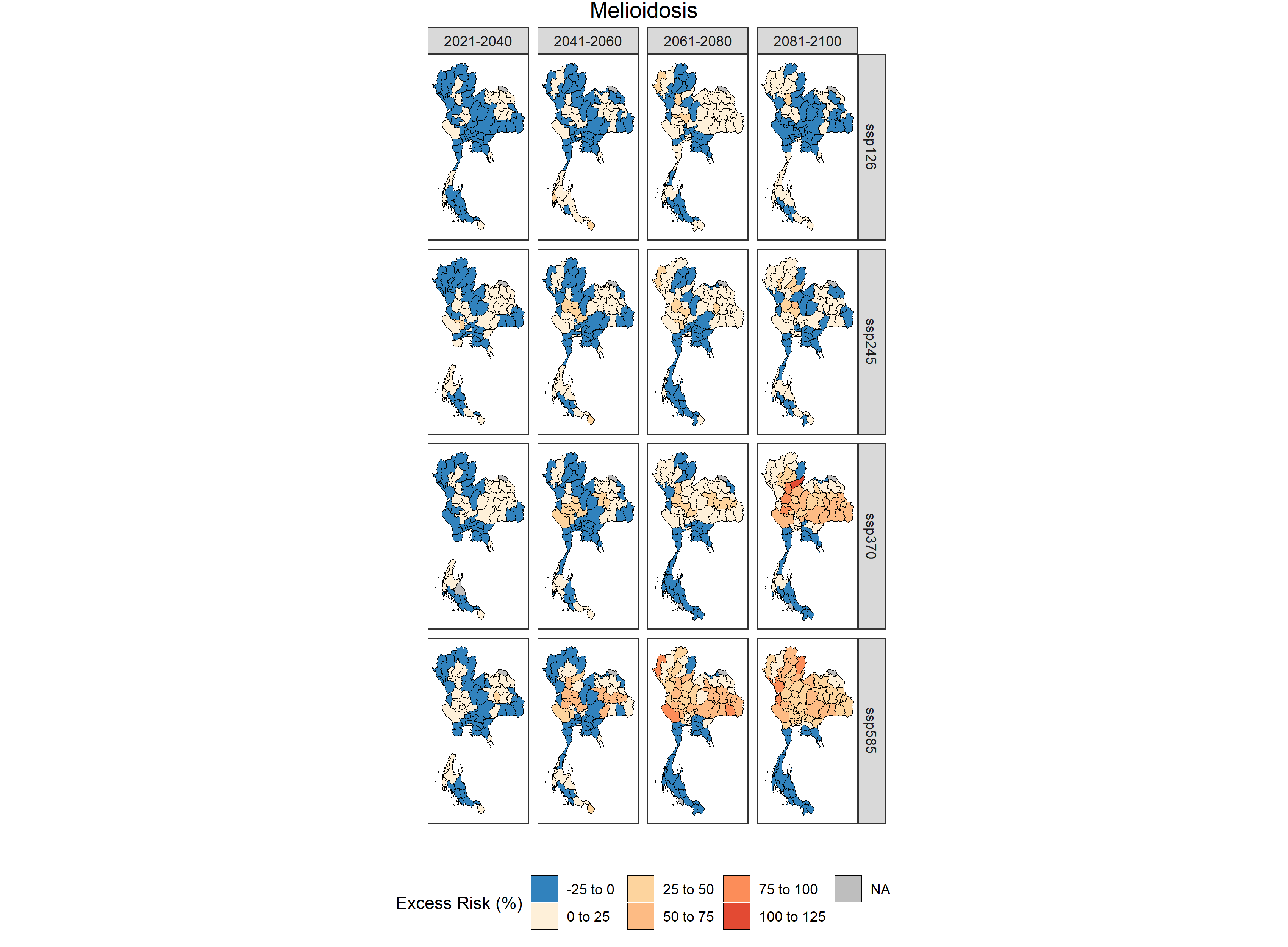

Supplement: S33 Fig — Disease-specific generalised additive models were trained using historical data and future disease cases were projected based on MIROC6 general circulation model climate data. Province-level excess risk was calculated using the mean disease case counts across the historical period and the projected case counts at a respective time period and climate change scenario at each province. Excess risk represents the percentage change in disease cases compared to historical levels. Missing polygons are provinces which are not projected to experience extreme weather events in that period and climate change scenario. Asterisks represent provinces with statistically significant excess risk. Map created using GADM data (https://gadm.org/index.html, freely available for academic use). The map outlines and administrative boundaries are used with permission for academic publishing. (PNG) [file pntd.0013896.s039.png]

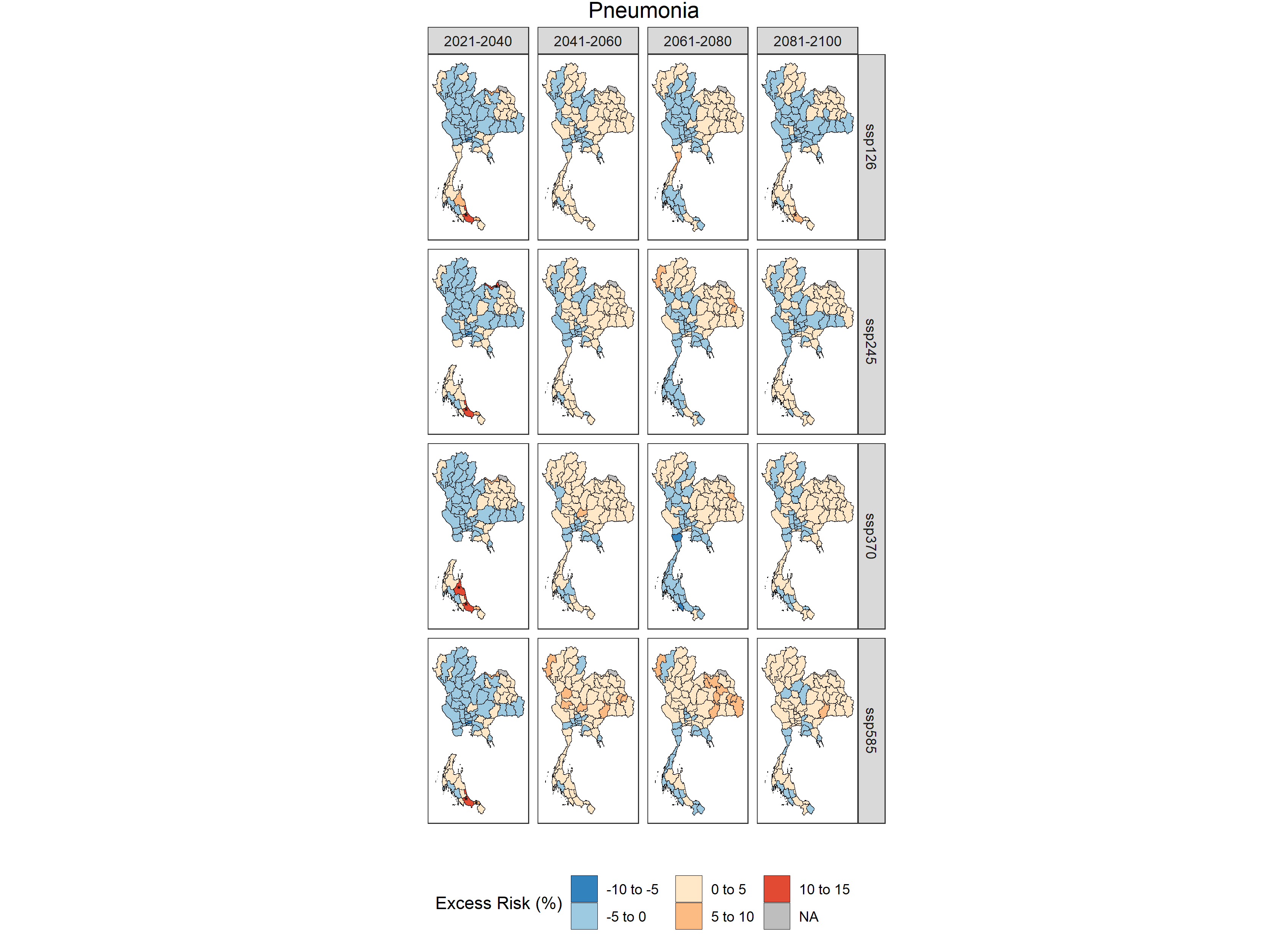

Supplement: S34 Fig — Disease-specific generalised additive models were trained using historical data and future disease cases were projected based on MIROC6 general circulation model climate data. Province-level excess risk was calculated using the mean disease case counts across the historical period and the projected case counts at a respective time period and climate change scenario at each province. Excess risk represents the percentage change in disease cases compared to historical levels. Missing polygons are provinces which are not projected to experience extreme weather events in that period and climate change scenario. Asterisks represent provinces with statistically significant excess risk. Map created using GADM data (https://gadm.org/index.html, freely available for academic use). The map outlines and administrative boundaries are used with permission for academic publishing. (PNG) [file pntd.0013896.s040.png]

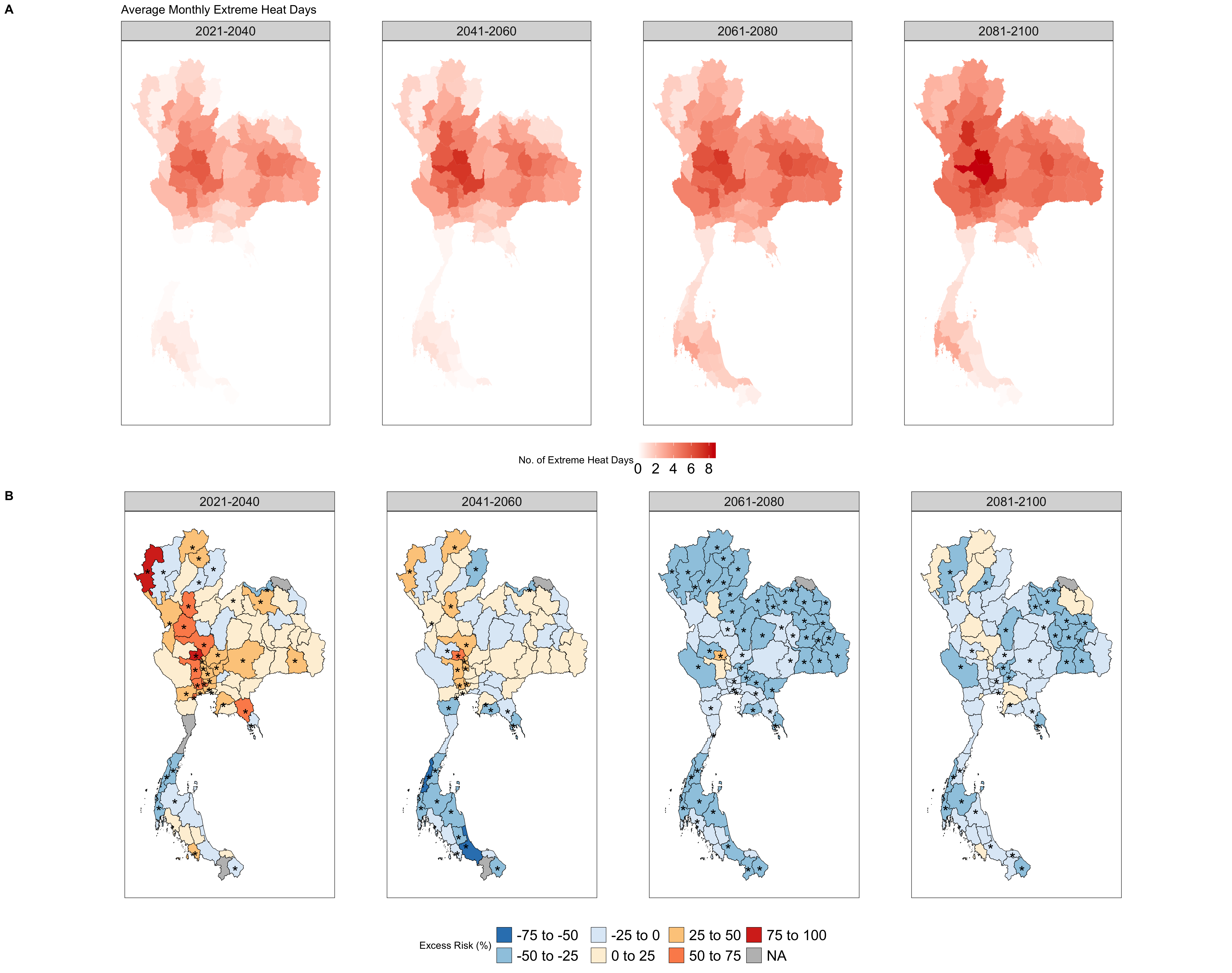

Supplement: S35 Fig — (A) Average number of extreme heat days in a month by province from 2021-2100. (B) Province-level excess risks of dengue under SSP245 from 2021–2100. Asterisks indicate a statistically significant excess risk value. Excess risk represents the percentage change in annual disease case counts from the historical baseline from 2003 to 2020. Map created using GADM data (https://gadm.org/index.html, freely available for academic use). The map outlines and administrative boundaries are used with permission for academic publishing. (PNG) [file pntd.0013896.s041.png]

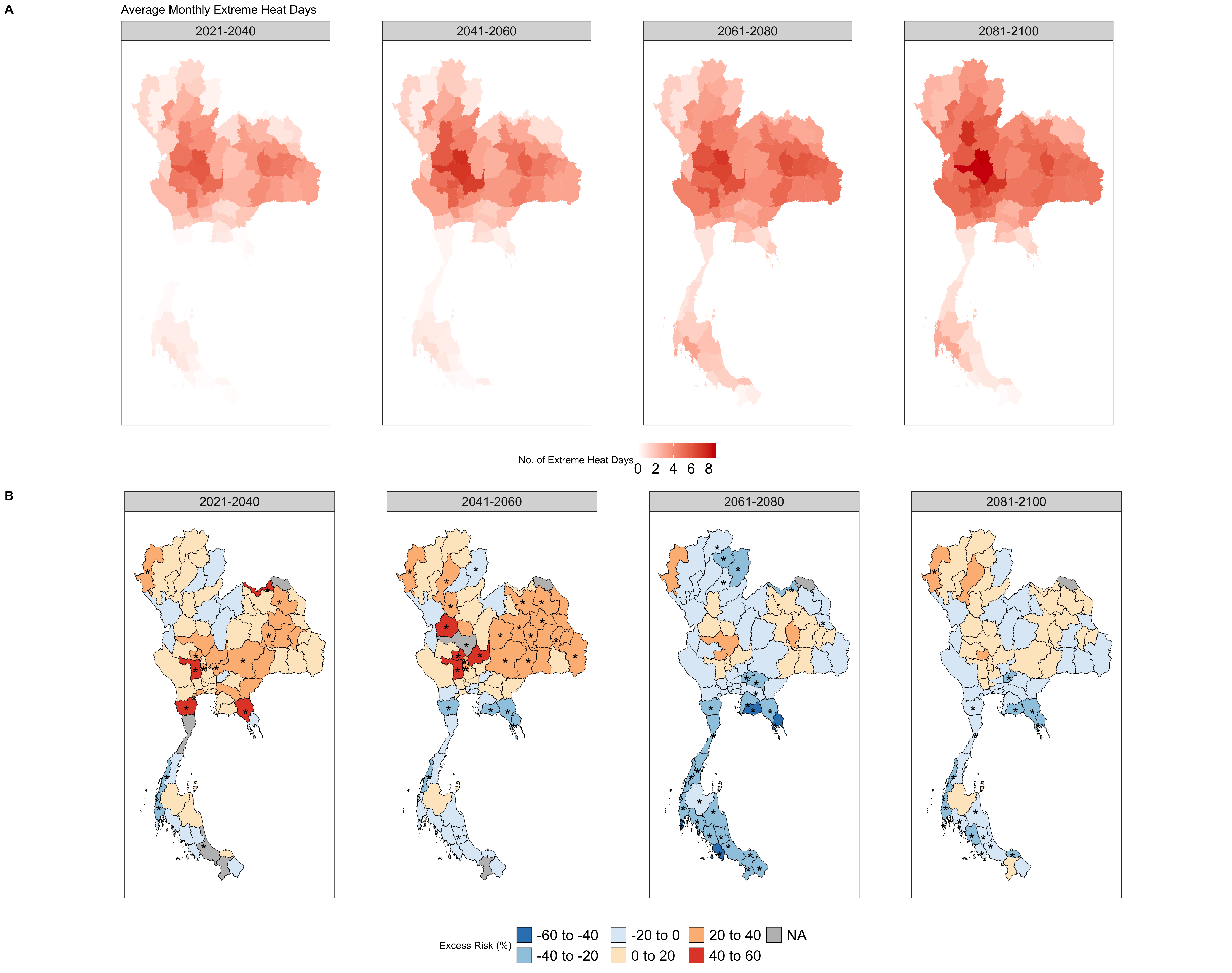

Supplement: S36 Fig — (A) Average number of extreme heat days in a month by province from 2021-2100. (B) Province-level excess risks of dengue under SSP245 from 2021–2100. Asterisks indicate a statistically significant excess risk value. Excess risk represents the percentage change in annual disease case counts from the historical baseline from 2003 to 2020. Map created using GADM data (https://gadm.org/index.html, freely available for academic use). The map outlines and administrative boundaries are used with permission for academic publishing. (PNG) [file pntd.0013896.s042.png]

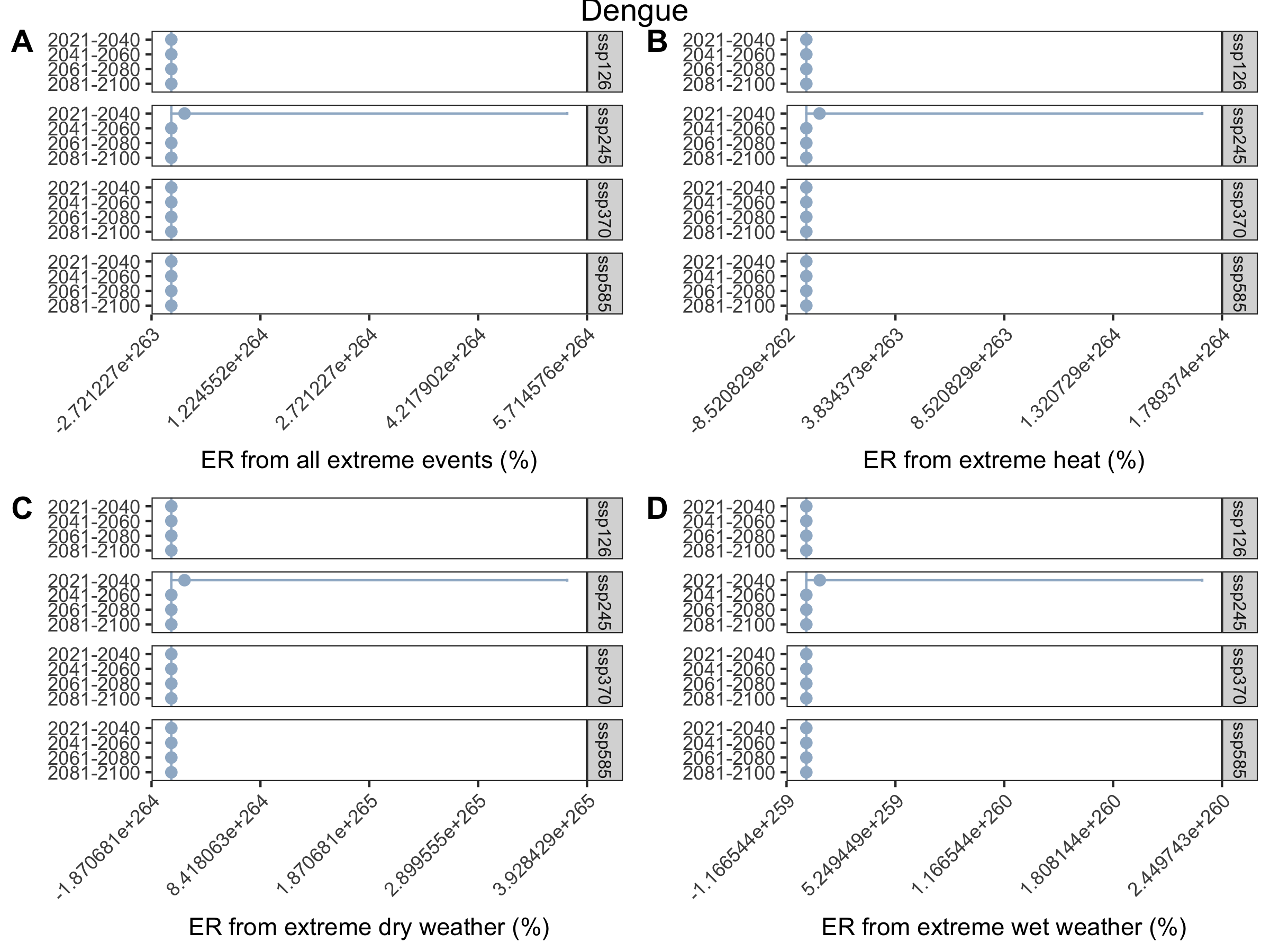

Supplement: S37 Fig — Annual excess risk of dengue in Thailand attributed to (A) all extreme weather events, (B) extreme heat days, (C) extreme dry weather and (D) extreme wet weather. Disease-specific generalized additive models (GAM) were trained with lagged extreme heat days, lagged standardized precipitation index (SPI), relative humidity and population density as variables, and population was used as an offset. Future disease cases were then projected using future extreme heat days, SPI, relative humidity from the MIROC6 general circulation model but historical population density, and historical population as an offset. In a separate analysis, the GAMs were trained on a separate historical population dataset which corresponds to the future population dataset, and we projected disease cases using future population density as a variable and future population as an offset. National-level excess risk was calculated using the mean disease case counts across the historical period, 2003–2019, and the projected case counts at a respective time period and climate change scenario at the national level. Excess risk represents the percentage change in disease cases compared to historical levels. National-level excess risks of each disease were observed to take very extreme values and hence, we did not use future population and population density when projecting disease cases. (PNG) [file pntd.0013896.s043.png]

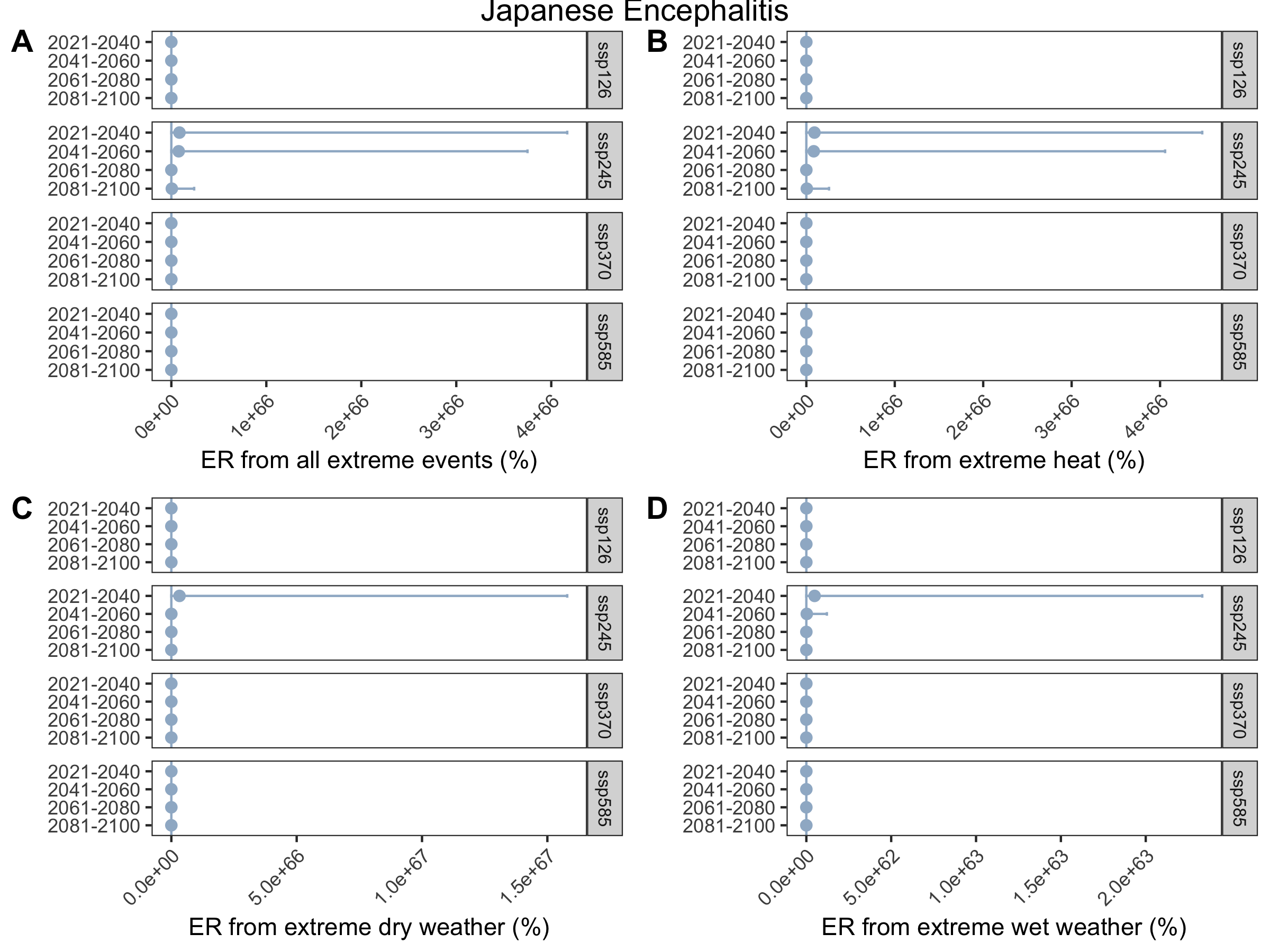

Supplement: S38 Fig — Annual excess risk of JEV in Thailand attributed to (A) all extreme weather events, (B) extreme heat days, (C) extreme dry weather and (D) extreme wet weather. Disease-specific generalized additive models (GAM) were trained with lagged extreme heat days, lagged standardized precipitation index (SPI), relative humidity and population density as variables, and population was used as an offset. Future disease cases were then projected using future extreme heat days, SPI, relative humidity from the MIROC6 general circulation model but historical population density, and historical population as an offset. In a separate analysis, the GAMs were trained on a separate historical population dataset which corresponds to the future population dataset, and we projected disease cases using future population density as a variable and future population as an offset. National-level excess risk was calculated using the mean disease case counts across the historical period, 2003–2019, and the projected case counts at a respective time period and climate change scenario at the national level. Excess risk represents the percentage change in disease cases compared to historical levels. National-level excess risks of each disease were observed to take very extreme values and hence, we did not use future population and population density when projecting disease cases. (PNG) [file pntd.0013896.s044.png]

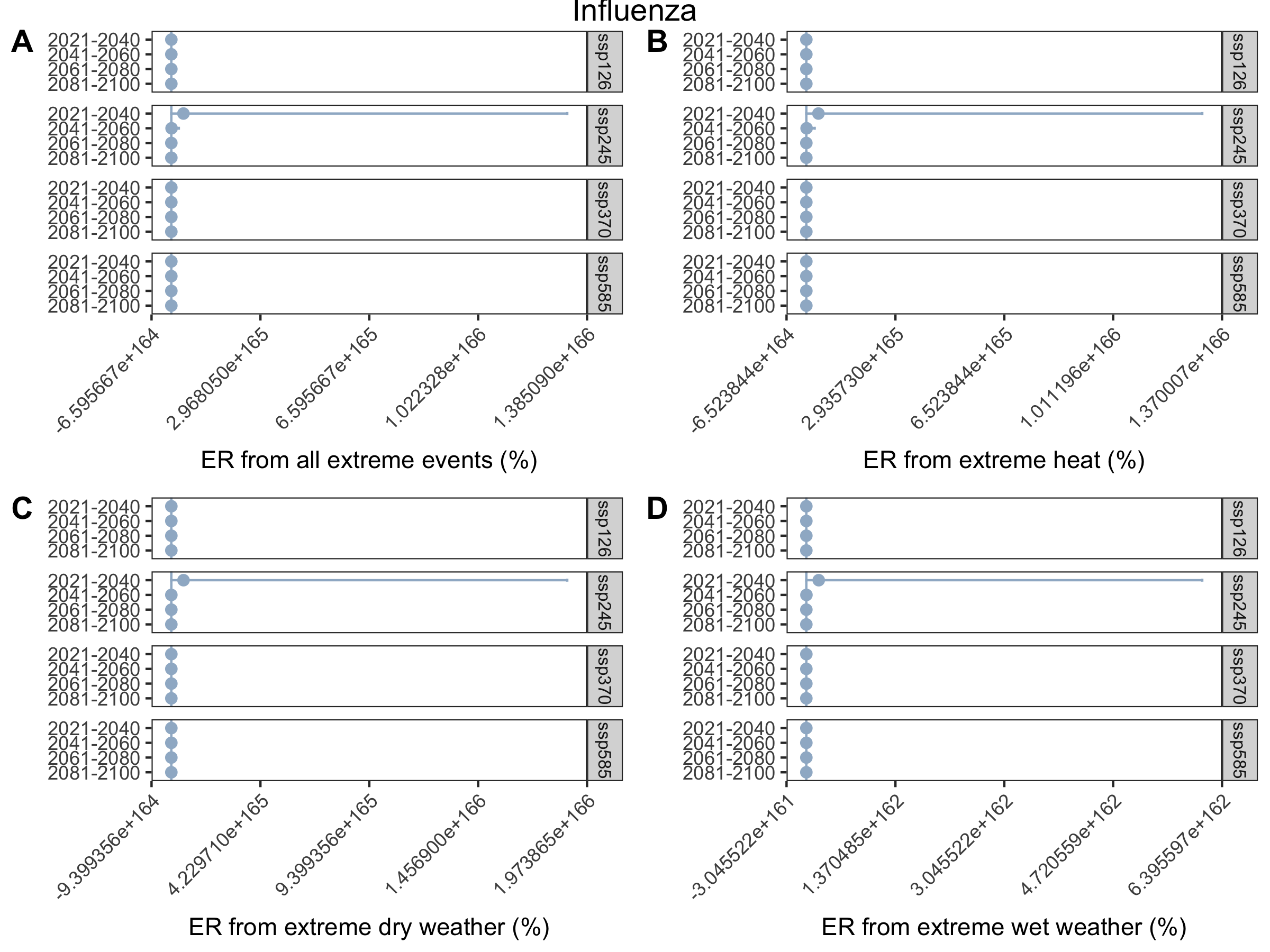

Supplement: S39 Fig — Annual excess risk of influenza in Thailand attributed to (A) all extreme weather events, (B) extreme heat days, (C) extreme dry weather and (D) extreme wet weather. Missing values are due to insufficient data in that period. Disease-specific generalized additive models (GAM) were trained with lagged extreme heat days, lagged standardized precipitation index (SPI), relative humidity and population density as variables, and population was used as an offset. Future disease cases were then projected using future extreme heat days, SPI, relative humidity from the MIROC6 general circulation model but historical population density, and historical population as an offset. In a separate analysis, the GAMs were trained on a separate historical population dataset which corresponds to the future population dataset, and we projected disease cases using future population density as a variable and future population as an offset. National-level excess risk was calculated using the mean disease case counts across the historical period, 2003–2019, and the projected case counts at a respective time period and climate change scenario at the national level. Excess risk represents the percentage change in disease cases compared to historical levels. National-level excess risks of each disease were observed to take very extreme values and hence, we did not use future population and population density when projecting disease cases. (PNG) [file pntd.0013896.s045.png]

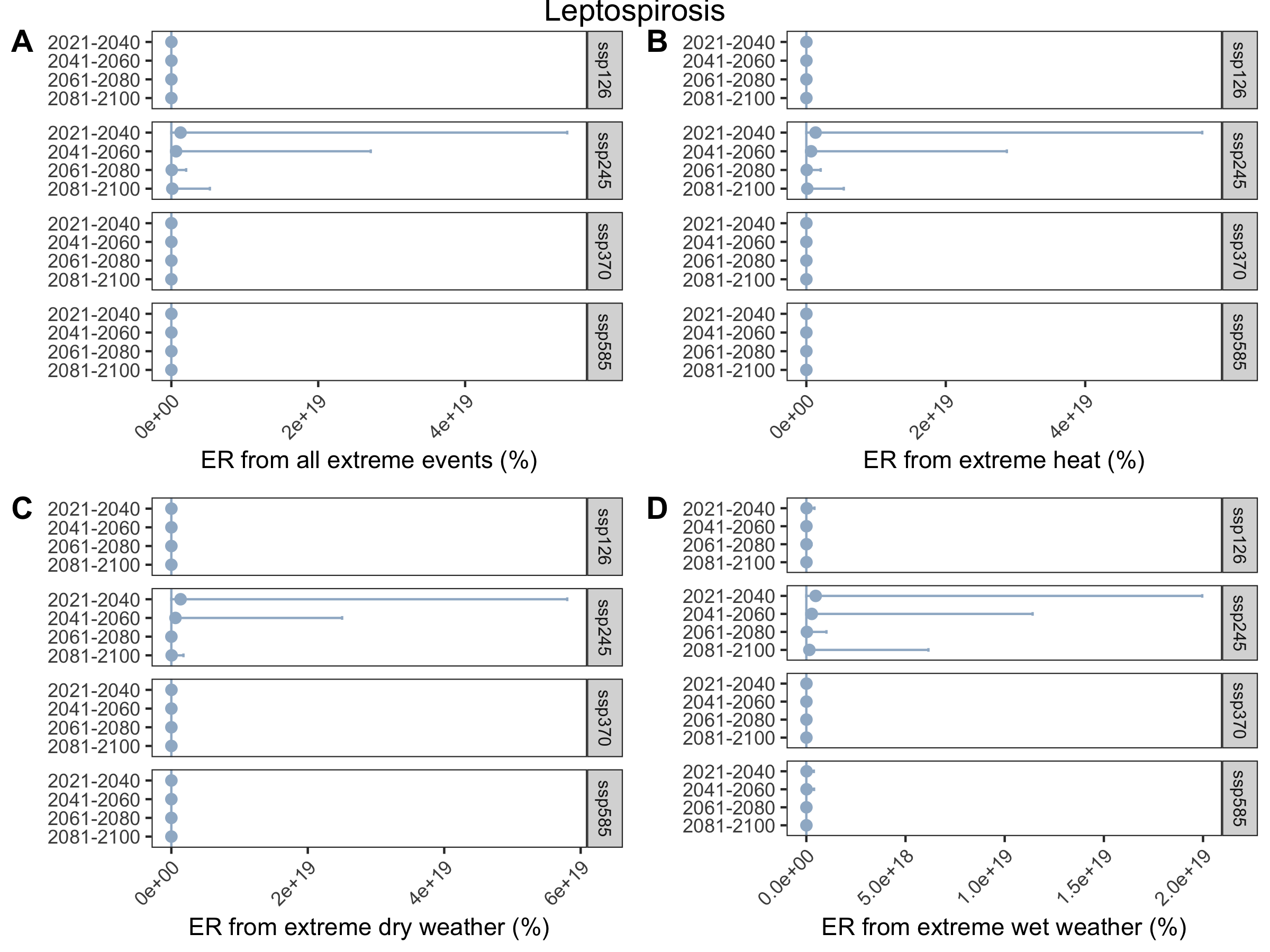

Supplement: S40 Fig — Annual excess risk of leptospirosis in Thailand attributed to (A) all extreme weather events, (B) extreme heat days, (C) extreme dry weather and (D) extreme wet weather. Disease-specific generalized additive models (GAM) were trained with lagged extreme heat days, lagged standardized precipitation index (SPI), relative humidity and population density as variables, and population was used as an offset. Future disease cases were then projected using future extreme heat days, SPI, relative humidity from the MIROC6 general circulation model but historical population density, and historical population as an offset. In a separate analysis, the GAMs were trained on a separate historical population dataset which corresponds to the future population dataset, and we projected disease cases using future population density as a variable and future population as an offset. National-level excess risk was calculated using the mean disease case counts across the historical period, 2003–2019, and the projected case counts at a respective time period and climate change scenario at the national level. Excess risk represents the percentage change in disease cases compared to historical levels. National-level excess risks of each disease were observed to take very extreme values and hence, we did not use future population and population density when projecting disease cases. (PNG) [file pntd.0013896.s046.png]

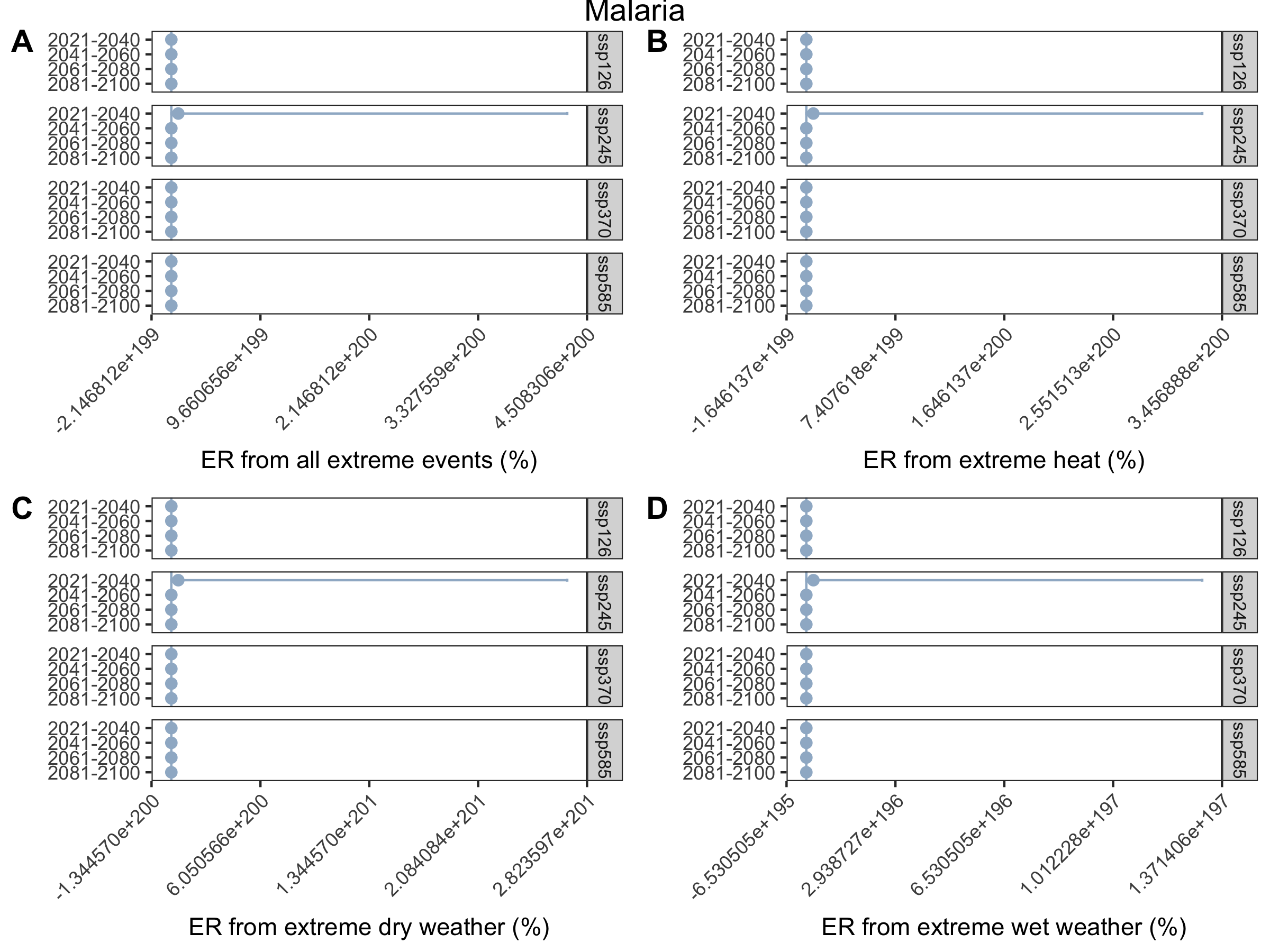

Supplement: S41 Fig — Annual excess risk of malaria in Thailand attributed to (A) all extreme weather events, (B) extreme heat days, (C) extreme dry weather and (D) extreme wet weather. Disease-specific generalized additive models (GAM) were trained with lagged extreme heat days, lagged standardized precipitation index (SPI), relative humidity and population density as variables, and population was used as an offset. Future disease cases were then projected using future extreme heat days, SPI, relative humidity from the MIROC6 general circulation model but historical population density, and historical population as an offset. In a separate analysis, the GAMs were trained on a separate historical population dataset which corresponds to the future population dataset, and we projected disease cases using future population density as a variable and future population as an offset. National-level excess risk was calculated using the mean disease case counts across the historical period, 2003–2019, and the projected case counts at a respective time period and climate change scenario at the national level. Excess risk represents the percentage change in disease cases compared to historical levels. National-level excess risks of each disease were observed to take very extreme values and hence, we did not use future population and population density when projecting disease cases. (PNG) [file pntd.0013896.s047.png]

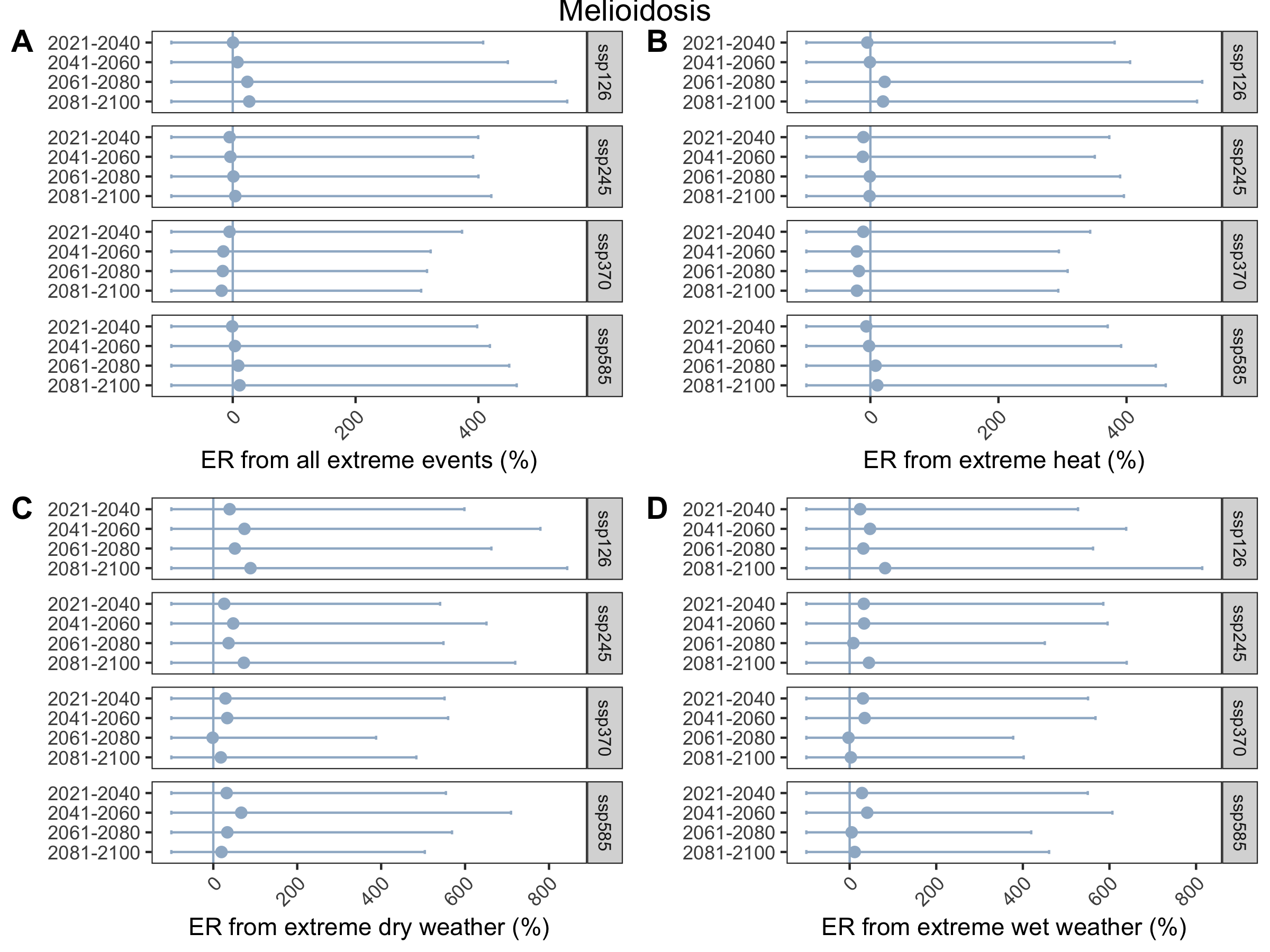

Supplement: S42 Fig — Annual excess risk of melioidosis in Thailand attributed to (A) all extreme weather events, (B) extreme heat days, (C) extreme dry weather and (D) extreme wet weather. Disease-specific generalized additive models (GAM) were trained with lagged extreme heat days, lagged standardized precipitation index (SPI), relative humidity and population density as variables, and population was used as an offset. Future disease cases were then projected using future extreme heat days, SPI, relative humidity from the MIROC6 general circulation model but historical population density, and historical population as an offset. In a separate analysis, the GAMs were trained on a separate historical population dataset which corresponds to the future population dataset, and we projected disease cases using future population density as a variable and future population as an offset. National-level excess risk was calculated using the mean disease case counts across the historical period, 2003–2019, and the projected case counts at a respective time period and climate change scenario at the national level. Excess risk represents the percentage change in disease cases compared to historical levels. National-level excess risks of each disease were observed to take very extreme values and hence, we did not use future population and population density when projecting disease cases. (PNG) [file pntd.0013896.s048.png]

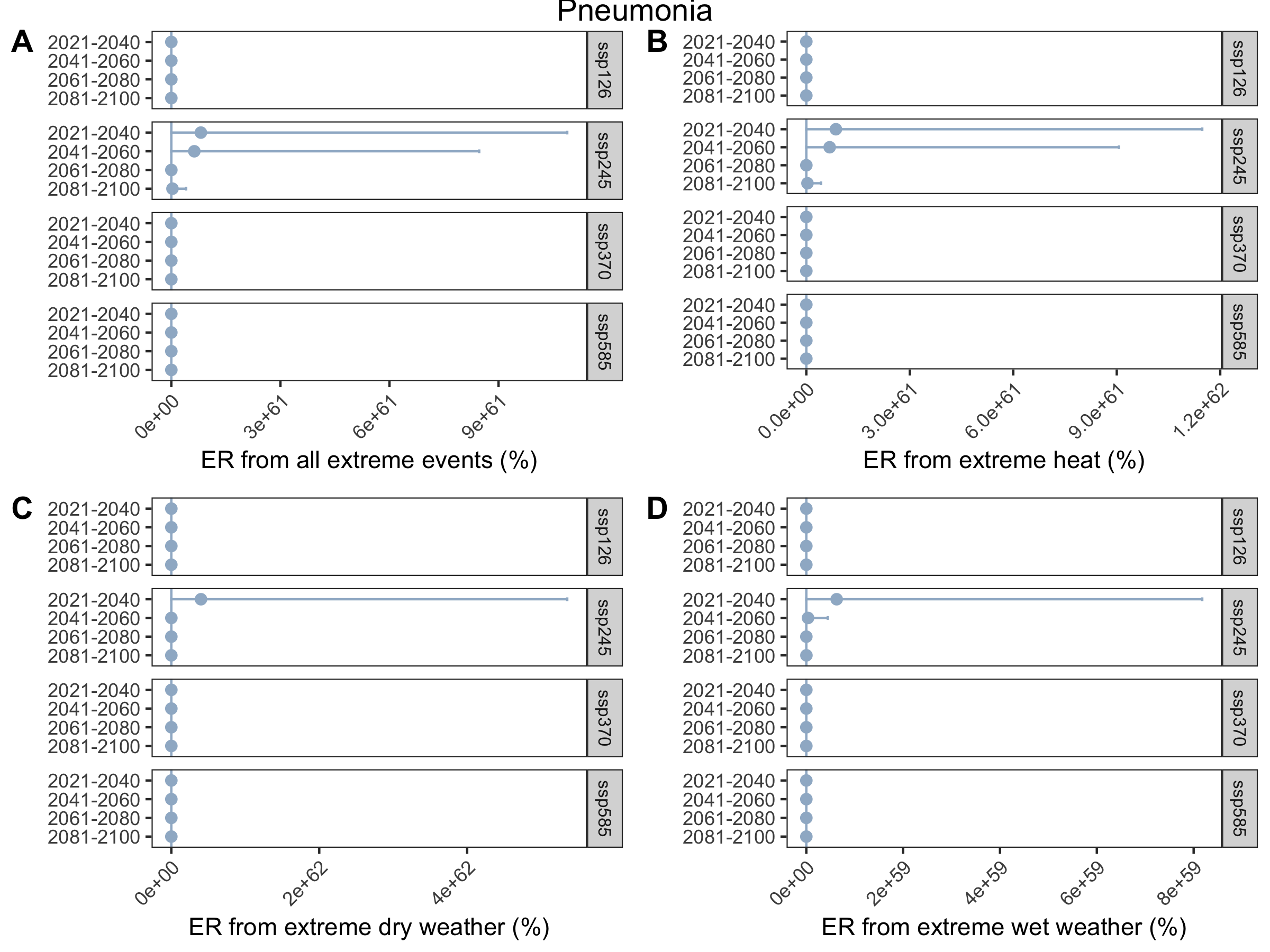

Supplement: S43 Fig — Annual excess risk of pneumonia in Thailand attributed to (A) all extreme weather events, (B) extreme heat days, (C) extreme dry weather and (D) extreme wet weather. Disease-specific generalized additive models (GAM) were trained with lagged extreme heat days, lagged standardized precipitation index (SPI), relative humidity and population density as variables, and population was used as an offset. Future disease cases were then projected using future extreme heat days, SPI, relative humidity from the MIROC6 general circulation model but historical population density, and historical population as an offset. In a separate analysis, the GAMs were trained on a separate historical population dataset which corresponds to the future population dataset, and we projected disease cases using future population density as a variable and future population as an offset. National-level excess risk was calculated using the mean disease case counts across the historical period, 2003–2019, and the projected case counts at a respective time period and climate change scenario at the national level. Excess risk represents the percentage change in disease cases compared to historical levels. National-level excess risks of each disease were observed to take very extreme values and hence, we did not use future population and population density when projecting disease cases. (PNG) [file pntd.0013896.s049.png]

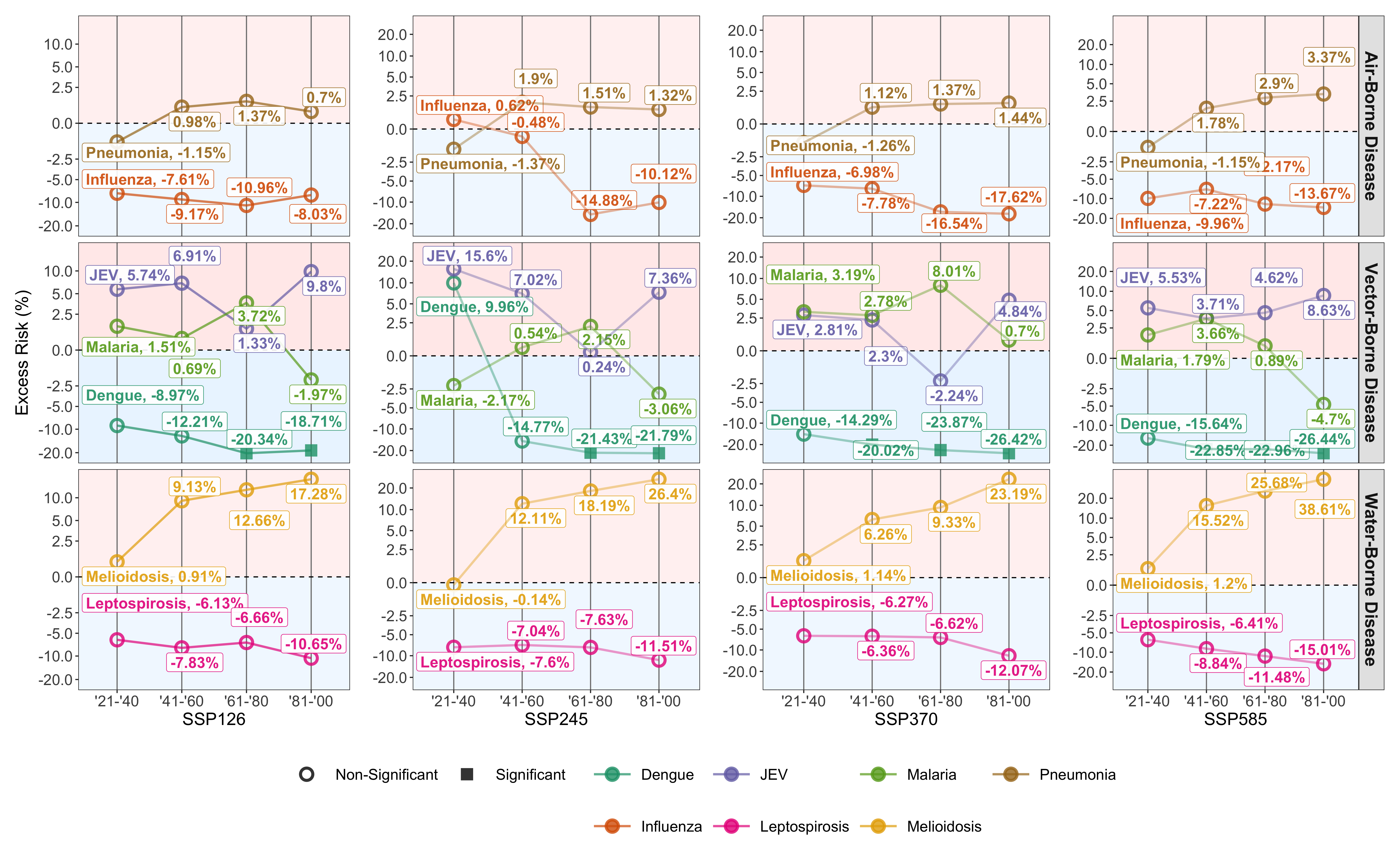

Supplement: S44 Fig — Disease-specific generalized additive models (GAM) were trained on historical data from 2003-2019 and used to project future case counts of the respective disease across 4 time periods (2021–2040, 2041–2060, 2061–2080, 2081–2100) and 4 climate change scenarios (SSP126, SSP245, SSP370, SSP585) during periods of extreme weather. Projections were made based on climate data from the CMCC-ESM2 general circulation model. National-level excess risk was calculated using the mean disease case counts across the historical period and the projected case counts at a respective time period and climate change scenario. Excess risk represents the percentage change in disease cases compared to historical levels. (PNG) [file pntd.0013896.s050.png]

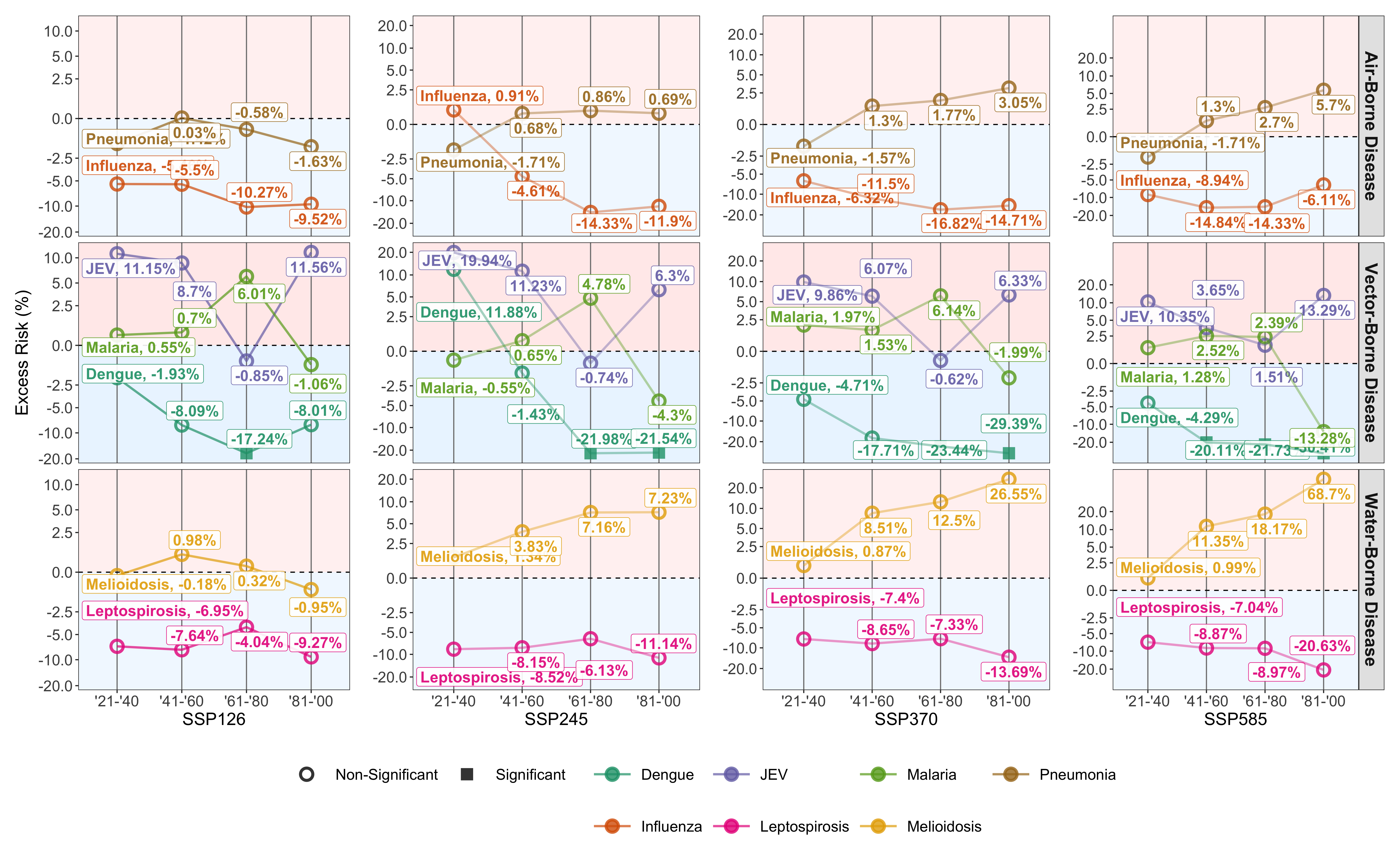

Supplement: S45 Fig — Disease-specific generalized additive models (GAM) were trained on historical data from 2003-2019 and used to project future case counts of the respective disease across 4 time periods (2021–2040, 2041–2060, 2061–2080, 2081–2100) and 4 climate change scenarios (SSP126, SSP245, SSP370, SSP585) during periods of extreme weather. Projections were made based on climate data from the IPSL-CM6A-LR general circulation model. National-level excess risk was calculated using the mean disease case counts across the historical period and the projected case counts at a respective time period and climate change scenario. Excess risk represents the percentage change in disease cases compared to historical levels. (PNG) [file pntd.0013896.s051.png]

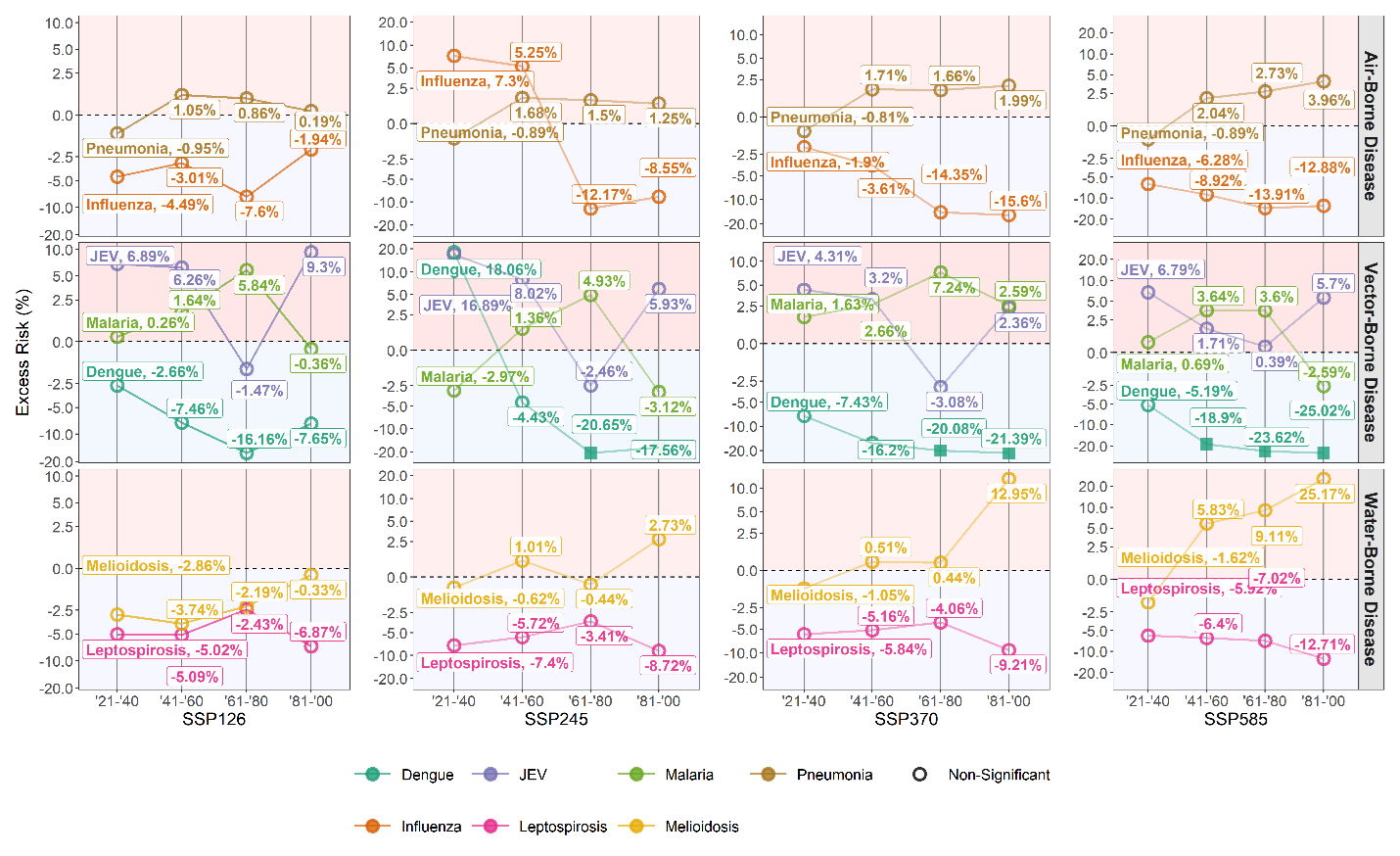

Supplement: S46 Fig — Disease-specific generalized additive models (GAM) were trained on historical data from 2003-2019 and used to project future case counts of the respective disease across 4 time periods (2021–2040, 2041–2060, 2061–2080, 2081–2100) and 4 climate change scenarios (SSP126, SSP245, SSP370, SSP585) during periods of extreme weather. Projections were made based on the means of climate data from the MIROC6, CMCC-ESM2 and IPSL-CM6A-LR general circulation model. National-level excess risk was calculated using the mean disease case counts across the historical period and the projected case counts at a respective time period and climate change scenario. Excess risk represents the percentage change in disease cases compared to historical levels. (PNG) [file pntd.0013896.s052.png]

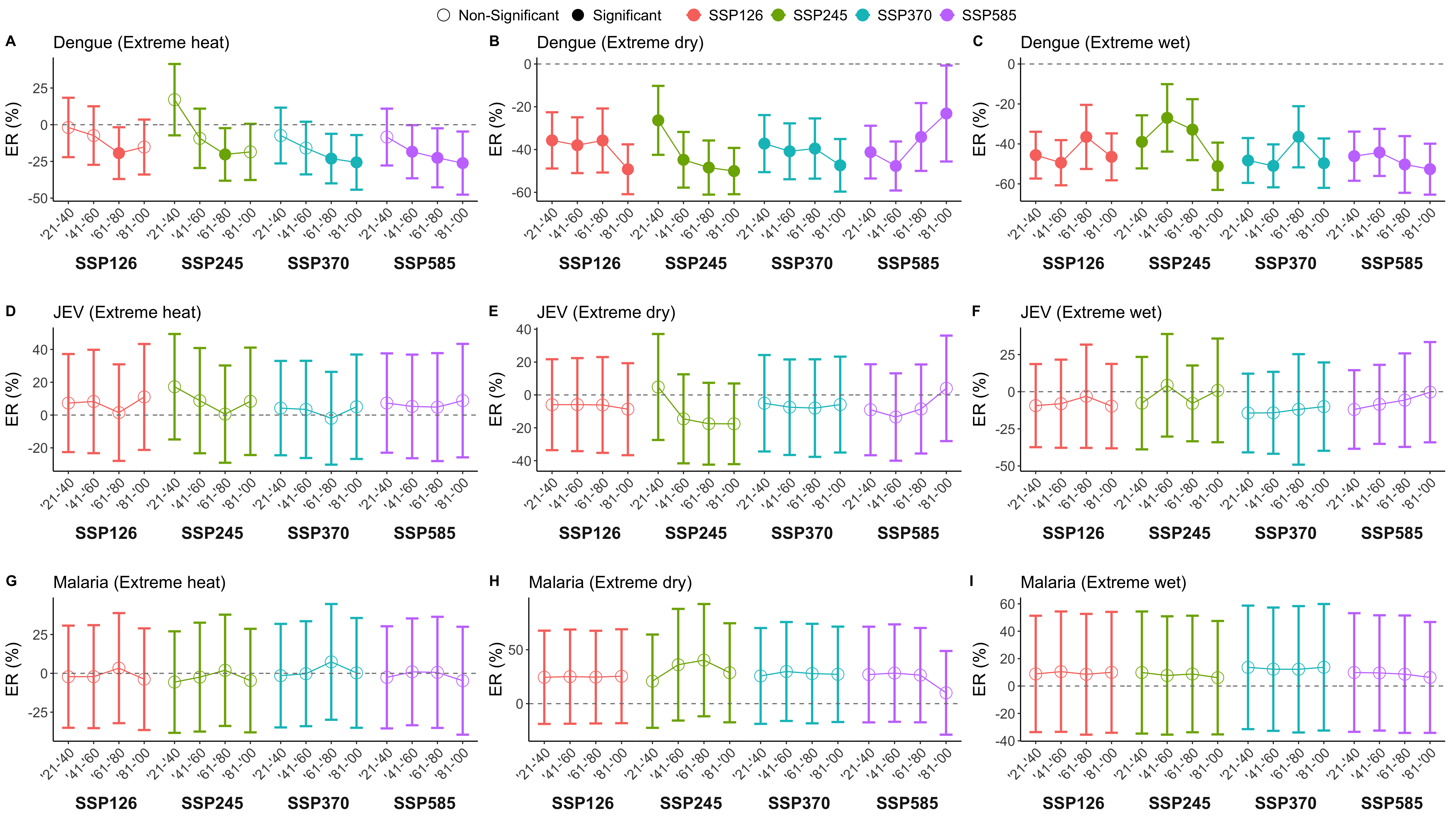

Supplement: S47 Fig — Disease-specific generalized additive models (GAM) were trained on historical data from 2003-2019 and used to project future case counts of the respective disease across 4 time periods (2021–2040, 2041–2060, 2061–2080, 2081–2100) and 4 climate change scenarios (SSP126, SSP245, SSP370, SSP585) during periods of extreme weather. Projections were made based on climate data from the CMCC-ESM2 general circulation model. National-level excess risk was calculated using the mean disease case counts across the historical period and the projected case counts at a respective time period and climate change scenario. Excess risk was stratified across each type of extreme weather, which represents the percentage change in disease cases compared to historical levels during periods of extreme heat, extreme dry weather and extreme wet weather alone. The results obtained using the CMCC-ESM2 general circulation model were generally similar to the main results obtained using the MIROC6 general circulation model. (PNG) [file pntd.0013896.s053.png]

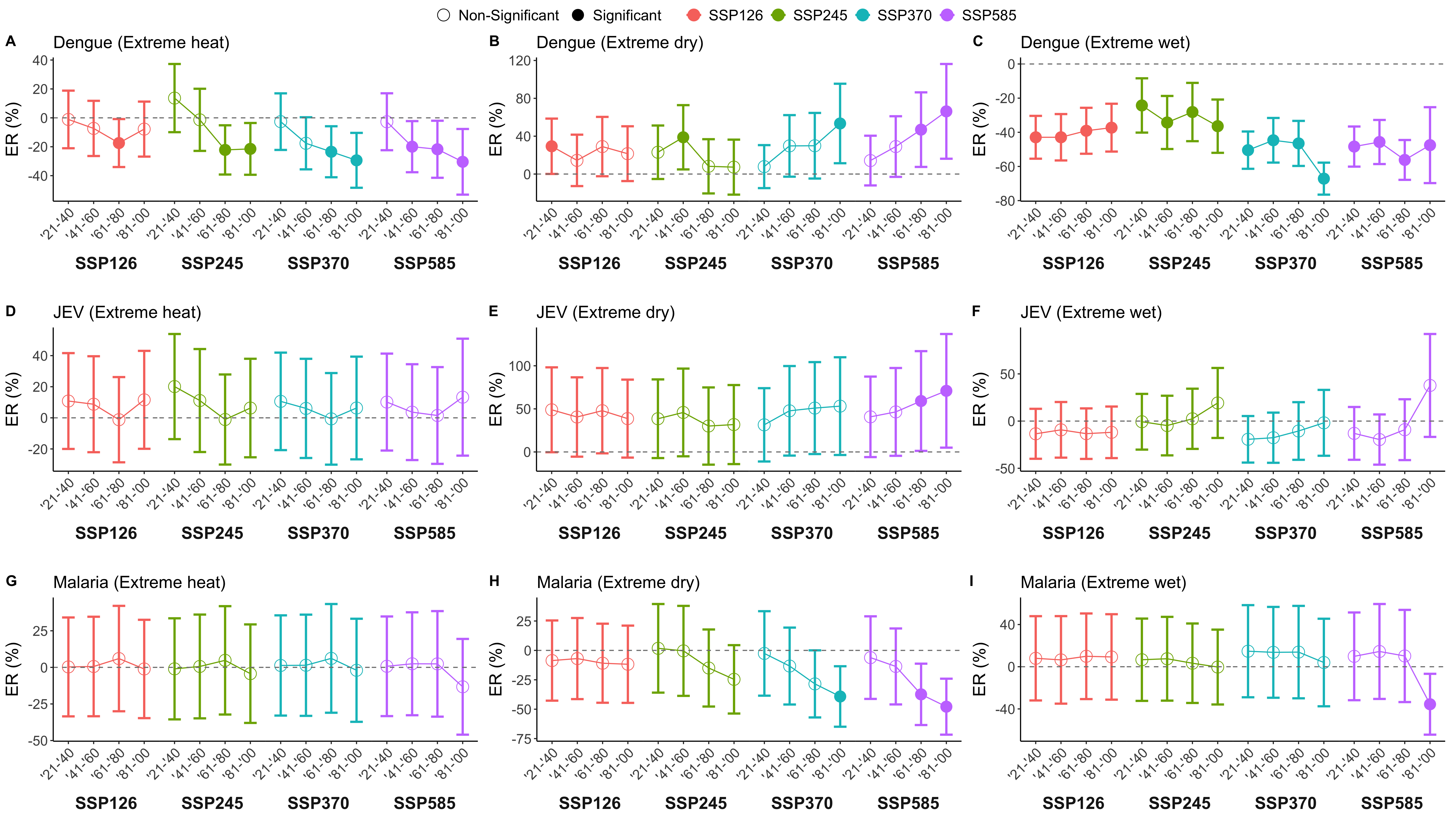

Supplement: S48 Fig — Disease-specific generalized additive models (GAM) were trained on historical data from 2003-2019 and used to project future case counts of the respective disease across 4 time periods (2021–2040, 2041–2060, 2061–2080, 2081–2100) and 4 climate change scenarios (SSP126, SSP245, SSP370, SSP585) during periods of extreme weather. Projections were made based on climate data from the IPSL-CM6A-LR general circulation model. National-level excess risk was calculated using the mean disease case counts across the historical period and the projected case counts at a respective time period and climate change scenario. Excess risk was stratified across each type of extreme weather, which represents the percentage change in disease cases compared to historical levels during periods of extreme heat, extreme dry weather and extreme wet weather alone. The results obtained using the CMCC-ESM2 general circulation model were slightly different from the main results obtained using the MIROC6 general circulation model. Dengue risk is expected to increase during periods of extreme dry weather in all climate change scenarios, which varies from the main model where dengue risk is expected to decrease during periods of extreme dry weather. (PNG) [file pntd.0013896.s054.png]

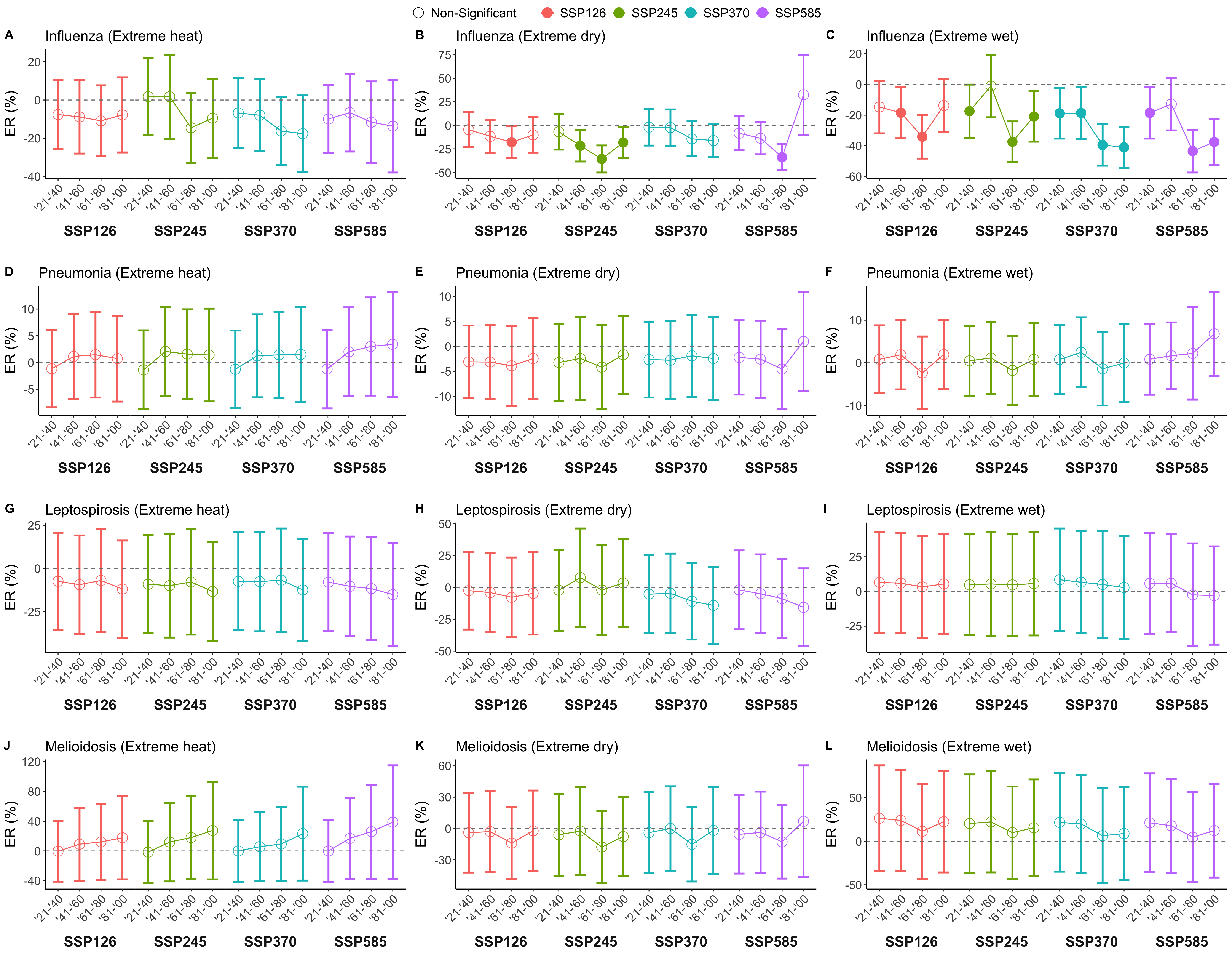

Supplement: S49 Fig — Disease-specific generalized additive models (GAM) were trained on historical data from 2003-2019 and used to project future case counts of the respective disease across 4 time periods (2021–2040, 2041–2060, 2061–2080, 2081–2100) and 4 climate change scenarios (SSP126, SSP245, SSP370, SSP585) during periods of extreme weather. Projections were made based on climate data from the CMCC-ESM2 general circulation model. National-level excess risk was calculated using the mean disease case counts across the historical period and the projected case counts at a respective time period and climate change scenario. Excess risk was stratified across each type of extreme weather, which represents the percentage change in disease cases compared to historical levels during periods of extreme heat, extreme dry weather and extreme wet weather alone. The results obtained using the CMCC-ESM2 general circulation model were generally similar to the main results obtained using the MIROC6 general circulation model. (PNG) [file pntd.0013896.s055.png]

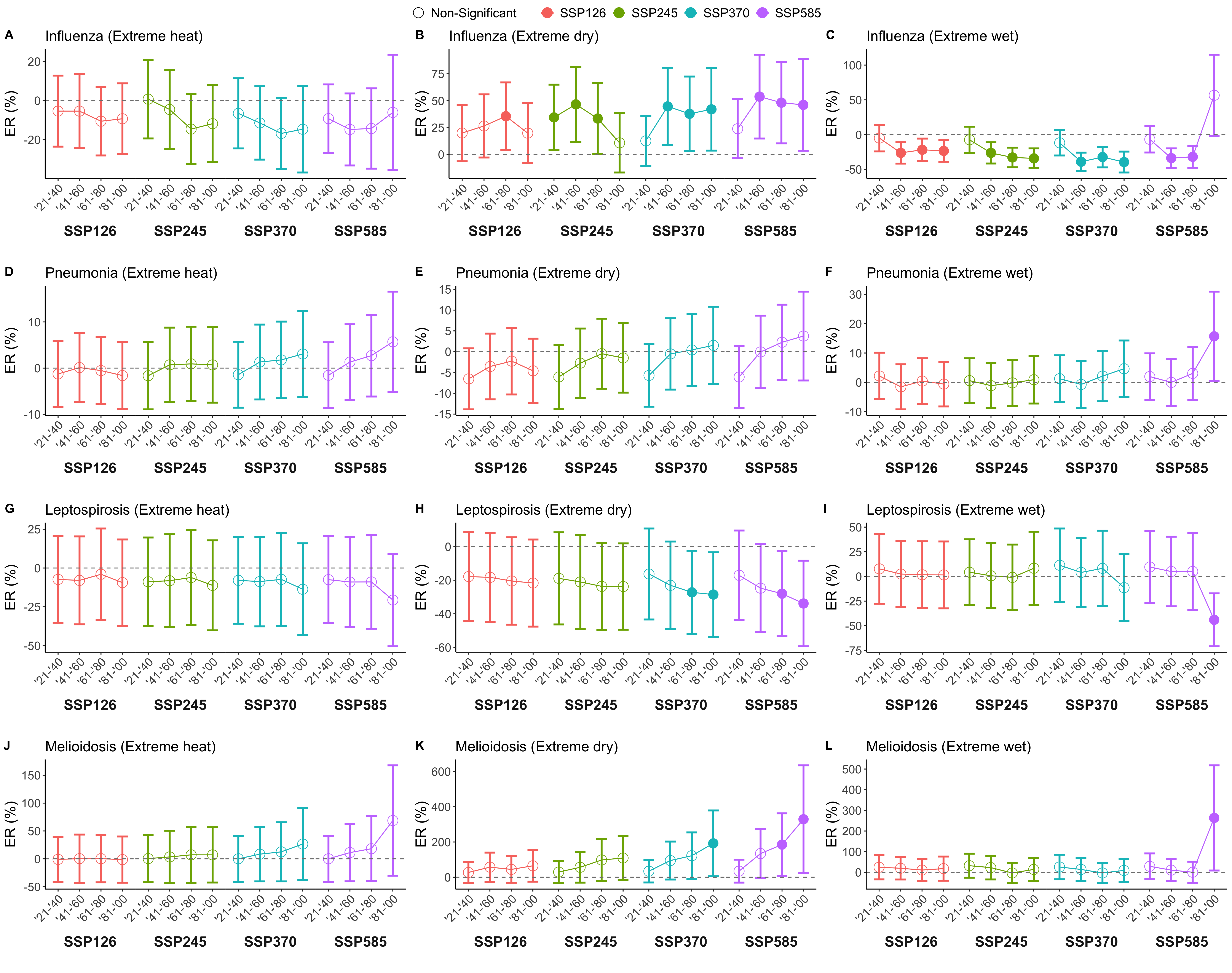

Supplement: S50 Fig — Disease-specific generalized additive models (GAM) were trained on historical data from 2003-2019 and used to project future case counts of the respective disease across 4 time periods (2021–2040, 2041–2060, 2061–2080, 2081–2100) and 4 climate change scenarios (SSP126, SSP245, SSP370, SSP585) during periods of extreme weather. Projections were made based on climate data from the IPSL-CM6A-LR general circulation model. National-level excess risk was calculated using the mean disease case counts across the historical period and the projected case counts at a respective time period and climate change scenario. Excess risk was stratified across each type of extreme weather, which represents the percentage change in disease cases compared to historical levels during periods of extreme heat, extreme dry weather and extreme wet weather alone. The results obtained using the CMCC-ESM2 general circulation model were largely similar to the main results obtained using the MIROC6 general circulation model. The expected increase in influenza during periods of extreme wet and dry weather are much higher than projected in the main model. (PNG) [file pntd.0013896.s056.png]

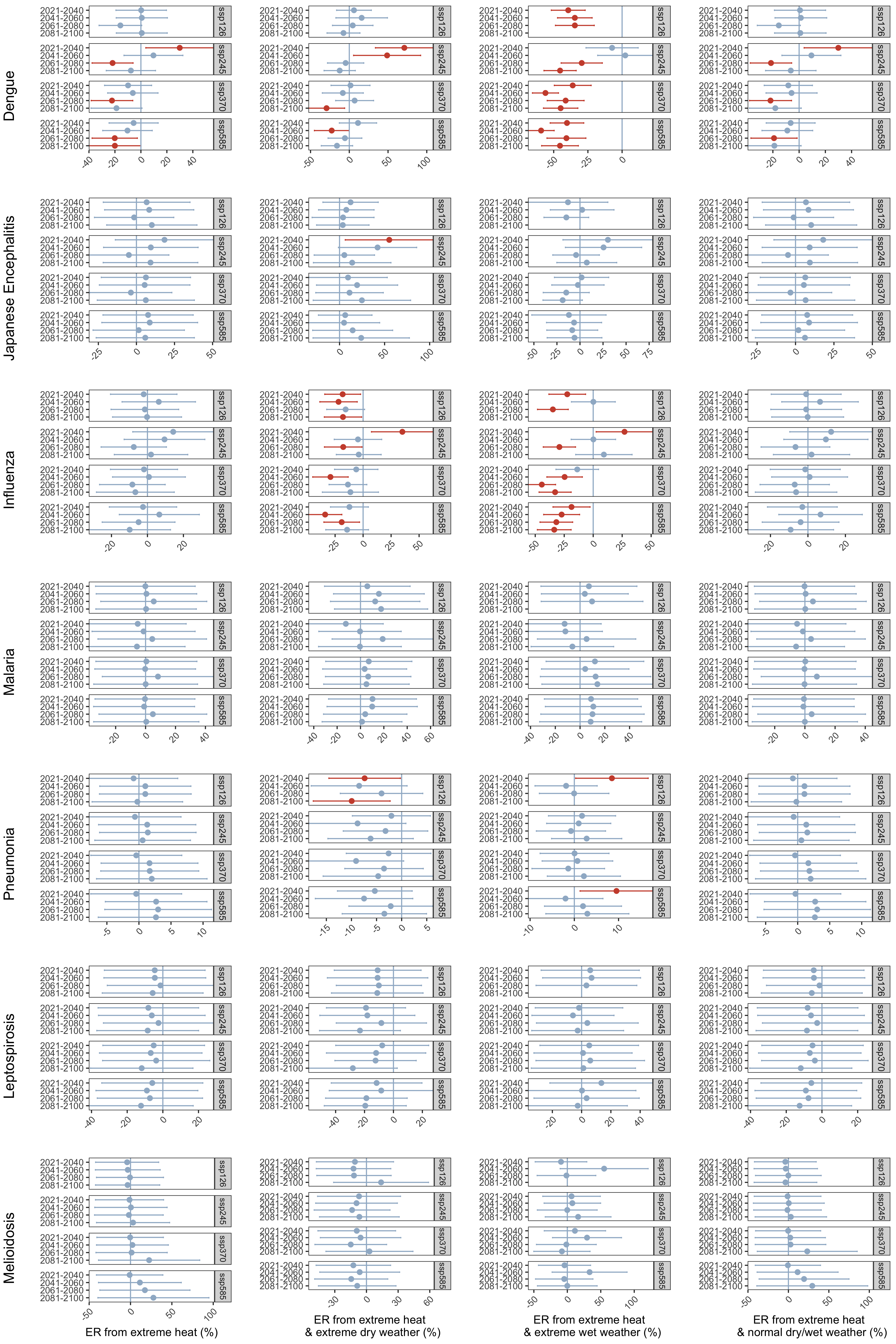

Supplement: S51 Fig — Disease-specific generalized additive models (GAM) were trained on historical data from 2003-2019 and used to project future case counts of the respective disease across 4 time periods (2021–2040, 2041–2060, 2061–2080, 2081–2100) and 4 climate change scenarios (SSP126, SSP245, SSP370, SSP585) during periods of extreme weather. National-level excess risk was calculated using the mean disease case counts across the historical period and the projected case counts at a respective time period and climate change scenario. Excess risk represents the percentage change in disease cases compared to historical levels. We estimated the excess risk attributable to extreme heat, controlling for the presence or absence of concurrent extreme wet weather or extreme dry weather. Excess risks attributable to extreme heat resembled the excess risks attributable to extreme heat with normal dry/wet weather. This indicates that the excess risks attributable to extreme heat were not confounded by the effects of extreme dry/wet weather. (PNG) [file pntd.0013896.s057.png]

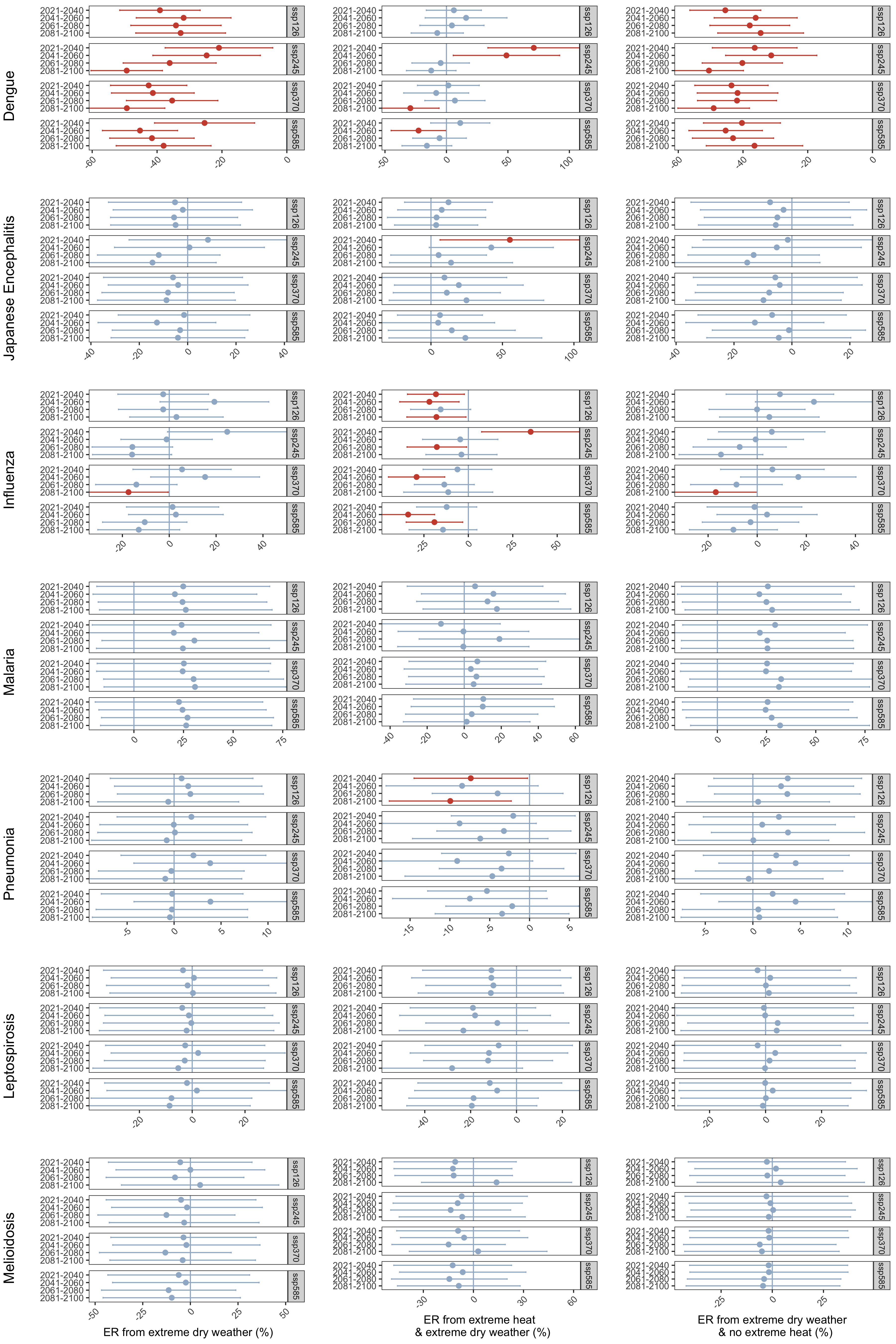

Supplement: S52 Fig — Disease-specific generalized additive models (GAM) were trained on historical data from 2003-2019 and used to project future case counts of the respective disease across 4 time periods (2021–2040, 2041–2060, 2061–2080, 2081–2100) and 4 climate change scenarios (SSP126, SSP245, SSP370, SSP585) during periods of extreme weather. National-level excess risk was calculated using the mean disease case counts across the historical period and the projected case counts at a respective time period and climate change scenario. Excess risk represents the percentage change in disease cases compared to historical levels. We estimated the excess risk attributable to extreme dry weather, controlling for the presence or absence of concurrent extreme heat. Excess risks attributable to extreme dry weather resembled the excess risks attributable to extreme dry weather with no extreme heat. This indicates that the excess risks attributable to extreme dry weather were not confounded by the effects of extreme heat. (PNG) [file pntd.0013896.s058.png]

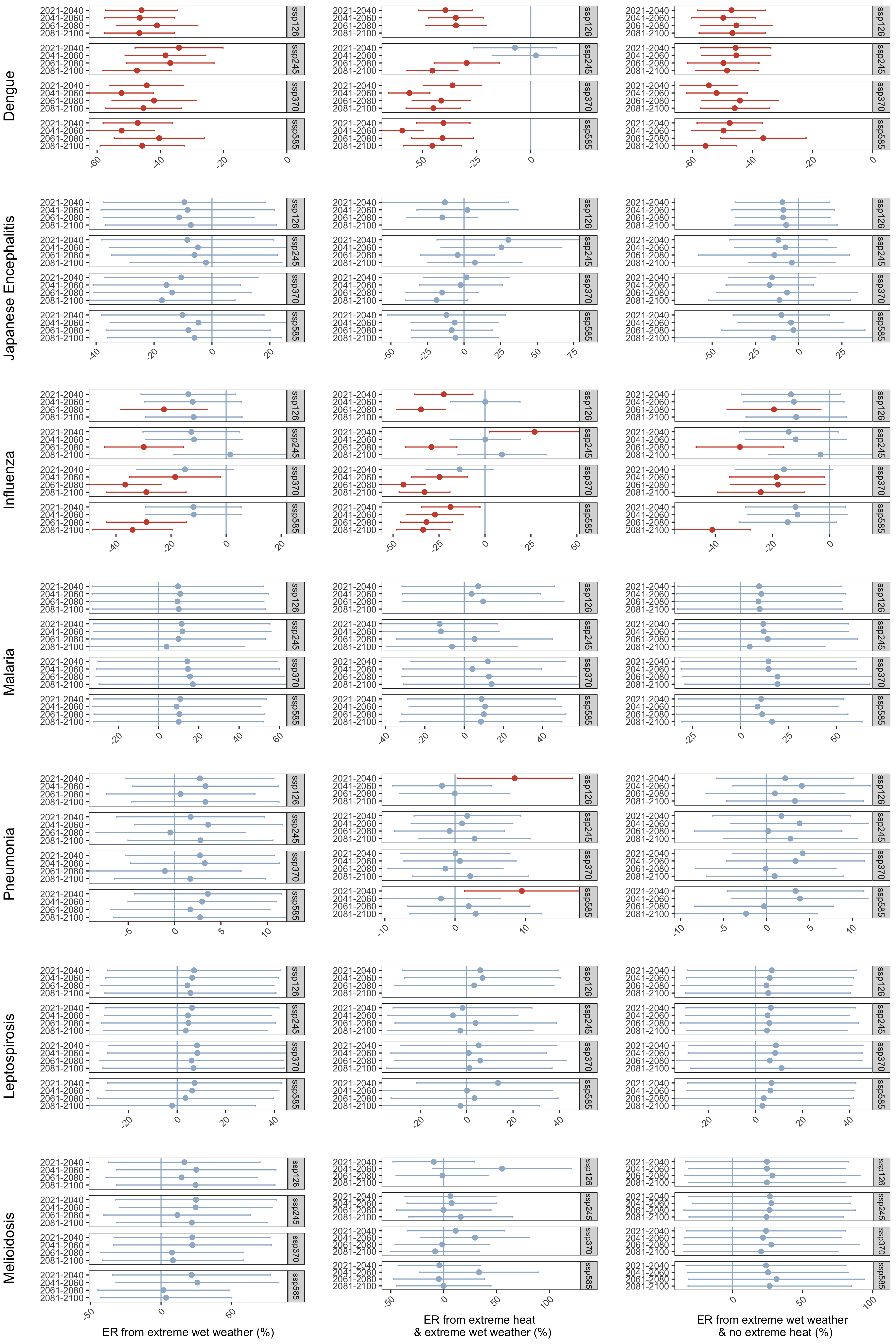

Supplement: S53 Fig — Disease-specific generalized additive models (GAM) were trained on historical data from 2003-2019 and used to project future case counts of the respective disease across 4 time periods (2021–2040, 2041–2060, 2061–2080, 2081–2100) and 4 climate change scenarios (SSP126, SSP245, SSP370, SSP585) during periods of extreme weather. National-level excess risk was calculated using the mean disease case counts across the historical period and the projected case counts at a respective time period and climate change scenario. Excess risk represents the percentage change in disease cases compared to historical levels. We estimated the excess risk attributable to extreme wet weather, controlling for the presence or absence of concurrent extreme heat. The excess risk attributable to extreme wet weather paralleled the excess risks attributable to a combination of extreme wet weather and no extreme heat. This suggests that the excess risks attributable to extreme wet weather were similarly not confounded by the effects of extreme heat. (PNG) [file pntd.0013896.s059.png]
