# Supplementary material for: Projecting long-term excess risks of major infectious diseases associated with future extreme weather events in Thailand
Source: PLoS Negl Trop Dis. 2026 Jan 5;20(1):e0013896. doi: 10.1371/journal.pntd.0013896 (PMC12782439; doi:10.1371/journal.pntd.0013896)
Supplement: Attachment — Submitted filename: 2025 PloS NTD_projections.docx [file pntd.0013896.s060.docx]

In the Research Article, authors first use GAMs to describe the historical relationships between the number of extreme heat days in a month and extreme dry and extreme wet conditions (as measured by SPI) and a number of disease outcomes in Thailand. These diseases include dengue, JEV, influenza, malaria, pneumonia, leptospirosis, and meliodosis. They then apply the exposure-response relationships to projected heat and SPI metrics to estimate the additional number of cases caused or averted by extreme weather at mid- and end-of-century. The paper is ambitious, and the authors should be commended on how much work went into this paper.

Methodically, I believe the work is sound, although I did some questions on the historical results, described below. After reading the manuscript, I did have some comments and areas of confusion, as well as some potential suggestions to improve digestibility of the manuscript for readers.

**General comment:**

I wondered why the authors chose to model days of extreme heat as the exposure rather than merely temperature. The authors make some claim in the introduction that the projected impact of extreme weather events on the selected diseases has not been quantified, but at the same time indicate that this has not been done for temperature either (except in the case of dengue). Is there some additional motivation as to why extreme heat is the right place to start?

My other major comments, which are included below, included confusion over how lags were treated in providing historical results and making projections as well as some seeming mismatch between IRR’s, PAF’s, and directions stated in results.

**Introduction**

- Line 72 discusses previous work finding dengue increases with temperature. Do studies find dengue decreasing with extreme high temperatures; that would set the reader up to see that results align with previous findings
- Again, I felt there should be a better rationale for why the authors did not start with temperature as the exposure

**Methods**

- The discussion around which GCMs were used seemed a bit wordy. I suggest to condense to just say that MIROC6 was used, and then list the others used in sensitivity analysis
- Line 141-145 about the projected population was confusing and I think not needed given that ultimately the authors decide not to report those results. I would just remove projected population mentions from the methods and results and list is as a justifiable limitation in the discussion.
- Line 168-172: I was confused how wet days were defined, and the statement regarding interpolation. Perhaps changes to sentence structure could help
- Table 1: is there a source to cite?
- Line 181: in statistical model, suggest o state what the covariate are
- Equation 1:
  - In line 197, suggest to state that time t is month t
  - Lines 199-200, the letteres don’t seem to align with the equation. For instance, should it be p lags or k lags?q lags or j lags?
  - Line 201, what about f3 – is that not a spline?
- I appreciated the transparency of the equations, but for succinctness, I did not feel all were needed (e.g., equation 8)
- The chosen lags were not clear. Table S2 says that “The number of variable lags selected for models of each disease are bolded and underlined” but I did not see this. It was also not clear how the authors accounted for lags in projections and what lags were represented in the plots in Figure 2.

**Results**

- In Figure 1A, it appears that future projections do not include extreme monthly SPI values. From my understanding, precipitation in many places in the future is expected to become more variable, while potentially not shifting as much on average. Is the projected SPI reflecting an averaging that is cancelling out extreme values. If there are no extreme SPI values projected in the future, how are the projections made?
- Line 358 says, “IRR can be interpreted as ….compared to the baseline”. Can the authors clarify what baseline is here? Presumably it is 0 for SPI, but for heat days, it is 0 or is it one fewer extreme heat days?
- Figure 2: I was confused why the PAF sometimes had unexpected directions for the extreme heat days. For instance, the dengue IRR plot implies lower dengue about 20 heat days per month, whereas the PAF implies a positive percent of dengue cases are attributable to than 20 heat days per month.
- Figure 2: It was unclear to me the reference for the SPI plots. For instance, I expected the reference to be at 0, but the plots did not cross IRR=1 at SPI = 1. Similarly, I presumed the IRR was 1 for heat days = 0, but it would be nice to have this confirmed.
- Paragraph beginning line 370: “In general, extreme heat…”. I suggest to keep the results about heat together and then go into the results about dry or wet conditions. As is, we skip from heat to wet/dry and then back to heat, and then back to wet/dry. Additionally, would it be more accurate to change “and” to “or” in the statement, “increased dry and wet weather increased disease risk with the exception of dengue.”
- Figure 3 was extremely busy, and had overlapping boxes. While I commend the authors for trying to fit so much on a plot, the style of Figure 4 was much cleaner, while conveying important information, like the confidence intervals. I suggest to remove some of the text on the graph and potentially draw more inspiration from Figure 4 style plots.
- Many places in results Figure 1 was referenced when I believe that Figure 2 was meant to be referenced.
- There were many places where I felt the stated direction of the associations did not match the results. These included:
  - Line 418: “Having more than 19 heat days in a month was associated with **an increase** in dengue risk” – the IRR plots seem to imply less (although the PAF does imply more, which is confusing)
  - Line 437: “We expect JEV risk to **increase** during period of extreme dry and extreme wet weather”
  - Line 447: “During periods of extreme dry weather, influenza risk is expected to **decrease…**this relationship is seen historically, where extreme dry weather has a **negative association** with influenza risk”
  - Line 464: “The risk of meliodosis is projected to **increase** on extreme heat days”
- Panels A in Figures 6 and 7 were also very busy. In general, I found the details of the sub-national analysis to be overwhelming and could perhaps be condensed to a few broad trends, with the supplement showing details.
- Model evaluation – I felt model evaluation could be condensed and moved up to be included when talking about the historical patterns. Table 2 could probably be moved to a supplement, while the paragraph about projected population could also be condensed and discussed in a limitation (e.g., we considered using projected populations but did not for __ reasons”
- The results from the sub-scenarios were also somewhat overwhelming to digest.
