# Supplementary material for: Projecting long-term excess risks of major infectious diseases associated with future extreme weather events in Thailand
Source: PLoS Negl Trop Dis. 2026 Jan 5;20(1):e0013896. doi: 10.1371/journal.pntd.0013896 (PMC12782439; doi:10.1371/journal.pntd.0013896)
Supplement: Attachment — Submitted filename: Response to Reviewers.docx [file pntd.0013896.s061.docx]

PNTD-D-25-00718

Response to reviewers

Dear Dr. Shaden Kamhawi, Dr Paul Brindley and Dr Peter J. Hotez,

Thank you for the opportunity to submit a revised draft of the manuscript entitled “Projecting long-term excess risks of major infectious diseases associated with future extreme weather events in Thailand” to *PLOS Neglected Tropical Diseases*. We appreciate the time and effort that you and the reviewers have dedicated to providing your valuable feedback on my manuscript. We have been able to incorporate changes to reflect the suggestions provided by the reviewers and have tracked the changes within the manuscript. Here is a point-by-point response to the reviewers’ comments and concerns.

Comments from Reviewer 1

General comments

1. **Comment:** *I wondered why the authors chose to model days of extreme heat as the exposure rather than merely temperature. The authors make some claim in the introduction that the projected impact of extreme weather events on the selected diseases has not been quantified, but at the same time indicate that this has not been done for temperature either (except in the case of dengue). Is there some additional motivation as to why extreme heat is the right place to start?*

**Response:** We thank the reviewer for their question. We agree that both temperature and extreme heat are important exposures influencing disease transmission. Several studies have been published exploring the projected impact of mean temperature and precipitation on infectious diseases, including dengue (<10.17605/OSF.IO/85XWQ>), influenza (<https://www.nature.com/articles/s41612-025-00968-3>), leptospirosis (10.1088/1755-1315/1089/1/012074) and malaria ([10.1029/2025GH001541](https://doi.org/10.1029/2025GH001541)). In contrast, few studies have specifically examined extreme heat, even at the association level despite its growing public health relevance under climate change. Thus, focusing on extreme heat allows us to address an underexplored but increasingly important aspect of temperature-disease relationships. We have updated the Introduction to justify our focus on extreme heat rather than just temperature (line 82-91):

“Most studies have focused on the effects of mean temperature and rainfall on future disease burden. Recent work has projected the burden of influenza^10^, leptospirosis^11^, malaria^12^ and dengue^13^ based on temperature and precipitation projections under different climate change scenarios. Colón-González et al. projected the future burden of dengue in Southeast Asia based on climate, population, human mobility and gross domestic product projections – expecting it to peak in this century before declining to lower levels. Heterogenous impacts were anticipated across the region – with long term average decreases in dengue burden in Thailand and Cambodia^13^. However, although extreme weather events are expected to increase in occurrence, no study has attempted to quantify the impact of extreme weather events on climate-sensitive infectious diseases.”

Introduction

2. **Comment:** *Line 72 discusses previous work finding dengue increases with temperature. Do studies find dengue decreasing with extreme high temperatures; that would set the reader up to see that results align with previous findings*

**Response:** We thank the reviewer for their question. We have revised the Introduction to include past studies which found that dengue risk was inversely associated with extreme high temperatures (line 72-73):

“A systematic review and meta-analysis including 106 studies demonstrated that 1 °C increase in high temperature was associated with a 13% increase in dengue risk^6^. Another study found that beyond 31 °C, a 1 °C increase in maximum temperature was associated with 13.1% decrease in cumulative dengue risk over 6 weeks^7^.”

3. **Comment:** *Again, I felt there should be a better rationale for why the authors did not start with temperature as the exposure*

**Response:** We thank the reviewer for their comment. We agree that both temperature and extreme heat are important exposures influencing disease transmission. Several studies have been published exploring the projected impact of mean temperature and precipitation on infectious diseases, including dengue (<10.17605/OSF.IO/85XWQ>), influenza (<https://www.nature.com/articles/s41612-025-00968-3>), leptospirosis (10.1088/1755-1315/1089/1/012074) and malaria ([10.1029/2025GH001541](https://doi.org/10.1029/2025GH001541)). In contrast, few studies have specifically examined extreme heat, even at the association level despite its growing public health relevance under climate change. Thus, focusing on extreme heat allows us to address an underexplored but increasingly important aspect of temperature-disease relationships. We have updated the Introduction to justify our focus on extreme heat rather than just temperature (line 82-91):

“Most studies have focused on the effects of mean temperature and rainfall on future disease burden. Recent work has projected the burden of influenza^10^, leptospirosis^911^, malaria^12^ and dengue^13^ based on temperature and precipitation projections under different climate change scenarios. Colón-González et al. projected the future burden of dengue in Southeast Asia based on climate, population, human mobility and gross domestic product projections – expecting it to peak in this century before declining to lower levels. Heterogenous impacts were anticipated across the region – with long term average decreases in dengue burden in Thailand and Cambodia^13^. However, although extreme weather events are expected to increase in occurrence, no study has attempted to quantify the impact of extreme weather events on climate-sensitive infectious diseases.”

Methods

4. **Comment:** *The discussion around which GCMs were used seemed a bit wordy. I suggest to condense to just say that MIROC6 was used, and then list the others used in sensitivity analysis*

**Response:** We thank the reviewer for their suggestion. The discussion around GCMs highlights that we have considered multiple GCMs and justifies the selection of the 3 GCMs used in the study. We believe these details would help readers understand the robustness of our results and the rationale behind our model choices. Hence, we have chosen to retain the original discussion.

5. **Comment:** *Line 141-145 about the projected population was confusing and I think not needed given that ultimately the authors decide not to report those results. I would just remove projected population mentions from the methods and results and list is as a justifiable limitation in the discussion.*

**Response:** We thank the reviewer for this comment. While we understand that the discussion of projected population may have seemed unnecessary, it represents an important part of our sensitivity analysis and was also requested by another reviewer. We have therefore retained these descriptions but revised the text (Lines 145-147) to clarify why and how the additional population data set was used:

**“**We obtained a second historical population dataset from WorldPop for each province from 2003 to 2019, which was the data used to train the CMIP6 population projection data^24^. This dataset was used exclusively to train the models in the sensitivity analysis that incorporated future population changes. For the main analysis, we used government census-based population data, which are more reliable for historical estimates but do not extend to future projections.”

6. **Comment:** *Line 168-172: I was confused how wet days were defined, and the statement regarding interpolation. Perhaps changes to sentence structure could help*

**Response:** We thank the reviewer for their comment. We have restructured the sentence to make the statement clearer (line 181-188):

“MODAWEC, a daily weather generator, was used to disaggregate daily precipitation, daily maximum and daily minimum temperature from the respective monthly data^32^. The monthly number of wet days was also required as an input by MODAWEC, where a wet day is defined as a day with more than 1 mm of precipitation, which is consistent with standard definitions and climate studies. The future number of wet days were linearly interpolated based on historical daily precipitation data and number of wet days.”

7. **Comment:** *Table 1: is there a source to cite?*

**Response:** We thank the reviewer for their question. We have included the source for the table, which is the article “The Shared Socioeconomic Pathways and their energy, land use, and greenhouse gas emissions implications: An overview” (Riahi et al., 2017).

8. **Comment**: *Line 181: in statistical model, suggest to state what the covariate are*

**Response:** We thank the reviewer for their suggestion. We have revised the Methods to state the covariates (line 197-200):

“Generalised additive models (GAMs) with a negative binomial distribution were used to model the associations between disease case counts and the covariates. The covariates include lagged monthly SPI, lagged number of monthly extreme heat days, population density and contemporaneous relative humidity.”

9. **Comment:** *Equation 1:*
   a. *In line 197, suggest to state that time t is month t*
   b. *Lines 199-200, the letters don’t seem to align with the equation. For instance, should it be p lags or k lags? q lags or j lags?*
   c. *Line 201, what about f3 – is that not a spline?*

**Response:** We thank the reviewer for their comments. For comment b, we would like to clarify that there are k = {1, 2, …, p} lags for SPI and j = {1, 2, …, q} lags for number of extreme heat days. We have modified Equation 1 according to the reviewer’s comments (line 212-220):

“$\log\left( y_{t, d,l} \right) =\alpha_{d}+\log\left( P_{a\left[ t \right],l} \right) + +\sum_{k=1}^{p} f_{1,k,d}\left( x_{1,t-k} \right)+\sum_{j=1}^{q} f_{2,j,d}\left( x_{2,t-j} \right)+ f_{3,d}\left( m\left[ t \right] \right)+f_{4,d}\left( x_{3,a\left[ t \right], l} \right)+ f_{5,d}(z_{t,l})+ \gamma_{l}$ (1)

where $y_{t,d,l}$ is the case count of disease $d$ at monthly time $t$ in province $l$, $\alpha$is the intercept, $\log\left( P_{a\left[ t \right]} \right)$ is the logarithm of the province population at year $a\left[ t \right]$ which is used as an offset for the at-risk population. $x_{1,t-k}$ is the SPI up to $k=\{1,2,..,p\}$lags, $x_{2,t-j}$ is the number of extreme heat days in a month up to $j=\{1, 2,...,q\}$lags, $x_{3,a\left[ t \right], l}$ is the population density at year $a\left[ t \right]$ in province $l$, $z_{t,l}$ is the contemporaneous relative humidity in province $z_{t,l}$, and $f_{1,k,d},$ $f_{2,j,d}$, $f_{3,d}$, $f_{4,d}$ and $f_{5,d}$ are the thin-plate splines for disease $d$.”

10. **Comment:** *I appreciated the transparency of the equations, but for succinctness, I did not feel all were needed (e.g., equation 8)*

**Response:** We thank the reviewer for their suggestion. We have compressed equation 8 into plain language for succinctness (line 355-358):

“Annual national-level ER is calculated by modifying (6) such that incidences are aggregated over all provinces. This represents the percentage change in projected nationwide incidence under extreme weather conditions compared to the expected nationwide incidence under historical average weather.”

11. **Comment:** *The chosen lags were not clear. Table S2 says that “The number of variable lags selected for models of each disease are bolded and underlined” but I did not see this. It was also not clear how the authors accounted for lags in projections and what lags were represented in the plots in Figure 2.*

**Response:** We thank the reviewer for their comment. We would like to clarify that the chosen lags were bolded and underlined in the table. To make the table clearer, we have also highlighted the chosen lags in Table S2.

In projections, lagged extreme heat days and SPI were taken from the corresponding months in the preceding periods. However, as climate projections only provide multi-year monthly averages rather than the continuous monthly time series, lagged covariates are treated as cyclical. For example, for disease projections in February, 3 months of lagged SPI would be taken from SPI in January, December and November. We have revised the Methods to explain how we accounted for lags in projections (line 315-330):

“

$$\hat{y}_{proj,d,m, t,l,s}=E\left[ \hat{y}_{d,m,t,l,s} | x_{i}=x_{i,m,t,l,s}^{*},\left\{ x_{1, m-k,t,l,s}^{*} | 1\leq k\leq p \right\},\left\{ x_{2,m-q,t,l,s}^{*} | 1\leq j\leq q \right\},x_{-i}=\bar{x_{-i,m,l}} \right]$$

(4)

where $\hat{y}$ is the estimated incidence rate of the disease $d$ in month $m$ of future time period $t$ and province $l$ under climate change scenario $s$. $x_{i}$ include the covariates SPI, number of extreme heat days and relative humidity, and $x_{i,s,m}^{*}$is the covariate of interest $i$ in month $m$ of future time period $t$ under climate change scenario $s$ in province $l$. $x_{1, m-k,t,l,s}^{*}$ is the lagged monthly extreme heat days of up to $p$ lags, while $x_{2, m-q,t,l,s}^{*}$ is the lagged monthly extreme heat days of up to $q$ lags. The number of lags are shown in Supplementary Table 2. As climate projections only provide multi-year monthly averages rather than the continuous monthly time series, lagged covariates are treated as cyclical. For example, for disease projections in February, 3 months of lagged SPI would be taken from SPI in January, December and November.”

In figure 2, the incidence rate ratios (IRRs) and population attributable fractions (PAFs) were calculated based on a change in contemporaneous SPI or extreme heat days. We have revised the Results to clarify this (line 403-407):

“IRR can be interpreted as the ratio change in incidence rates given exposure to extreme weather in the same month compared to the baseline (0 heat days and 0 SPI respectively), while PAF can be interpreted as the proportion of cases in a population that could be avoided in the absence of anomalous SPI and extreme heat in the same month.”

Results

12. **Comment:** *In Figure 1A, it appears that future projections do not include extreme monthly SPI values. From my understanding, precipitation in many places in the future is expected to become more variable, while potentially not shifting as much on average. Is the projected SPI reflecting an averaging that is cancelling out extreme values. If there are no extreme SPI values projected in the future, how are the projections made?*

**Response:** We thank the reviewer for their question. Figure 1A does not show extreme monthly SPI as it is aggregated nationwide to provide a summary statistic. At the province level, extreme SPI values are still observed which allows us to make projections. We have revised the Results to explain this observation (line 389-391):

“From 2021 to 2100, extreme SPI values are only observed at the province level (Supplementary Figure S1).”

Furthermore, future SPI values for each month are averaged across 20 years, which may cancel out extreme values. This may be why there are no extreme nationwide SPI values. We have included this possibility as a limitation in the Discussion (line 757-763):

“Meanwhile, extreme SPI values appeared less frequently in the GCM projections compared to the historical period. This is likely because each monthly value represents a 20-year average, which smooths out year-to-year extremes. Hence, our results might underestimate the impact of extreme dry or wet weather on disease risk. Future research should focus on the development of GCM outputs that provide continuous monthly data for future periods, which would allow for a more accurate representation of interannual climate variability and extreme events.”

13. **Comment:** *Line 358 says, “IRR can be interpreted as ….compared to the baseline”. Can the authors clarify what baseline is here? Presumably it is 0 for SPI, but for heat days, it is 0 or is it one fewer extreme heat days?*

**Response:** We thank the reviewer for their question. The baseline for SPI is 0 and for heat days is 0 days. We have revised the Results to reflect this (line 403-407):

“IRR can be interpreted as the ratio change in incidence rates given exposure to extreme weather in the same month compared to the baseline (0 heat days and 0 SPI respectively), while PAF can be interpreted as the proportion of cases in a population that could be avoided in the absence of anomalous SPI and extreme heat in the same month.”

14. **Comment:** *Figure 2: I was confused why the PAF sometimes had unexpected directions for the extreme heat days. For instance, the dengue IRR plot implies lower dengue about 20 heat days per month, whereas the PAF implies a positive percent of dengue cases are attributable to than 20 heat days per month.*

**Response:** We thank the reviewer for their comment. We apologise as there was a mistake in the calculation of IRRs. We have corrected the plot and all downstream results accordingly.

Figure 2: National level incidence rate ratio (IRR) and population attributable fraction (PAF) over days of extreme heat and standardised precipitation index (SPI) for dengue, JEV, influenza, malaria, pneumonia, leptospirosis and melioidosis. Bars represent 95% confidence intervals and red bars represent statistically significant points.

15. **Comment:** *Figure 2: It was unclear to me the reference for the SPI plots. For instance, I expected the reference to be at 0, but the plots did not cross IRR=1 at SPI = 1. Similarly, I presumed the IRR was 1 for heat days = 0, but it would be nice to have this confirmed.*

**Response:** We thank the reviewer for their comment. We apologise as there was a mistake in the calculation of IRRs. IRR and PAF now crosses 1 when SPI=0, which is the baseline. For extreme heat, we did not include 0 extreme heat days in the axis, hence IRR and PAF do not cross 1. We have corrected the plot and all downstream results accordingly.

Figure 2: National level incidence rate ratio (IRR) and population attributable fraction (PAF) over days of extreme heat and standardised precipitation index (SPI) for dengue, JEV, influenza, malaria, pneumonia, leptospirosis and melioidosis. Bars represent 95% confidence intervals and red bars represent statistically significant points.

16. **Comment:** *Paragraph beginning line 370: “In general, extreme heat…”. I suggest to keep the results about heat together and then go into the results about dry or wet conditions. As is, we skip from heat to wet/dry and then back to heat, and then back to wet/dry. Additionally, would it be more accurate to change “and” to “or” in the statement, “increased dry and wet weather increased disease risk with the exception of dengue.”*

**Response:** We thank the reviewer for their suggestion. We have restructured the paragraph accordingly to improve the readability (line 416-433):

“In dengue, JEV, influenza, pneumonia and melioidosis, the number of extreme heat days in a month was positively associated with disease risk (Fig. 2). On the other hand, increasing days of extreme heat days in a month was negatively associated with malaria and leptospirosis risk (Fig. 2). The pattern was also consistent when the association of disease incidence against extreme weather was broken down on a province-specific basis, particularly in highly populated provinces like Bangkok, Nakhon Ratchasima and Ubon Ratchathani (Supplementary Figure S2–10). Province-level PAFs demonstrated the same trend, with positive values signifying that a proportion of cases in the population can be attributed to extreme heat, extreme dry weather and extreme wet weather respectively (Supplementary Figure S11–17).

Extreme wet weather (i.e. SPI $\geq$1.5) was historically associated with lower incidence of dengue and higher incidence of JEV, pneumonia and melioidosis. At SPI levels of 3, dengue IRR was 0.66 (95% CI: 0.62, 0.69), while JEV IRR was 1.29 (95% CI: 1.19, 1.40). Extreme dry weather (i.e. SPI $\leq$ -1.5) was historically associated with higher incidence of JEV and lower incidence of dengue and pneumonia (Fig. 2). At SPI levels of $-$3, dengue IRR was 0.88 (95% CI: 0.83, 0.92), while JEV IRR was 1.21 (95% CI: 1.12, 1.30). Province-level IRRs and PAFs were concordant with these trends (Supplementary Figure S2-17).”

17. **Comment:** *Figure 3 was extremely busy, and had overlapping boxes. While I commend the authors for trying to fit so much on a plot, the style of Figure 4 was much cleaner, while conveying important information, like the confidence intervals. I suggest to remove some of the text on the graph and potentially draw more inspiration from Figure 4 style plots.*

**Response:** We thank the reviewer for their comment. We understand that the Figure 3 may appear visually complex. However, it aims to give a comprehensive overview of nationwide excess risk estimates for across diseases, SSPs and time periods. Due to the complexity and breadth of our study, simplifying the figure would limit its ability to convey the full scope of our results. Hence, we have chosen to retain the original figure to preserve the completeness of the information presented.

18. **Comment:** *Many places in results Figure 1 was referenced when I believe that Figure 2 was meant to be referenced.*

**Response:** We thank the reviewer for their comment and apologise for the mistake. We have revised the incorrect figure labelling throughout the text.

19. **Comment:** *There were many places where I felt the stated direction of the associations did not match the results. These included:*
    a. *Line 418: “Having more than 19 heat days in a month was associated with* ***an increase*** *in dengue risk” – the IRR plots seem to imply less (although the PAF does imply more, which is confusing)*
    b. *Line 437: “We expect JEV risk to* ***increase*** *during period of extreme dry and extreme wet weather”*
    c. *Line 447: “During periods of extreme dry weather, influenza risk is expected to* ***decrease…****this relationship is seen historically, where extreme dry weather has a* ***negative association*** *with influenza risk”*
    d. *Line 464: “The risk of meliodosis is projected to* ***increase*** *on extreme heat days”*

**Response:** We thank the reviewer for their comment. In statement a, b and d, the misalignment was due to a miscalculation of historical association shown in Figure 2. These statements match the results after the correction. For statement c, we have corrected the description to correspond with the results (line 502-504):

“The greatest expected decrease during periods of extreme wet weather can be seen under SSP370, which decreases by -37.8% (95% CI: -50.8%, -24.8%) from 2061–2080 (Fig. 5C). This relationship is also seen historically, where extreme wet weather has a negative association with influenza risk (Fig. 2).”

20. **Comment:** *Panels A in Figures 6 and 7 were also very busy. In general, I found the details of the sub-national analysis to be overwhelming and could perhaps be condensed to a few broad trends, with the supplement showing details.*

**Response:** We thank the reviewer for this helpful comment. We acknowledge that Panels A in Figures 6 and 7 are visually dense. However, these panels are designed to illustrate the province-level heterogeneity in excess risks, which allows us to examine spatial variation rather than only broader trends. As identifying provinces or regions projected to experience higher future incidence is a key focus of our analysis, we have chosen to retain the original figure.

21. **Comment:** *Model evaluation – I felt model evaluation could be condensed and moved up to be included when talking about the historical patterns. Table 2 could probably be moved to a supplement, while the paragraph about projected population could also be condensed and discussed in a limitation (e.g., we considered using projected populations but did not for __ reasons)”*

**Response:** We thank the reviewer for their suggestions. We chose not to condense to the model evaluation section as it is important for justifying our model choice. We have moved Table 2 to the supplement as suggested to condense the section. Regarding the paragraph on projected population, we retained it to clearly explain why we did not use projected population and because it was requested by another reviewer.

22. **Comment:** *The results from the sub-scenarios were also somewhat overwhelming to digest.*

**Response:** We thank the reviewer for their comment. We have simplified and condensed the results from the sub-scenarios to improve readability (line 635-651):

“We first compared excess risk attributable to extreme heat against sub-scenarios combining extreme heat and extreme dry weather, extreme wet weather or normal conditions. Across diseases, the excess risks attributable to extreme heat were showed different trends from those under the combined sub-scenarios (Supplementary Figure S45). This suggests that excess risks attributable to extreme heat estimated may be influenced by other extreme weather conditions.

Additionally, we compared the excess risk attributable to extreme dry weather with those under sub-scenarios involving extreme dry weather with or without extreme heat respectively. Excess risks attributable to extreme dry weather resembled the excess risks attributable to extreme dry weather with no extreme heat (Supplementary Figure S46). This indicates that the excess risks attributable to extreme dry weather were not confounded by the effects of extreme heat.

Lastly, we compared the excess risk attributable to extreme wet weather with those under sub-scenarios involving extreme wet weather with or without extreme heat respectively. The excess risks were likewise consistent, suggesting that the excess risks attributable to extreme wet weather were similarly not confounded by the effects of extreme heat (Supplementary Figure S47).”

Comments from Reviewer 2

Major comments

1. **Comment**: *Extreme heat threshold: You set the national 90th percentile Tmax (34.9 °C) and tally the number of days in a month that have at least three days of heat. This might put heat in the wrong category in milder northern and highland areas and downplay heat in hotter southern and coastal provinces. Please test percentiles that are particular to a province or climatic zone and let us know how sensitive IRR/ER is to that decision.*

**Response**: We thank the reviewer for their comment. We acknowledge that the temperature variation across Thailand may influence the characterization of extreme heat events. We separated Thailand by its 6 geographical regions and obtained the 90^th^ percentile Tmax for each region. Each region’s 90^th^ percentile Tmax differed by only about 1 °C from the national 90^th^ percentile Tmax which was used in the study.

| Region | 90^th^ percentile Tmax / °C |
| --- | --- |
| Northern | 35.21 |
| Northeastern | 35.52 |
| Central | 36.10 |
| South | 33.51 |
| East | 33.84 |
| West | 35.52 |

Furthermore, using a single national-level threshold ensures consistency and comparability across regions and provinces, enabling clearer interpretation of spatial differences in outcomes.

We have revised the Methods section to justify the use of a national-level threshold across regions (line 156-159):

“A single national threshold, rather than a region-specific threshold, was used to define extreme heat days to ensure comparability across regions. Furthermore, the 90th percentile daily maximum temperature differed by only 1 to 2 °C across geographical regions, supporting the use of a national threshold.”

2. **Comment:** *You utilise SPI-1 (no PET) since PET projections are restricted. Think about utilising SPEI-1 with PET approximations (such Hargreaves or Penman-Monteith based on CMIP6 variables) to see how sensitive it is, or at least prove that SPI-1 and SPEI-1 categorise dry and wet months in the same way for a historical overlap. This will make it easier to draw conclusions about how heat and moisture interact*.

**Response:** We thank the reviewer for their comment. Following the reviewer’s suggestion, we calculated monthly SPEI-1 values and approximated evapotranspiration following the 1985 Hargreaves equation. We compared the SPEI-1 values to the original SPI-1 values. SPEI-1 and SPI-1 classify months identically into extreme wet (>1.5), extreme dry (< -1.5) or normal in 81% of the data. Furthermore, the distribution of wet, dry and normal months classified by SPI-1 and SPEI-1 are largely similar, as shown in the table below.

|  | Number of dry months | Number of normal months | Number of wet months |
| --- | --- | --- | --- |
| SPI-1 | 1088 | 15205 | 1035 |
| SPEI-1 | 1107 | 15162 | 1059 |

Hence, using SPI-1 rather than SPEI-1 would not significantly alter the results of our study. We have revised the Methods to justify the use of SPI (line 164-168):

“To test if SPEI and SPI categorised wet and dry months similarly, potential evapotranspiration was estimated for the historical period by using the Hargreaves equation to calculate SPEI. SPEI-1 and SPI-1 categorised extreme dry, extreme wet and normal months identically in 81% of the data. Hence, the use of SPI rather than SPEI would not have significantly affected our results.”

3. **Comment:** *MODAWEC and linearly interpolated wet-day counts are used to create daily sequences for heatwaves and wet days from monthly averages. This may smooth out persistence and tails, which might change the durations of intense heat and heavy rain periods that affect transmission and exposure paths. Please compare the outputs of the generators to the reanalysis (ERA5) for the years 2003 to 2019 on metrics such the distribution of heatwave durations, the lengths of wet-day spells, and the daily precipitation at the 95th and 99th percentiles at the provincial level. Please quantify any attenuation and change the predictions or uncertainty as needed.*

**Response:** We thank the reviewer for their suggestion. We acknowledge that downscaling monthly temperature data may underestimate daily extremes. To clarify, SPI was estimated using monthly precipitation values and did not rely on downscaled precipitation values. From the MODAWEC output, only daily maximum temperature was used to calculate the number of extreme heat days. We have revised the Methods to express this clearer (line 181-188):

“MODAWEC, a daily weather generator, was used to disaggregate daily precipitation, daily maximum and daily minimum temperature from the respective monthly data^32^. The monthly number of wet days was also required as an input by MODAWEC, where a wet day is defined as a day with more than 1 mm of precipitation, which is consistent with standard definitions and climate studies. The future number of wet days were linearly interpolated based on historical daily precipitation data and number of wet days. Only the daily maximum temperature was used in subsequent analyses to determine the monthly number of extreme heat days.”

Furthermore, we averaged the daily ERA5 maximum temperature, minimum temperature and total precipitation into monthly data, then used MODAWEC to retrieve the daily measurements. From the daily measurements, we compared the number of extreme heat days a month across provinces in the actual data and the data downscaled by MODAWEC. We found that the direction of bias was unclear. MODAWEC overestimated the number of months with less than 8 heat days, but underestimated the number of months with more than 8 heat days. Hence, our projections may not capture the exact distribution of heat extremes observed in reality. We have highlighted this in the Discussion as a limitation (line 750-754):

“A limitation of our study was in the temporal disaggregation of the climate change projections. Future monthly temperature was disaggregated to daily temperature to determine the number of extreme heat days. However, this approach may not accurately capture the true variability and daily extremes within each month. Hence, the projected frequency of extreme heat events may differ slightly from actual conditions.”

Figure A: Number of months experiencing a given number of extreme heat days across all provinces based on actual data and data downscaled with MODAWEC.

4. **Comment:** *Monthly case numbers very definitely show some degree of autocorrelation. The GAM framework has seasonality but not a clear AR term. Please report the remaining ACF/PACF and, if necessary, include AR(1) other distributed-lag factors (for example, corAR1) and re-fit the main models to make sure that the IRR/ER is stable.*

**Response:** We thank the reviewer for their suggestion. We attempted to include an AR(1) correlation structure to account for potential residual autocorrelation. However, the model could not converge despite multiple adjustments likely due to the combination of a negative binomial family and distributed lag terms. Furthermore, aggregating to monthly counts likely reduces short-term correlations, so monthly cases are generally less strongly autocorrelated than daily cases. We have included this as a limitation in the Discussion (line 768-769):

“We were unable to include a autocorrelation structure in our model due to convergence issues arising from the combination of lagged covariates and overdispersed count outcomes.”

5. **Comment:** Province random intercepts provide for variability but fail to address spatial dependency. Examine a spatially structured effect (CAR/SAR) or spatial smooth of provincial centroids and see whether spatially informed models significantly alter IRR/ER.

**Response:** We thank the reviewer for their suggestion. We obtained Moran’s I values for each disease and each timepoint to test if there is spatial autocorrelation that needs to be addressed. For JEV, malaria, pneumonia, leptospirosis and melioidosis residuals, the Moran’s I p-values were p>0.05 for all timepoints. Hence, the spatial autocorrelation for those diseases were not statistically significant. For dengue and influenza residuals, spatial autocorrelation was significant for 143 and 63 over 212 timepoints respectively. The mean Moran’s I value across timepoints for dengue and influenza were 0.033 and 0.005 respectively, which represent very weak spatial autocorrelation. Hence, spatially informed models are unlikely to significantly alter IRR or ER.

We have revised the Methods section to justify not incorporating spatial autocorrelation into our models (lines 228-231):

“Moran’s I was calculated across diseases and timepoints. As the observed spatial autocorrelation was very weak and largely statistically non-significant, we did not incorporate spatial autocorrelation in the model.”

6. **Comment:** *In addition to AIC vs. GLM, use rolling-origin time-series cross-validation (for example, 5-fold blocks) to present RMSE/MAE and the calibration of prediction intervals for 2015–2019. This will make the projections more believable.*

**Response:** We thank the reviewer for their suggestion. We acknowledge that rolling-origin time-series cross-validation is a well-established approach for evaluating forecast performance. However, our focus was on assessing model fit and parsimony rather than short-term predictive accuracy. Hence, we adopted the Akaike Information Criterion (AIC), which provides a more appropriate balance between goodness-of-fit and model complexity. Moreover, rolling-origin validation is typically applied to short-term forecasting problems, whereas our study focuses on long-term projections over multiple years. We have added this justification to the Methods section (lines 362-364):

“Model fit was evaluated using AIC as it balances model fit with complexity and is more suitable for long-term projections than time series cross-validation approaches, which are intended for short-term forecasts.”

7. **Comment:** *You set the population at historical averages to see how weather affects it; the other option is to use estimates that are slanted towards the future population. Instead of completely getting rid of demographics, please (i) show two sets of results: risk (per-capita incidence; your main) and burden (cases using SSP-consistent population trajectories) with clear caveats, and (ii) limit the effect by age-structure scenarios or a scaled offset that is capped at historical maxima to avoid extrapolation that goes too far. This helps policymakers understand the difference between instances avoided and cases that are too high compared to risk.*

**Response:** We thank the reviewer for their suggestion. We projected disease burden accounting for SSP-consistent population trajectories but found that predictions were heavily skewed by future population projections. These results were included as Supplementary Figure 32-38. For example, dengue risk estimates were implausibly large with extremely wide confidence intervals. This is because the population in many provinces are projected to exceed historical levels, leading to extrapolation beyond the range of observed data and unstable model prediction. On the other hand, capping future population to historical maxima would underestimate burden estimates. Hence, we were unable to present disease burden under future populations. Instead, our study focuses on risk which provides a more stable and reliable measure for decision-making.

Figure S32. Excess risk of dengue attributable to extreme weather taking into account future population.

Annual excess risk of dengue in Thailand attributed to (A) all extreme weather events, (B) extreme heat days, (C) extreme dry weather and (D) extreme wet weather.

We have revised the Discussion to include this as a limitation (lines 769-772):

“We also could not account for future population growth as future population exceeded historical maxima, hence incorporating future population in projections led to implausibly large and uncertain estimates.”

8. **Comment:** *You know that malaria and JEV rely on how much the forest is disturbed and how many pigs there are, which are both left out. Dengue also relies on how well water is managed in the developed environment. To cut down on omitted-variable bias, please add province-level time-varying proxies (such forest loss, pig census, urbanization/impervious surface, and WASH indexes) or at least provincial fixed trends. Give a directed acyclic graph (DAG) that shows the assumed routes.*

**Response:** We thank the reviewer for their suggestions. We acknowledge that including variables such as forest loss and pig census are important for projecting malaria and JEV cases. However, there are currently no projections or predicted trends for these variables under different Shared Socioeconomic Pathways. Hence, we are unable to account for these in our projections. Instead, these variables are accounted for within the model intercept and is assumed to be constant. Furthermore, province-level spatial characteristics were partially accounted for through the inclusion of province-specific random effects. We have revised the discussion to include this as a limitation (lines 763-766):

“Additionally, as the focus of our study was extreme weather and due to the lack of future projected data under different SSPs, we did not account for variables such as changes in land use, use of intervention and proximity to amplifying hosts that may drive the transmission of diseases like JEV and malaria.”

9. **Comment:** *ER is calculated just during months designated as severe and is compared to historical baseline months. Because the incidence of exposure varies across SSPs, the selection of months is contingent upon the model used. Please include a counterfactual study by simulating each future month twice: once with forecast weather and once with "normal" weather based on historical monthly averages. Keep all other variables the same and report the difference. This sets up ER as a causal difference and prevents exposure from affecting it*.

**Response:** We thank the reviewer for their comment. To clarify, our study has already implemented the approach described. For each future period, we compare the incidence with future weather against incidence with “normal weather” based on historical monthly averages. We have revised the Methods to reinforce that excess risk was calculated based on this comparison (lines 339-342):

“For each month, the projected incidence under future weather conditions was compared with the expected incidence under historical average conditions for that same month. This ensures that excess risk reflects the impact of future extreme weather while holding all other covariates constant.”

10. **Comment:** *You utilise three GCMs: MIROC6 for the main one, CMCC-ESM2 for the sensitivity one, and IPSL-CM6A-LR for the sensitivity one. Think about presenting multi-model ensemble ER medians and 5–95% ranges for a larger CMIP6 subset, or explain your choice by using model performance weights for monsoon indicators in Thailand. Also, make it clear whether the MIROC6 fields were modified for bias before MODAWEC.*

**Response:** We thank the reviewer for their suggestion. We generated new projections based on a multi-model ensemble which was an average across the MIROC6, CMCC-ESM2 and IPSL-CM6A-LR models. The ensemble projections (top figure) were nearly identical to the main projections (bottom figure) used with the MIROC6 GCM.

Figure S43. Excess risk of pneumonia, influenza, JEV, malaria, dengue, melioidosis and leptospirosis during periods of extreme weather, across 2021 to 2100, in climate change scenarios SSP126, SSP245, SSP370 and SSP585 using **multi-model ensemble** climate change model.

Figure 3: Excess risk of pneumonia, influenza, JEV, malaria, dengue, melioidosis and leptospirosis during periods of extreme weather, across 2021 to 2100, in climate change scenarios SSP126, SSP245, SSP370 and SSP585 based on the **MIROC6** climate change model. Excess risk represents the percentage change in annual disease case counts from the historical baseline from 2003 to 2020.

We have included the results from the multi-model ensemble in the Results to show that using a single model produces projections consistent with an ensemble (lines 613-620):

“We estimated the excess risks attributed to extreme weather events based on 2 other global climate models (GCMs), CMCC-ESM2 and IPSL-CM6A-LR to capture the uncertainty inherent in the climate projections and test the robustness of our findings. We also estimated excess risks based on a multi-model GCM ensemble, calculated as the mean of MIROC6, CMCC-ESM2 and IPSL-CM6A-LR outputs. Across all the diseases studied, the general trends of excess risk attributable to each extreme weather scenario based on the alternative GCMs were similar to the main projections in this study (Supplementary Figures S40-48).”

Furthermore, our study did not perform additional bias correction to the MIROC6 inputs. These GCM outputs were used directly, as they already incorporate standard bias adjustments applied by the modelling centers. We have incorporated this as a limitation in the Discussion (lines 754-757):

“Moreover, we did not apply additional bias correction to the GCM inputs, so weather extremes may have been under-estimated or over-estimated, which could influence the estimated impact of extreme weather on disease outcomes.”

11. **Comment:** *The abstract says that tuberculosis is one among the illnesses, yet the Methods section contains seven diseases (dengue, JEV, malaria, pneumonia, influenza, leptospirosis, and melioidosis) that do not include TB. Please make sure that all parts and figures match up with the same set.*

**Response:** We apologise for the typographical mistake. Our study did not include any analysis on tuberculosis. We have revised the abstract to remove TB from the list of diseases (lines 25-29):

“We used generalised additive models to delineate the relationship between monthly extreme heat days, standardised precipitation index and incidence rates of seven infectious diseases (dengue, malaria, Japanese encephalitis, melioidosis, leptospirosis, pneumonia, influenza) across Thailand’s provinces.”

12. **Comment:** *Under SSP245 (central/northern provinces), you see dengue cases rise from 2021 to 2060, then fall as temperatures rise and dry/wet extremes become more common. Influenza cases rise at first, but fall as circumstances become wetter. Please provide mechanistic explanation (for example, vector survival/EIP vs. adult mortality at high Tmax; human indoor mixing and absolute humidity for influenza) and exhibit ER maps next to anticipated heat-day distributions to visibly connect mechanism and location.*

**Response:** We thank the reviewer for their suggestion. We have revised the Discussion to include mechanistic explanations in relation to the projected trends for dengue and influenza (line 697-717):

“Under SSP245, dengue is expected to first increase with moderate levels of extreme heat. This can be explained by the shortening of the extrinsic incubation period with increasing temperatures^43^. Transmission rate increases from the reduction in the time taken for *Aedes* mosquitoes to become infectious, leading to higher dengue incidence. In the second half of the century, dengue is expected to decrease in periods of extreme dry weather, extreme wet weather and high levels of extreme heat. Associations between dengue risk, extreme wet and extreme dry conditions have been observed in other studies^44^. *Aedes aegypti*, the main vector of dengue, breeds in a wide variety of natural and artificial water containers^45^. In months of drought, the number of potential breeding sites for laying eggs reduces, hence restricting the *Aedes aegypti* population and decreasing transmission of dengue. On the other hand, excessive rainfall could wash out breeding sites and hence reduce transmission of dengue^1246^.

Influenza risk is first expected to increase from historical baselines in periods of extreme heat and extreme dry weather under SSP245. This could be because physiological stress from heat and dehydration weakens respiratory immunity^47,48^. Furthermore, in Thailand, influenza cases were historically found to decline following hot and dry months^49^. These findings support our results of decreased influenza risk during extreme dry weather. Influenza is then expected to decrease later in the century during periods of extreme wet weather, despite “humid-rainy” seasons being commonly positively associated with influenza risk^49–51^. This may be a result of decreased human mobility and social mixing from extreme wet weather, reducing influenza transmission.”

We have also added additional figures in the Supplementary Information to show the ER maps against the distribution of extreme heat.

**Figure S32. Province-level distribution of extreme heat and excess risks of dengue under SSP245.**

(A) Average number of extreme heat days in a month by province from 2021-2100. (B) Province-level excess risks of dengue under SSP245 from 2021–2100. Asterisks indicate a statistically significant excess risk value. Excess risk represents the percentage change in annual disease case counts from the historical baseline from 2003 to 2020.

**Figure S33. Province-level distribution of extreme heat and excess risks of influenza under SSP245.**

(A) Average number of extreme heat days in a month by province from 2021-2100. (B) Province-level excess risks of dengue under SSP245 from 2021–2100. Asterisks indicate a statistically significant excess risk value. Excess risk represents the percentage change in annual disease case counts from the historical baseline from 2003 to 2020.

13. **Comment:** *Translate province-level ER into operational triggers (e.g., thresholds for extreme-heat days or SPI indicating ≥20% ER for dengue/influenza) and suggest specific actions (time for reducing vector sources, preparing hospitals for surges, and communicating risks) by area and decade. This will make the document more useful in real life.*

**Response:** We thank the reviewer for their suggestions. We have included a paragraph on operational triggers specific to area and time period in the Discussion (lines 678-687):

“Under the SSP245 scenario, dengue is expected to rise from 2021–2040. High-risk provinces in northern and central Thailand, particularly Bangkok, Chiang Rai, Ratchaburi, Nakhon Ratchasima, and Si Sa Ket, should prioritize vector control and hospital preparedness during heatwaves accompanied by normal rainfall, as both dry and wet extremes appear to be protective. Influenza is also expected to rise from 2021–2060 under SSP245. High-risk provinces such as Nakhon Ratchasima, Buri Ram, Ubon Ratchathani, and Chiang Mai should strengthen surveillance, vaccination campaigns, and healthcare surge capacity during heatwaves coupled with extended dry periods, which are both associated with higher transmission risks. These operational triggers may inform region-specific preparedness and response planning under future climate scenarios.”

14. **Comment:** *The table/methods only mention SSP470 once; in other places, you use SSP370. Please always fix things.*

**Response:** We apologise for the typographical mistake. We have revised the Methods to reflect the correct numbering (lines 192-193):

“In this study, the SSP126, SSP245, SSP370 and SSP585 scenarios were considered, which are summarised in Table 1.”

15. **Comment:** *You choose lags based on the average AIC of all the models for an illness. Briefly explain why the chosen delays (such dengue EIP/hatching windows and influenza incubation/transmission) make sense from a biological point of view, and provide an extra lag-response figure.*

**Response:** We thank the reviewer for their suggestion. We have included a brief biological reasoning for the chosen lags in our model (lines 235-244):

“For dengue and JEV, lags of up to 2-3 months for extreme heat and SPI reflect their effects on the extrinsic incubation period and vector reproduction, together with the delayed effects on vector abundance ^42,43^. For malaria, the short heat lag of 1 month aligns with the effect of extreme heat on the extrinsic incubation period, while rainfall effects on vector habitats accumulate over a longer period of 3 months^44,45^. For pneumonia, leptospirosis and influenza, 3-month lags for both extreme heat and SPI capture delayed effects of temperature and moisture on transmission, exposure pathways and host susceptibility^46,47^. For pneumonia and influenza, the longer lags compared to the incubation periods may be the cumulative effects of extreme weather on respiratory immunity^46,48^. Melioidosis included an immediate heat effect and a 3-month SPI lag to capture delayed rainfall impacts on soil and bacterial exposure^19^.”

We have also included the lag-response curve in the supplementary information:

Figure S25. Lag–response curves showing the relative risk of seven infectious diseases (dengue, JEV, influenza, pneumonia, leptospirosis, melioidosis, and malaria) per 1-unit increase in standardized precipitation index (SPI) and extreme heat. Relative risk (RR) represents the multiplicative change in disease incidence associated with a 1-unit increase in SPI or extreme heat at each lag compared to when all covariates are held at the mean.

16. **Comment:** *You include RH at the same time. Think about measuring absolute humidity (which is more directly related to how easily the flu spreads) and lagged RH for strength; summarise in the Supplement.*

**Response:** We thank the reviewer for the suggestion. While absolute humidity (AH) is mechanistically relevant for influenza transmission, projected AH values were not available from the global circulation models (GCMs) used in our study. Although in principle AH can be derived from relative humidity (RH) and temperature, this would propagate additional uncertainty in the model. Furthermore, our study focuses on the impacts of extreme heat and precipitation rather than on isolating the effects of humidity. The existing inclusion of RH and temperature in the model also captures the main climatic influences on all the diseases studied. Therefore, we do not anticipate that the exclusion of AH will affect our results.

17. **Comment:** Make it clear whether monthly smooths are cyclical (January ≈ December). If not, think of a cyclic spline.

**Response:** We thank the reviewer for this suggestion. In our model, months were fitted using a thin plate spline basis which does not impose a strict periodic boundary constraint. The fitted smooth from a thin plate spline closely approximated a cyclic pattern, where the estimated smooth for Month transitions smoothly between December and January. Moreover, using a cubic cyclic spline basis fitted a nearly identical smooth function.

Figure B. Fitted monthly smooths using cubic cyclic spline basis (left) and thin plate spline basis (right).

Therefore, we retained the thin plate spline specification, as it adequately represents the observed seasonality without requiring an explicit cyclic constraint. We have revised the Methods section to clarify that the fitted monthly smooths were cyclical (lines 221-223):

“Although the splines for calendar month were not explicitly specified as cyclic, the fitted monthly smooths naturally exhibited a cyclical pattern, appropriately capturing seasonality.”

18. **Comment:** *When looking at IRR over a lot of SPI/heat values and provinces, keep in mind how you show uncertainty (emphasis on impact sizes and CIs instead of binary significance).*

**Response:** We thank the reviewer for their suggestion. We have reworded the Results section to emphasis impact sizes and Cis instead of binary significance (lines 416-433):

“In dengue, JEV, influenza, pneumonia and melioidosis, the number of extreme heat days in a month was positively associated with disease risk (Fig. 2). On the other hand, increasing days of extreme heat days in a month was negatively associated with malaria and leptospirosis risk (Fig. 2). The pattern was also consistent when the association of disease incidence against extreme weather was broken down on a province-specific basis, particularly in highly populated provinces like Bangkok, Nakhon Ratchasima and Ubon Ratchathani (Supplementary Figure S2–10). Province-level PAFs demonstrated the same trend, with positive values signifying that a proportion of cases in the population can be attributed to extreme heat, extreme dry weather and extreme wet weather respectively (Supplementary Figure S11–17).

Extreme wet weather (i.e. SPI $\geq$1.5) was historically associated with lower incidence of dengue and higher incidence of JEV, pneumonia and melioidosis. At SPI levels of 3, dengue IRR was 0.66 (95% CI: 0.62, 0.69), while JEV IRR was 1.29 (95% CI: 1.19, 1.40). Extreme dry weather (i.e. SPI $\leq$ -1.5) was historically associated with higher incidence of JEV and lower incidence of dengue and pneumonia (Fig. 2). At SPI levels of $-$3, dengue IRR was 0.88 (95% CI: 0.83, 0.92), while JEV IRR was 1.21 (95% CI: 1.12, 1.30). Province-level IRRs and PAFs were concordant with these trends (Supplementary Figure S2-17).”

19. **Comment:** *Fig. 1: To indicate spread, add units (days, SPI unitless) and display distributions (like boxplots) for each time.*

**Response:** We thank the reviewer for their comment. Figure 1 is a descriptive summary of past and future monthly SPI and extreme heat days. The future values are point estimates derived from the global climate model outputs, spatially averaged over each province. Thus, there is no distribution that can be shown for the past and future climate data.

20. **Comment:** *Make sure the colour scales are the same and include the number of severe months that went into each ER estimate (this helps make sense of broad CIs).*

**Response:** We thank the reviewer for their comment. There were sufficient data points (i.e. number of months with either type of extreme weather per SSP and time period) that went into each ER estimate as shown in the following table. Each disease has the same number of months with extreme weather.

| Period | SSP126 | SSP245 | SSP370 | SSP585 |
| --- | --- | --- | --- | --- |
| 2021-2040 | 36 | 35 | 37 | 36 |
| 2041-2060 | 33 | 36 | 26 | 30 |
| 2061-2080 | 33 | 33 | 37 | 31 |
| 2081-2100 | 32 | 35 | 36 | 40 |

Broad confidence intervals (CIs) of excess risk estimates were more likely due to low case numbers, particularly in Japanese encephalitis and melioidosis which have zero-inflated case counts. We have included this in the Results to explain why some ER estimates had large CIs (lines 520-521):

“Broad confidence intervals could be explained by low monthly case counts across provinces.”

21. **Comment:** *When you state "increase/decrease during periods of extreme weather," make it clear which event (hot, dry, or wet) you mean to prevent confusion, unless you're using the combined term.*

**Response:** We thank the reviewer for their comment. We have ensured that throughout the results section, the phrasing “during periods of extreme weather” refers only to combined periods of extreme dry weather, extreme wet weather or extreme heat.

22. **Comment:** *It's great that you provide a link to GitHub. Please pin the code to a release/tag, add an environment file, and put the processed province-level series in a data repository with a DOI.*

**Response:** We thank the reviewer for their suggestion. We have pinned the code to a release in GitHub, added an environment file (<https://github.com/EstherChoo/THextremeweather>) and placed the processed data in a repository (10.6084/m9.figshare.30579395).

23. **Comment:** *To make it easier to read, some sentences could be shortened (for example, there are two statements about flu rates going down in the second half of the century—merge and cross-reference the figure panels).*

**Response:** We thank the reviewer for their suggestion. We have shortened some sentences throughout the Results section to increase readability.

24. **Comment:** *Briefly explain how you dealt with any gaps in case definition stability (for example, how you handled the province split/merge that was already mentioned).*

**Response:** We thank the reviewer for their suggestion. As Bueng Khan province was separated from Nong Khai in 2011, we added case counts from Bueng Khan back to Nong Khai from 2011 onwards. Furthermore, throughout the study period, case definitions did not change and cases were reported based on clinical suspicion. We have revised the Methods to highlights this (lines 124-125):

“There were no documented changes in case definitions for the diseases of interest between 2003 and 2019, and cases were reported based on clinical suspicion.”

25. **Comment**: *After fitting the GAMs, did you check for residual autocorrelation and spatial dependence? What did Moran's I and the residual indicate if so?*

**Response:** We thank the reviewer for their suggestion. We obtained Moran’s I values for each disease and each timepoint to test if there is spatial autocorrelation that needs to be addressed. For JEV, malaria, pneumonia, leptospirosis and melioidosis residuals, the Moran’s I p-values were p<0.05 for all timepoints. Hence, the spatial autocorrelation for those diseases were not statistically significant. For dengue and influenza residuals, spatial autocorrelation was significant for 143 and 63 over 212 timepoints respectively. The mean Moran’s I value across timepoints for dengue and influenza were 0.033 and 0.005 respectively, which represent very weak spatial autocorrelation.

Furthermore, while residual ACF did indicate residual autocorrelation, we were unable to incorporate AR(1) correlation structure into the model. The model could not converge despite multiple adjustments likely due to the combination of a negative binomial family and distributed lag terms. Furthermore, aggregating to monthly counts likely reduces short-term correlations, so monthly cases are generally less strongly autocorrelated than daily cases. We have included this as a limitation in the Discussion (lines 768-769):

“Furthermore, we were unable to include a autocorrelation structure in our model due to convergence issues arising from the combination of lagged covariates and overdispersed count outcomes.”

26. **Comment:** *How much do your dengue/influenza ER findings change when you use provincial-specific heat thresholds instead of a nationwide threshold? For example, the 90th percentile per province.*

**Response:** We thank the reviewer for their comment. We separated Thailand by its 6 geographical regions and obtained the 90^th^ percentile Tmax for each region. Each region’s 90^th^ percentile Tmax differed by only about 1 °C from the national 90^th^ percentile Tmax which was used in the study. Therefore, our excess risk (ER) findings are unlikely to change substantially if we were to use provincial-specific heat thresholds. Furthermore, using a nationwide threshold allows the province-level ERs to be comparable.

| Region | 90^th^ percentile Tmax / °C |
| --- | --- |
| Northern | 35.21 |
| Northeastern | 35.52 |
| Central | 36.10 |
| South | 33.51 |
| East | 33.84 |
| West | 35.52 |

27. **Comment:** *Did you fix any bias in the MIROC6/CMCC/IPSL inputs (for example, by using quantile mapping) before MODAWEC? If so, please write out how you did it; if not, please talk about what this means for extremes.*

**Response:** We thank the reviewer for their question. Our study did not perform additional bias correction to the MIROC6/CMCC/IPSL inputs. These GCM outputs were used directly, as they already incorporate standard bias adjustments applied by the modelling centers. Using the raw GCM outputs means that extreme values at the local scale may be over- or under-estimated, which could affect the magnitude of projected extremes. We have incorporated this as a limitation in the Discussion (lines 754-757):

“Moreover, we did not apply additional bias correction to the GCM inputs, so weather extremes may have been under-estimated or over-estimated, which could influence the estimated impact of extreme weather on disease outcomes.”

28. **Comment:** *How many intense months did each ER cell (disease × period × SSP) get? Could sparse counts be causing broad CIs, such in the case of melioidosis under SSP585?*

**Response:** We thank the reviewer for their comment. There were sufficient data points (i.e. number of months with either type of extreme weather per SSP and time period) that went into each ER estimate as shown in the following table. Each disease has the same number of months with extreme weather.

| Period | SSP126 | SSP245 | SSP370 | SSP585 |
| --- | --- | --- | --- | --- |
| 2021-2040 | 36 | 35 | 37 | 36 |
| 2041-2060 | 33 | 36 | 26 | 30 |
| 2061-2080 | 33 | 33 | 37 | 31 |
| 2081-2100 | 32 | 35 | 36 | 40 |

Broad confidence intervals (CIs) of excess risk estimates were more likely due to low case numbers, particularly in Japanese encephalitis and melioidosis which have zero-inflated case counts. We have included this in the Results to explain why some ER estimates had large CIs (lines 520-521):

“Broad confidence intervals could be explained by low monthly case counts across provinces.”

29. **Commen**t**:** *For each SSP, can you make a "normal weather" projection series that shows ER as a difference in anticipated incidence while keeping weather around historical means?*

**Response:** We thank the reviewer for their comment. To clarify, our study has already implemented the approach described. For each future period, we compare the incidence with future weather against incidence with “normal weather” based on historical monthly averages. We have revised the Methods to reinforce that excess risk was calculated based on this comparison (lines 339-342):

“For each month, the projected incidence under future weather conditions was compared with the expected incidence under historical average conditions for that same month. This ensures that excess risk reflects the impact of future extreme weather while holding all other covariates constant.”

30. **Commen**t**:** *Did model calibration or diagnostics take into account any surveillance artefacts, such COVID-19 interference around 2020 or 2009 H1N1 for influenza?*

**Response:** We thank the reviewer for their question. We did not explicitly account for such surveillance artefacts. However, our analysis uses a long time series spanning 2003 to 2019, which does not overlap with the COVID-19 pandemic and therefore avoids COVID-related reporting disruptions. Regarding influenza, while the 2009 H1N1 pandemic may have temporarily increased reported cases, these events were relatively short compared to the overall study period, Hence, their impact on long-term trends is likely minimal. We also conducted model diagnostics to ensure reasonable fit to observed data across the full time series, which did not indicate major artefacts affecting model performance. We have revised the Discussion to highlight these points (lines 774-777):

“Short-term events, such as influenza pandemics or other disruptions to surveillance, may have temporarily affect reported case counts. However, their impact on long-term trends is likely minimal given the length of the study period.”
