# Supplementary material for: Projecting long-term excess risks of major infectious diseases associated with future extreme weather events in Thailand
Source: PLoS Negl Trop Dis. 2026 Jan 5;20(1):e0013896. doi: 10.1371/journal.pntd.0013896 (PMC12782439; doi:10.1371/journal.pntd.0013896)
Supplement: Attachment — Submitted filename: 2025 PloS NTD_projections_rev2.docx [file pntd.0013896.s062.docx]

In the Research Article, authors first use GAMs to describe the historical relationships between the number of extreme heat days in a month and extreme dry and extreme wet conditions (as measured by SPI) and a number of disease outcomes in Thailand. These diseases include dengue, JEV, influenza, malaria, pneumonia, leptospirosis, and meliodosis. They then apply the exposure-response relationships to projected heat and SPI metrics to estimate the additional number of cases caused or averted by extreme weather at mid- and end-of-century. The paper is ambitious, and the authors should be commended on how much work went into this paper.

The authors have addressed many of the comments of the reviewers, although I feel that there remains room for improvement in terms of simplifying the presentation of figures and results (especially Figs 3, 6, and 7). I have a few minor comments after reviewing the revised version:

**Comment:** I appreciate the clarification of model terminology. In the revised manuscript, the authors state:

$x_{1,t-k}$ is the SPI up to $k=\{1,2,..,p\}$lags, $x_{2,t-j}$ is the number of extreme heat days in a month up to $j=\{1, 2,...,q\}$lags

Since you modelled lag 0 too, should this be $k=\{0, 1,2,..,p\}$ and $j=\{0,1, 2,...,q\}$?

**Comment:** In revised equation 4, the authors state:

$$\hat{y}_{proj,d,m, t,l,s}=E\left[ \hat{y}_{d,m,t,l,s} | x_{i}=x_{i,m,t,l,s}^{*},\left\{ x_{1, m-k,t,l,s}^{*} | 1\leq k\leq p \right\},\left\{ x_{2,m-q,t,l,s}^{*} | 1\leq j\leq q \right\},x_{-i}=\bar{x_{-i,m,l}} \right]$$

(4)

$x_{1, m-k,t,l,s}^{*}$ is the lagged monthly extreme heat days of up to $p$ lags, while $x_{2, m-q,t,l,s}^{*}$ is the lagged monthly extreme heat days of up to $q$ lags.

In accordance with the notation above (see comment 1), should the q in the subscript for x_2_^*^ be a j: $x_{2, m-j,t,l,s}^{*}$ is the lagged monthly extreme heat days of up to $q$ lags. Additionally, is it possible to combine

**Comment:** In the discussion and results, it is curious that dengue is expected to decrease with extreme heat given the IRRs in Figure 2 show only increases. The authors explain that the projected decreases in dengue are largely attributed to changes in SPI, which makes sense. However, Figure 4A does show decreases in dengue with higher heat (with one exception). My best guess for why dengue is projected to decrease with heat is that the exposure-response curve (Fig 2) starts to decrease for increases in heat days between ~16 and 22. Do the authors think this shape is reflective of a true mechanistic relationship between dengue and extreme heat, or could it be an artifact of the model being overly flexible?

**Comment:** I appreciate the addition of the lag-response relationships in Figure S25. Although the lag duration was chosen using AIC, the relationship between extreme weather and IDs generally do not return to null by the end of the lagged period. This suggests that the full, delayed effects of extreme weather on disease transmission may not be entirely accounted for in the analysis. The authors may wish to comment on this in the discussion.
