# Supplementary material for: Projecting long-term excess risks of major infectious diseases associated with future extreme weather events in Thailand
Source: PLoS Negl Trop Dis. 2026 Jan 5;20(1):e0013896. doi: 10.1371/journal.pntd.0013896 (PMC12782439; doi:10.1371/journal.pntd.0013896)
Supplement: Attachment — Submitted filename: Response_to_Reviewers_auresp_2.docx [file pntd.0013896.s063.docx]

PNTD-D-25-00718

Response to reviewers

Dear Dr. Shaden Kamhawi, Dr Paul Brindley and Dr Peter J. Hotez,

Thank you for the opportunity to submit a revised draft of the manuscript entitled “Projecting long-term excess risks of major infectious diseases associated with future extreme weather events in Thailand” to *PLOS Neglected Tropical Diseases*. We appreciate the time and effort that you and the reviewers have dedicated to providing your valuable feedback on my manuscript. We have been able to incorporate changes to reflect the suggestions provided by the reviewers and have tracked the changes within the manuscript. Here is a point-by-point response to the reviewers’ comments and concerns.

Comments from Reviewer 1

1. **Comment:** *The authors have addressed many of the comments of the reviewers, although I feel that there remains room for improvement in terms of simplifying the presentation of figures and results (especially Figs 3, 6, and 7).*

**Response:** We thank the reviewer for their comment. We have simplified the presentation of Figures 3, 6 and 7. In Figure 3, we have only retained text labels of statistically significant points to avoid overlapping text boxes. In panel A of Figures 6 and 7, we have reduced the number of provinces highlighted from 10 to 5 to improve clarity and reduce overlapping lines:

**Figure 3**: Excess risk of pneumonia, influenza, JEV, malaria, dengue, melioidosis and leptospirosis during periods of extreme weather, across 2021 to 2100, in climate change scenarios SSP126, SSP245, SSP370 and SSP585. Excess risk represents the percentage change in annual disease case counts from the historical baseline from 2003 to 2020.

**Figure 6**: (A) Excess risk of the five provinces with the historically highest incidence of dengue during periods of extreme weather, under SSP245 from 2021–2100. (B) Province-level excess risks of dengue under SSP245 from 2021–2100. Asterisks indicate a statistically significant excess risk value. Excess risk represents the percentage change in annual disease case counts from the historical baseline from 2003 to 2020. Map created using GADM data (<https://gadm.org/index.html>, freely available for academic use). The map outlines and administrative boundaries are used with permission for academic publishing.

**Figure 7**: (A) Excess risk of the five provinces with the historically highest incidence of influenza during periods of extreme weather, under SSP245 from 2021–2100. (B) Province-level excess risks of influenza under SSP245 from 2021–2100. Asterisks indicate a statistically significant excess risk value. Excess risk represents the percentage change in annual disease case counts from the historical baseline from 2003 to 2020. Map created using GADM data (<https://gadm.org/index.html>, freely available for academic use). The map outlines and administrative boundaries are used with permission for academic publishing.

2. **Comment:** *I appreciate the clarification of model terminology. In the revised manuscript, the authors state:*

$x_{1,t-k}$ *is the SPI up to* $k=\{1,2,..,p\}$*lags,* $x_{2,t-j}$ *is the number of extreme heat days in a month up to* $j=\{1, 2,...,q\}$*lags*

*Since you modelled lag 0 too, should this be* $k=\{0, 1,2,..,p\}$ *and* $j=\{0,1, 2,...,q\}$*?*

**Response:** We thank the reviewer for their comment. We have revised the equation to include lag 0 (lines 217-218):

“$x_{1,t-k}$ is the SPI up to $k=\{0, 1,2,..,p\}$lags, $x_{2,t-j}$ is the number of extreme heat days in a month up to $j=\{0, 1, 2,...,q\}$ “

3. **Comment:** *In revised equation 4, the authors state:*

$$\hat{y}_{proj,d,m, t,l,s}=E\left[ \hat{y}_{d,m,t,l,s} | x_{i}=x_{i,m,t,l,s}^{*},\left\{ x_{1, m-k,t,l,s}^{*} | 1\leq k\leq p \right\},\left\{ x_{2,m-q,t,l,s}^{*} | 1\leq j\leq q \right\},x_{-i}=\bar{x_{-i,m,l}} \right]$$

*(4)*

$x_{1, m-k,t,l,s}^{*}$ *is the lagged monthly extreme heat days of up to* $p$ *lags, while* $x_{2, m-q,t,l,s}^{*}$ *is the lagged monthly extreme heat days of up to* $q$ *lags.*

*In accordance with the notation above (see comment 1), should the q in the subscript for x_2_^*^ be a j:* $x_{2, m-j,t,l,s}^{*}$ *is the lagged monthly extreme heat days of up to* $q$ *lags. Additionally, is it possible to combine*

**Response:** We thank the reviewer for their comment. We have corrected the subscript in Equation 4 (lines 314-317):

“$\hat{y}_{proj,d,m, t,l,s}=E\left[ \hat{y}_{d,m,t,l,s} | x_{i}=x_{i,m,t,l,s}^{*},\left\{ x_{1, m-k,t,l,s}^{*} | 1\leq k\leq p \right\},\left\{ x_{2,m-j,t,l,s}^{*} | 1\leq j\leq q \right\},x_{-i}=\bar{x_{-i,m,l}} \right]$

(4)”

4. **Comment:** *In the discussion and results, it is curious that dengue is expected to decrease with extreme heat given the IRRs in Figure 2 show only increases. The authors explain that the projected decreases in dengue are largely attributed to changes in SPI, which makes sense. However, Figure 4A does show decreases in dengue with higher heat (with one exception). My best guess for why dengue is projected to decrease with heat is that the exposure-response curve (Fig 2) starts to decrease for increases in heat days between ~16 and 22. Do the authors think this shape is reflective of a true mechanistic relationship between dengue and extreme heat, or could it be an artifact of the model being overly flexible?*

**Response:** We thank the reviewer for their comment. In response, we conducted additional analyses to investigate the discrepancy between the projected decrease in dengue risk during extreme heat and the historically observed positive association. We found that the projected changes in dengue risk were primarily driven by relative humidity rather than extreme heat alone, which explains the unexpected decrease in dengue with extreme heat. Specifically, when relative humidity was held constant between the baseline and projection scenarios, dengue excess risk during extreme heat was small and statistically insignificant across all SSPs and time periods (Supplementary Figure 26).

In contrast, incorporating projected changes in relative humidity resulted in a marked decline in dengue risk, indicating that lower relative humidity levels under future extreme heat conditions play a key role in shaping these projections (Supplementary Figure 27). We have revised the results to highlight the role of relative humidity in the projected decreases in dengue risk (lines 470-480):

“A decrease in dengue is expected during periods of extreme heat under all climate change scenarios other than SSP245 (Fig. 4A). In SSP245, a large increase of 26.2% (95% CI: 0.90%, 51.7%) is expected from 2021–2040, which then declines and increases slightly again in 2081–2100 (Fig. 4A). The decrease in dengue risk during extreme heat generally contradicts the historical association between extreme heat and increased dengue risk. We found that changes in dengue risk were primarily driven by future relative humidity. When relative humidity was held constant between the baseline and projection scenarios, dengue excess risk was small and statistically insignificant across all SSPs and time points (Supplementary Figure 26). This suggests that the projected decrease in dengue risk was likely driven by lower levels of relative humidity, which have been historically associated with reduced dengue risk (Supplementary Figure 27). Among other diseases, relative humidity was not found to drive any changes in excess risk during periods of extreme heat (Supplementary Figure 26).”

To further support this interpretation, we have added a mechanistic explanation in the Discussion (lines 743–745):

“Decrease in dengue risk can also be driven by a decrease in relative humidity. Dengue transmission is affected by lower relative humidity, which can shorten the lifespan of mosquitoes and reduce oviposition rates^57,58^.”

**Figure S26. Projected excess risk of diseases in extreme heat when relative humidity is held constant.**

Excess risk associated with extreme heat under scenarios holding relative humidity constant (RH) (left panels) and using projected RH (right panels) for seven infectious diseases. Estimates are shown across climate change scenarios and time periods. Excess risk represents the percentage change in disease cases compared to historical levels. Holding RH constant results in minimal and non-significant dengue excess risk during extreme heat, suggesting that RH drives projected changes in dengue risk during periods of extreme heat.

**Figure S27. Incidence rate ratio of diseases across different levels of relative humidity.**

Figures show incidence rate ratio (IRR) of each disease over relative humidity (RH). The IRR gives the ratio of predicted cases with the respective RH to predicted cases with mean RH of 75%, obtained from the disease-specific generalised additive models. An IRR above 1 represents increased incidence rate when relative humidity exceeds the mean, while an IRR below 1 represents decreased incidence rate. Points represent point estimates for the IRR with accompanying 95% confidence intervals. IRRs were derived using the ratio of predicted cases with exposure and predicted cases with mean exposure. Statistical significance was denoted with orange points when 95% CIs of the IRRs do not cross 1.

5. **Comment:** *I appreciate the addition of the lag-response relationships in Figure S25. Although the lag duration was chosen using AIC, the relationship between extreme weather and IDs generally do not return to null by the end of the lagged period. This suggests that the full, delayed effects of extreme weather on disease transmission may not be entirely accounted for in the analysis. The authors may wish to comment on this in the discussion.*

**Reviewer:** We thank the reviewer for their comment. The optimal lag duration of up to 4 months was chosen based on AIC. We limited the lag duration of up to 4 months as this adequately accounts for short-term and medium-term impacts of extreme weather on disease transmission, including biologically processes such as vector population dynamics and pathogen incubation periods. Adding lags beyond 4 months may increase the risk of capturing spurious associations. Hence, the chosen lag durations provide sufficient temporal coverage without compromising model reliability and interpretability.
